# Autism-related traits in myotonic dystrophy type 1 model mice are due to MBNL sequestration and RNA mis-splicing of autism-risk genes

**DOI:** 10.1038/s41593-025-01943-0

**Published:** 2025-04-21

**Authors:** Łukasz J. Sznajder, Mahreen Khan, Adam Ciesiołka, Mariam Tadross, Curtis A. Nutter, Katarzyna Taylor, Christopher E. Pearson, Mark H. Lewis, Rochelle M. Hines, Maurice S. Swanson, Krzysztof Sobczak, Ryan K. C. Yuen

**Affiliations:** 1 Department of Chemistry and Biochemistry, University of Nevada, Las Vegas, NV USA; 2 Department of Molecular Genetics and Microbiology, Center for NeuroGenetics and the Genetics Institute, University of Florida, College of Medicine, Gainesville, FL USA; 3 Department of Molecular Genetics, University of Toronto, Toronto, Ontario Canada; 4 Genetics and Genome Biology, The Hospital for Sick Children, Toronto, Ontario Canada; 5 Department of Gene Expression, Institute of Molecular Biology and Biotechnology, Adam Mickiewicz University, Poznań, Poland; 6 Department of Psychiatry, McKnight Brain Institute, University of Florida, College of Medicine, Gainesville, FL USA; 7 Department of Psychology, University of Nevada, Las Vegas, NV USA; 8 Centre for Applied Genomics, The Hospital for Sick Children, Toronto, Ontario Canada

**Keywords:** Transcriptomics, RNA splicing, Social behaviour, Autism spectrum disorders

## Abstract

Genome-wide enrichment of gene-specific tandem repeat expansions has been linked to autism spectrum disorder. One such mutation is the CTG tandem repeat expansion in the 3′ untranslated region of the *DMPK* gene, which is known to cause myotonic muscular dystrophy type 1. Although there is a clear clinical association between autism and myotonic dystrophy, the molecular basis for this connection remains unknown. Here, we report that sequestration of MBNL splicing factors by mutant DMPK RNAs with expanded CUG repeats alters the RNA splicing patterns of autism-risk genes during brain development, particularly a class of autism-relevant microexons. We demonstrate that both *DMPK*-CTG expansion and *Mbnl* null mouse models recapitulate autism-relevant mis-splicing profiles, along with social behavioral deficits and altered responses to novelty. These findings support our model that myotonic dystrophy-associated autism arises from developmental mis-splicing of autism-risk genes.

## Abstract (web summary)

Sznajder et al. identified a molecular link between autism and myotonic dystrophy, showing that a tandem repeat mutation in a single gene can disrupt splicing of multiple autism-related genes during brain development, leading to autism-like traits.

## Main

Autism spectrum disorder (ASD) is a genetically and clinically heterogeneous neurodevelopmental condition that affects communication and social interactions with restricted interests and repetitive behaviors^1^. ASD affects 1 in 36 children, and more than 95% of them have at least one additional physical or mental health condition^2,3^. Despite hundreds of genes known to confer risk for ASD, the molecular mechanisms explaining ASD and comorbid conditions remain elusive^4^.

Recent whole-genome sequencing studies identified tandem repeat mutations that contribute to ASD risk^5,6^. One of the most recurrent mutations includes a CTG expansion (CTG^exp^), defined as >50 CTG repeats, in the 3′ untranslated region of the DM1 protein kinase (*DMPK*) gene. The prevalence of this mutation is estimated at ~1:2,100 newborns^7^. The *DMPK*-CTG^exp^ mutation causes myotonic dystrophy type 1 (DM1), a neuromuscular disease with onset times that span from in utero to late adulthood and highly variable symptom severity^8^. Previous studies reported comorbidity of DM1 and ASD and showed that the presence of ASD associates with younger age of DM1 onset^9–13^; however, a molecular mechanism explaining the manifestation of ASD in DM1-affected individuals is unknown.

In DM1, DMPK-CUG^exp^ transcripts provide many high-affinity binding sites for muscleblind-like (MBNL) RNA-binding proteins (RBPs), resulting in MBNL sequestration and formation of biomolecular condensates known as RNA foci^14,15^. MBNL proteins, including MBNL1 and MBNL2, are *trans*-acting factors that regulate alternative splicing (AS) during embryonic stem cell pluripotency and reprogramming, cell type differentiation and maturation, and organ development^16–18^. In postnatal tissues, MBNL loss leads to ‘adult-to-fetal’ reversion of the AS program, resulting in a plethora of DM1 clinical symptoms^19,20^. The severity of symptoms in DM1 corresponds well with the CUG^exp^ length, the concordant sequestration level of MBNL paralogs and the degree of mis-splicing^21,22^.

Developmental mis-splicing is a feature of DM1 and ASD^23–25^. Previous studies have reported the involvement of RBFOX and SRRM4 RBPs in mis-splicing of ASD-risk genes^26,27^. While RBFOX regulates multiple types of AS, SRRM4 predominantly governs the inclusion of neuronal microexons (miEs) of 3 to ~30 nucleotides (nt) that are misregulated in ~30% of idiopathic ASD brains^26^. MiEs play an essential role in nervous system development by encoding post-translational modification sites and modulating protein–protein interaction networks^28,29^. Alterations that recapitulate neuronal miE mis-splicing can lead to ASD-like behavioral phenotypes in mice, including social avoidance^30,31^. Although numerous DM1 mouse models have been investigated, the mechanistic pathway underlying ASD traits remains unknown^32,33^.

Here, we provide a mechanistic understanding of autism via the CUG^exp^ repeat in DM1. We demonstrate that loss of MBNL proteins leads to splicing disruptions, ~17–25% of which occur within known ASD-risk genes, including multiple top ASD-risk genes such as *SCN2A* and *ANK2*. We find that neuronal miEs constitute a novel class of mis-spliced events in DM1. Sequestration of MBNL proteins recapitulates miE mis-splicing of ASD-risk genes caused by SRRM4 protein downregulation in idiopathic ASD. Our detailed mechanistic analysis of the ANK2 miE demonstrates synergistic regulation by MBNL and SRRM proteins. Finally, we demonstrated social behavior deficits using both *Dmpk*-CTG^exp^ knock-in (KI) and *Mbnl2* knockout (KO) mouse models. Our results suggest that DM1-associated ASD is caused by developmental mis-splicing of ASD-linked genes, arising from loss of MBNL activity due to CUG^exp^.

## Results

### Mis-splicing of ASD-risk genes in DM1 prefrontal cortex

**Fig. 1:**
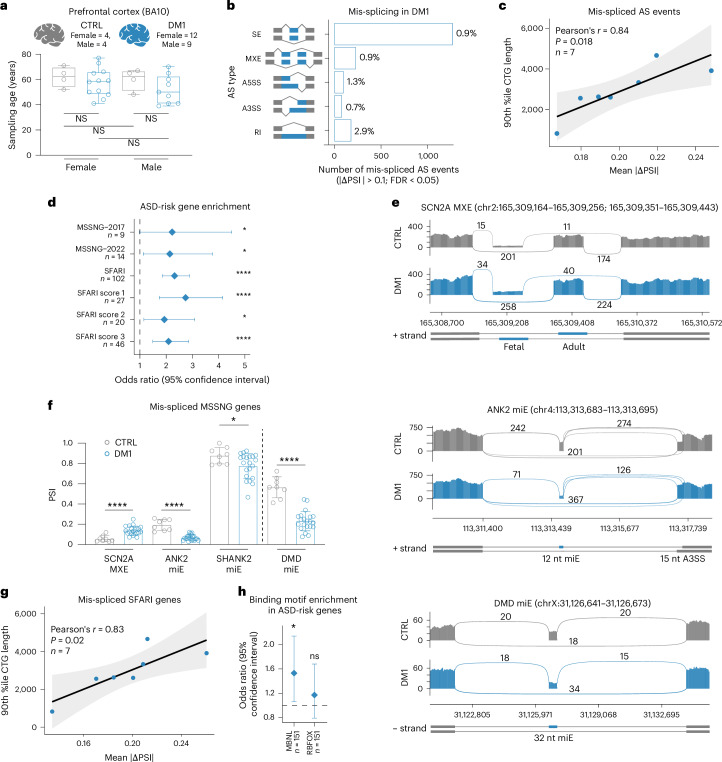
ASD-risk gene mis-splicing in human DM1 prefrontal cortex. **a**, Age and sex distribution of DM1 and control (CTRL) prefrontal cortex samples used by Otero et al.^35^ to generate RNA-seq data. Box plot shows the lower, middle and upper quartiles. Whiskers show minimum and maximum. Ordinary one-way analysis of variance (ANOVA) with Tukey’s multiple comparisons test: NS, adjusted *P (P*_adj_) > 0.6. **b**, The number and percentage of AS event types significantly mis-spliced in DM1. SE, skipped exon; MXE, mutually exclusive exon; A5SS/A3SS, alternative 5′/3′ splice site; RI, retained intron event. **c**, Strong positive correlation between *DMPK*-CTG lengths and mean |ΔPSI| for all mis-spliced events. The 90th percentile of *DMPK*-CTG length was selected for this analysis, as it shows the strongest correlation between repeat sizes and splicing dysregulation score in DM1 (ref. ^35^). **d**, Enrichment analysis for mis-spliced ASD-risk gene sets in DM1. **e**, Sashimi plots quantitatively visualize splice junctions in DM1 (*n* = 8) and CTRL (*n* = 8) RNA-seq samples for selected AS events. **f**, High-confidence mis-splicing of ASD-risk genes in DM1 (*n* = 21) versus CTRL (*n* = 8). Data are presented as mean values PSI ± s.d. **g**, Strong positive correlation between *DMPK*-CTG lengths and mean |ΔPSI| for mis-spliced events in SFARI genes in DM1. **h**, MBNL and RBFOX binding motif enrichment near SE in mis-spliced autism-risk genes. **c**,**g**, The black diagonal line is the linear regression line, and the light gray area is the 95% confidence interval. *P* value for a two-tailed test. **d**,**h**, Diamonds represent the OR. Error bars depict the 95% confidence interval. **d**,**f**,**h**, NS, FDR = 0.38, *FDR < 0.05, ****FDR < 0.0001. Source data

**Extended Data Fig. 1:**
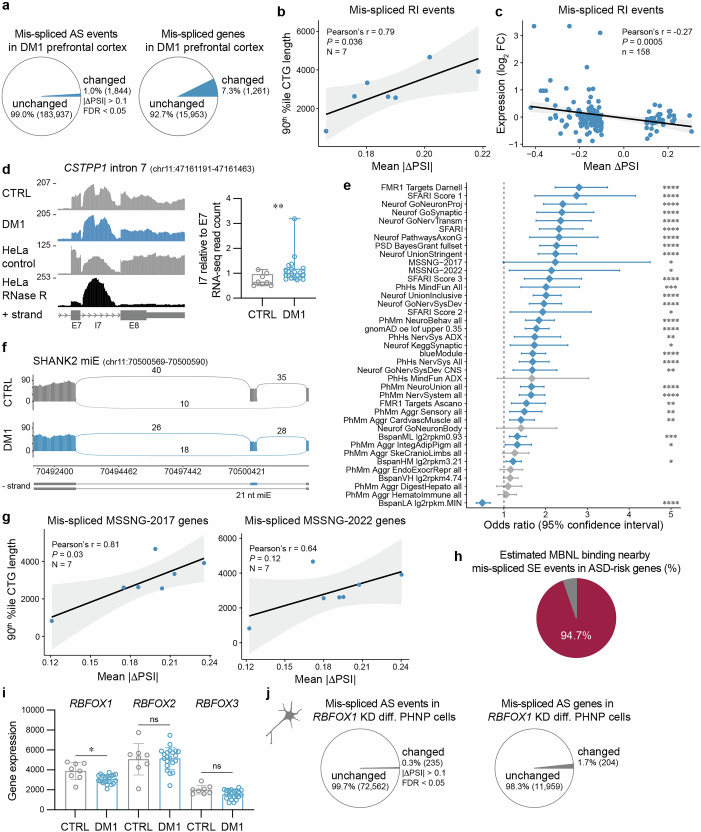
Mis-splicing in DM1 prefrontal cortex. **a**, Differential AS analysis in DM1 (N = 21) compared to control (CTRL; N = 8) prefrontal cortex. The percentage of mis-spliced AS events (left) and genes (right) in the DM1. **b**, Strong positive correlation between *DMPK*-CTG lengths and mean |ΔPSI| for mis-spliced retained intron (RI) events. Note, the 90^th^ percentile of *DMPK*-CTG length was selected, since it displays the strongest correlation between repeat sizes and splicing dysregulation score in DM1^35^. The black diagonal line represents the linear regression line, and the light gray area indicates the 95% confidence interval. P-value for a two-tailed test. **c**, Negative correlation between mean ΔPSI for mis-spliced RI events (n = 158) and their host transcript relative steady-state levels (baseMean ≥ 1) in DM1. FC-fold change. The black diagonal line represents the linear regression line, and the light gray area indicates the 95% confidence interval. P-value for a two-tailed test. **d**, Circular RNA species in CSTPP1 intron 7 (I7) in DM1 (N = 21) and CTRL (N = 8) prefrontal cortex as well as control and HeLa treated with RNase R RNA-seq samples^35,92^. Box plot shows I7 relative to exon 7 (E7) RNA-seq read count. Box plot shows the lower, middle, and upper quartiles. Whiskers show minimum and maximum. Two-tailed Mann-Whitney test; **P = 0.0098. **e**, ASD-relevant gene set enrichment analysis for mis-spliced genes in DM1 (N = 21). Diamonds represent the OR. Error bars depict the 95% confidence interval. *FDR < 0.05, **FDR < 0.01, ****FDR < 0.0001. **f**, Sashimi plots of DM1 (N = 8) and CTRL (N = 8) RNA-seq samples for SHANK2 miE. **g**, Positive correlation between *DMPK*-CTG lengths and mean |ΔPSI| for mis-spliced MSSNG-2017 and MSSNG-2022 genes in DM1. The black diagonal lines represent the linear regression lines, and the light gray areas indicate the 95% confidence intervals. P-value for a two-tailed test. **h**, Prediction of MBNL binding to mis-spliced SE events in ASD-risk genes in DM1. **i**, *RBFOX1*, *RBFOX2*, and *RBFOX3* expression in DM1 (N = 21) and CTRL (N = 8) prefrontal cortex. Data are presented as mean values ± SD. DEseq2; ^ns^*P*_adj_ > 0.05, *P_adj_ = 0.021. **j**, Differential AS analysis in *RBFOX1* KD (N = 5) compared to control (N = 5) primary human neural progenitor (PHNP) cell RNA-seq samples^52^. The percentage of mis-spliced AS events (left) and genes (right). Source data

The prefrontal cortex orchestrates executive functions, and transcriptome-wide changes are observed in this brain region of ASD-affected individuals^25,34^. To investigate whether the *DMPK*-CTG^exp^ mutation leads to RNA mis-splicing of ASD-risk genes in the prefrontal cortex, we analyzed RNA sequencing (RNA-seq) data generated from Brodmann area 10 (BA10) of human DM1 brain. Unaffected control samples were matched to minimize age and sex biases (Fig. 1a)^35–37^. For differential AS analysis, we computed the change of ‘percent spliced in’ (ΔPSI) for five AS event types (Fig. 1b). Of the ~184,000 AS events and ~16,000 genes in DM1, 1% (1,844) of AS events met our mis-splicing criteria (|ΔPSI| > 0.1, false discovery rate (FDR) < 0.05) in the total pool of 7% (1,261) of mis-spliced genes (Extended Data Fig. 1a). CTG^exp^ repeat length showed a strong positive correlation with the mean |ΔPSI| values for all mis-spliced events, including retained intron (RI) events (Fig. 1c and Extended Data Fig. 1b). Although not all RIs introduce a premature termination codon that induces nonsense-mediated decay, consistent with earlier studies, we observed a negative correlation between the level of intron inclusion and the steady-state level of the host transcript (Extended Data Fig. 1c)^38,39^. Some mis-spliced RI events represented additional RNA species, such as an elevated circular intronic RNA level in DM1 (Extended Data Fig. 1d), suggesting that other molecular events contribute to IR mis-splicing estimation.

To assess the relevance of DM1 mis-splicing to ASD, we retrieved 38 gene sets and available databases (Supplementary Table 1). We found a significant enrichment of mis-spliced events for 79% of the gene sets (Extended Data Fig. 1e) and 61% after applying more stringent mis-splicing criteria (|ΔPSI| > 0.2), indicating a consistent trend (Pearson’s *r* = 0.79, *P* < 0.0001). No enrichment was observed for the gene sets used as negative controls (such as immune or metabolism related genes). Notably, there was a significant enrichment of ASD-risk genes from the Simons Foundation Autism Research Initiative (SFARI) database and two large Autism Speaks MSSNG-based whole-genome sequencing studies, MSSNG-2017 (ref. ^40^) and MSSNG-2022 (ref. ^41^) (Fig. 1d and Extended Data Fig. 1e). Out of 36 overlapping high-confidence ASD-risk genes in both MSSNG-2017 and MSSNG-2022 studies, six were mis-spliced in DM1, including *SCN2A*, *ANK2* and *SHANK2* (Fig. 1e,f and Extended Data Fig. 1f). We also identified mis-splicing in the *DMD* gene, known to underlie Duchenne muscular dystrophy associated with ASD^42,43^. We also detected a significant positive correlation of CTG^exp^ length with mis-spliced events in ASD-risk genes (Fig. 1g and Extended Data Fig. 1g). Collectively, these results indicated that the *DMPK*-CTG^exp^ in DM1 prefrontal cortex perturbs the splicing of ASD-risk genes.

### *Mbnl* cDKO cortex mimics ASD-risk gene mis-splicing in DM1

The correlation of CTG^exp^ length with degree of ASD-risk gene mis-splicing suggests the involvement of MBNL splicing factors^35^. Based on our previous MBNL–RNA interaction studies^14,44,45^, we determined YGCYGCY and YGCY(N)_0–5_YGCY (Y indicates pyrimidine) as high-affinity MBNL-binding sequences. A genome-wide distribution analysis identified a significant enrichment of those binding motifs within 250 bp of mis-spliced skipped/cassette exons (SE) in ASD-risk genes in DM1 (FDR = 0.038) (Fig. 1h). In agreement, MBNL was also predicted to bind nearby ~95% (*P* = 0.043) of such mis-spliced events by RBPmap^46^ (Extended Data Fig. 1h).

**Fig. 2:**
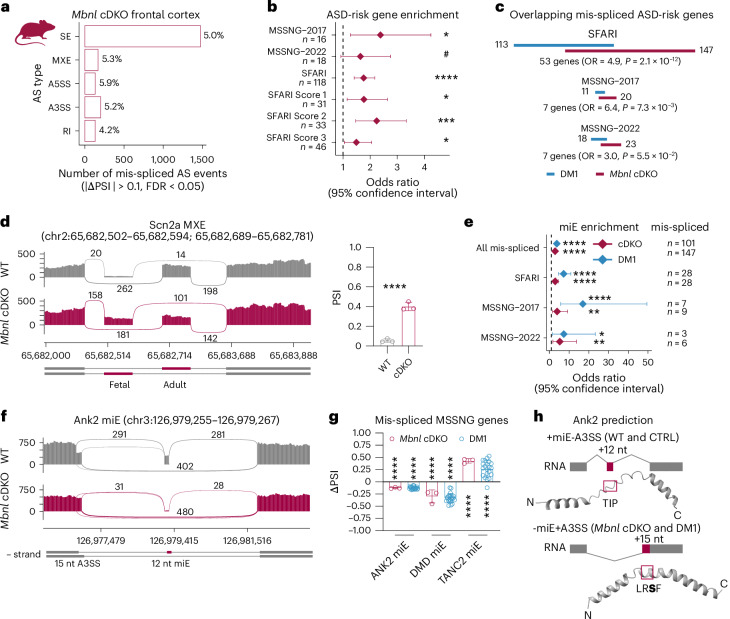
Microexon mis-splicing in DM1 and *Mbnl* cDKO frontal cortices. **a**, The number and percentage of AS event types significantly mis-spliced in *Mbnl* cDKO (*n* = 3 males). **b**, ASD-risk gene set enrichment analysis for mis-spliced genes in *Mbnl* cDKO. **c**, Overlap between mis-spliced ASD-risk gene sets in DM1 and *Mbnl* cDKO. **d**, Scn2a MXE mis-splicing in *Mbnl* cDKO (*n* = 3) versus WT (*n* = 3) RNA-seq. **e**, miE enrichment analysis for mis-spliced ASD-risk gene sets. **f**, Ank2 miE mis-splicing in in *Mbnl* cDKO RNA-seq. **g**, Orthologous mis-spliced miEs in DM1 (*n* = 21) and *Mbnl* cDKO (*n* = 3). **h**, Schematic of Ank2 miE to the A3SS coordinate splicing and modeled structures of mouse Ank2 polypeptides. The aa sequences changed by AS are shown by a magenta box. The S901 phosphorylation site is bolded. Diamonds represent OR (**b**,**e**). Error bars depict 95% confidence intervals. Data are presented as mean values ± s.d. (**d**,**g**). ^#^FDR < 0.10, *FDR < 0.05, **FDR < 0.01, ***FDR < 0.001, ****FDR < 0.0001 (**b**,**d**,**e**,**g**). Source data

**Extended Data Fig. 2:**
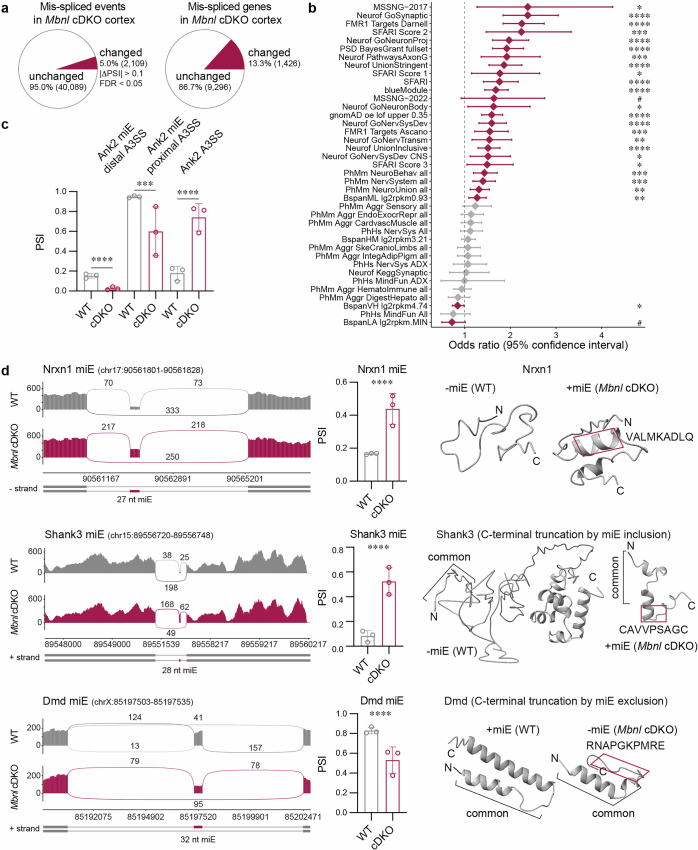
miE mis-splicing in *Mbnl* cDKO frontal cortex. **a**, Differential AS analysis in frontal cortex of *Mbnl* cDKO compared to littermate WT control (N = 3 males). The percentage of mis-spliced AS events (left) and genes (right) in the *Mbnl* cDKO. **b**, ASD-relevant gene set enrichment analysis for mis-spliced genes in *Mbnl* cDKO (N = 3). Diamonds represent the odds ratio. Error bars depict the 95% confidence interval. **c**, Quantification of coordinate Ank2 miE and A3SS mis-splicing in *Mbnl* cDKO (N = 3) and WT (N = 3) RNA-seq samples. **d**, Examples of miE mis-splicing in ASD-risk genes. Sashimi plots of *Mbnl* cDKO (N = 3) and WT (N = 3) RNA-seq samples for Nrxn1 miE, Shank3 miE, and Dmd miE. Modeled structures of mouse protein isoform regions containing a sequence encoded by miE (magenta box). **c,d**, Data are presented as mean values ± SD. **b,c,d**, ^#^FDR < 0.10, *FDR < 0.05, **FDR < 0.01, ***FDR < 0.001, ****FDR < 0.0001. Source data

To further investigate the role of MBNL in regulation of ASD-risk genes, we performed differential AS analysis on RNA-seq data from adult *Mbnl1*^−/−^;*Mbnl2*^c/c^;Nestin-*Cre*^+/−^ conditional double KO mice (hereafter *Mbnl* cDKO) frontal cortex samples^47^. *Mbnl* cDKO mice bypass the embryonic lethality of constitutive *Mbnl1*^−/−^*;Mbnl2*^−/−^ DKO mice and provide a nervous system-specific model in which *Mbnl1* expression is absent in all tissues, whereas *Mbnl2* is lost only in the nervous system, including neuronal and glial precursor cells. The *Mbnl* cDKO is characterized by RNA mis-splicing, altered cortical neuronal and synaptic structures and widespread anatomical changes in the brain^47–50^. In total, 5% (1,109) of AS events in 13% (1,426) of detected genes were mis-spliced (Fig. 2a and Extended Data Fig. 2a). Similar to human DM1, 61% of gene sets were significantly enriched among the mis-spliced genes in the *Mbnl* cDKO, including ASD-risk gene sets (Fig. 2b and Extended Data Fig. 2b). We detected significant overlaps between SFARI, MSSNG-2017 and MSSNG-2022 mis-spliced ASD-risk genes in human DM1 and mouse *Mbnl* cDKO cortices (Fig. 2c), which represent distinct systems (such as mutation type). In total, we identified a significant overlap of 55 commonly mis-spliced ASD-risk genes (odds ratio (OR) = 1.7, *P* = 1.6 × 10^−12^, Fisher’s exact test), including *SCN2A* (Fig. 2d). All these results indicate that MBNL loss perturbs ASD-risk gene splicing in the human DM1 cortex.

The RBFOX1 splicing regulator is downregulated in DM1 cortical organoids^51^, as are *RBFOX* paralogs in ASD brains^27^. Our differential gene expression analysis showed a 21% reduction of *RBFOX1* RNA level in human DM1 cortex, which did not correlate with CTG^exp^ length (*P* = 0.32) (Extended Data Fig. 1i). To explore the impact of RBFOX1 downregulation on mis-splicing of ASD-risk genes in DM1, we retrieved RNA-seq samples from differentiated primary human neural progenitor cells with *RBFOX1*-knockdown (KD)^52^. Our analysis revealed 235 mis-spliced events in the *RBFOX1* KD (Extended Data Fig. 1j). We found that less than 1% (12) of mis-spliced events in the DM1 overlapped with those observed in *RBFOX1* KD cells. The extent of this mis-splicing did not correlate with CTG^exp^ length (*P* = 0.62) and none of the overlapping events was found in ASD-risk genes. To support this observation, we performed a genome-wide distribution analysis of the RBFOX1-binding motif (GCAYG) and failed to detect significant enrichment of RBFOX1-binding sequences in mis-spliced ASD-risk genes in DM1 (FDR = 0.38) (Fig. 1h). Further, no significant RBFOX1 binding was predicted by RBPmap software in nearby mis-spliced events (*P* = 0.29). We concluded that mis-splicing of ASD-risk genes in the DM1 cortex is primarily attributed to MBNL loss, as evidenced by the lack of involvement of RBFOX1.

### Microexon mis-splicing of ASD-risk genes in DM1 and *Mbnl* cDKO

Mis-splicing of neuronal miEs (defined here as a 3–33 bp SE), a hallmark of ASD brains, can lead to ASD-like behaviors in mice^26,30,31^. We noticed significant enrichment of miE mis-splicing in both the *Mbnl* cDKO and DM1 cortex (Fig. 2e). MiEs constituted 4% (*n* = 1,185) of all detected SE events in wild-type (WT) mouse frontal cortex but represented 10% (*n* = 147) of mis-spliced SE events in the *Mbnl* cDKO, as well as 15% (*n* = 28) within mis-spliced ASD-risk genes from the SFARI, 23% (*n* = 6) from MSSNG-2022 and 33% (*n* = 9) from MSSNG-2017 studies. Similarly, miEs constituted 2% (*n* = 3,142) of all detected SE events in human control prefrontal cortex, 8% (101) of mis-spliced SE events in DM1, 19% (*n* = 28) in ASD-risk genes from SFARI, 17% (*n* = 3) from MSSNG-2022 and 47% (*n* = 7) from MSSNG-2017 studies. In total, we identified mis-spliced miE events in 33 genes that are present in both human DM1 and mouse *Mbnl* cDKO, including evolutionarily conserved miEs in high-confidence ASD-risk genes, such as *ANK2*, *TANC2* and *DMD* (Fig. 2f,g).

As previous studies have shown that miEs can locally modulate protein structure^26^, we performed comparative in silico modeling of peptides with and without miE-encoded amino acid (aa) sequences to test their potential for protein modulation. This analysis suggested some mis-spliced miEs modulate internal (such as ANK2 and NRXN1) or C-terminal (such as DMD and SHANK3) protein structures (Fig. 2h and Extended Data Fig. 2c,d). For example, inclusion of the highly conserved *Ank2* miE (12 nt) along with the use of a proximal alternative 3′ splice site (A3SS) results in a protein isoform with a Thr-Ile-Pro (mouse) or Thr-Met-Pro (human) aa sequence, whereas miE exclusion promotes distal A3SS usage (15 nt), and results in a protein isoform with a Leu-Arg-Ser-Phe (LRSF) aa sequence in both mouse and human containing a S901 phosphorylation site^53^ (Fig. 2h and Extended Data Fig. 2c). For Dmd, a 32-nt miE modulates the structure of the highly conserved dystrophin C terminus that interacts with other proteins and this miE exclusion has been shown to compromise muscle fiber maintenance in myotonic dystrophy (Extended Data Fig. 2d)^54^. Cumulatively, these data demonstrate that MBNLs regulate miE splicing in ASD-risk genes.

### ASD-risk gene splicing regulation in cortical development

**Fig. 3:**
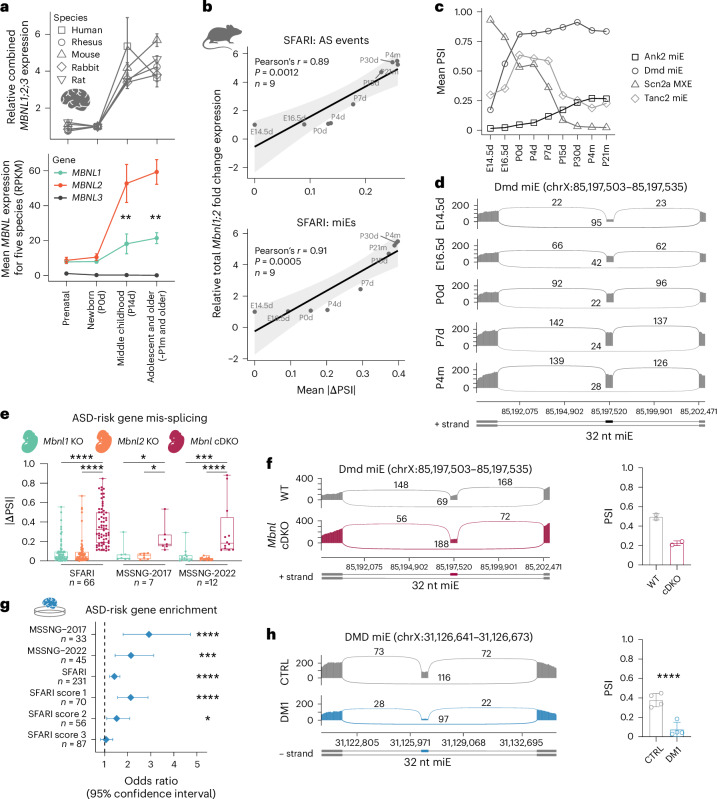
MBNL proteins govern developmental splicing transitions in ASD-risk genes. **a,** *MBNL1*, *MBNL2* and *MBNL3* gene expression levels in developing brains of five species: human (*n* = 49), rhesus (*n* = 17), mouse (*n* = 51), rabbit (*n* = 57) and rat (*n* = 57). Total *MBNL* expression relative to newborn/postnatal day 0 (P0d) (top). Mean *MBNL* expression for five species at each developmental stage (bottom). Significant differences between MBNL1 and MBNL2 expression at different developmental stages were determined by a two-tailed *t*-test; **P < 0.01. **b**, Strong positive correlation between *Mbnl* gene expression in developing WT mouse cortex and mean |ΔPSI | for MBNL-sensitive AS events (top) and miEs only (bottom) in SFARI genes. Black diagonal lines are the linear regression lines, and light gray areas are the 95% confidence intervals. *P* value for a two-tailed test. **c**, Splicing change in the developing mouse cortex for four MBNL-sensitive AS events in high-confidence ASD-risk genes. Embryonic days 14.5–16.5, postnatal days 0–30, and postnatal months 4–21 (*n* = 2 for each time point). **d**, Dmd miE splicing transitions during mouse cortical development. **e**, ASD-risk gene mis-splicing in *Mbnl1* KO (*n* = 2), *Mbnl2* KO (*n* = 2) and *Mbnl* cDKO (*n* = 2) mouse E18.5 d cortical neurons. Box plot shows the lower, middle and upper quartiles. Whiskers show minimum and maximum. Kruskal–Wallis test followed by Dunn’s multiple comparison test (*n*, mis-spliced AS events); *P_adj_ < 0.05, ***P_adj_ < 0.001, ****P_adj_ < 0.0001. **f**, Dmd miE mis-splicing in embryonic *Mbnl* cDKO (*n* = 2) versus WT (*n* = 2) RNA-seq. **g**, ASD-risk gene set enrichment analysis for mis-spliced genes in DM1 brain organoid. Diamonds represent the OR. Error bars depict the 95% confidence interval. **h**, DMD miE mis-splicing in in DM1 (*n* = 4) versus CTRL (*n* = 4) brain organoid RNA-seq. Data are presented as mean ± s.d. (**a**,**c**,**f**,**h**). *FDR = 0.018, ***FDR = 0.00026, ****FDR < 0.0001 (**g**,**h**). Source data

**Extended Data Fig. 3:**
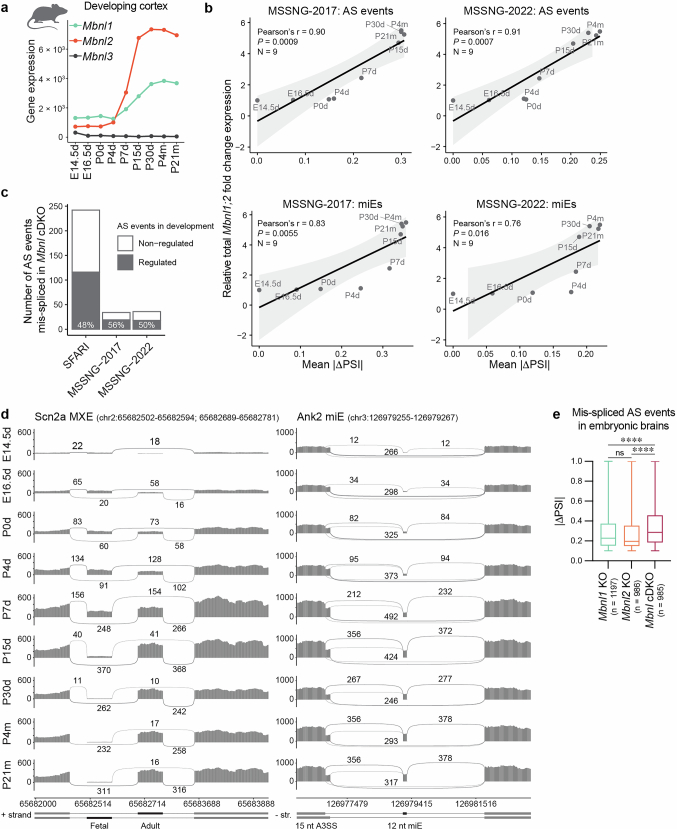
MBNL-mediated splicing transitions in ASD-risk genes during brain development. **a**, *Mbnl1*, *Mbnl2* and *Mbnl3* gene expression levels during WT mouse cortex development. Dots represent the mean values (N = 2). **b**, Strong positive correlation between *Mbnl* gene expression in developing WT mouse cortex and mean |ΔPSI | for MBNL-sensitive AS events (top) and miEs only (bottom) in MSSNG-2017 and MSSNG-2022 genes. The black diagonal lines represent the linear regression lines, and the light gray areas indicate the 95% confidence intervals. P-value for a two-tailed test. **c**, Proportion of MBNL-sensitive AS events differentially spliced (gray) in the developing cortex (E14.5 d, E16.5 d vs P4m, P21m). **d**, Scn2a MXE and Ank2 miE splicing transitions during WT cortical development. **e**, RNA mis-splicing in *Mbnl1* KO (N = 2), *Mbnl2* KO (N = 2) and *Mbnl* cDKO (N = 2) mouse E18.5 cortical neuron RNA-seq samples. Box plot shows the lower, middle, and upper quartiles. Whiskers show minimum and maximum. Kruskal–Wallis test followed by Dunn’s multiple comparison test (n = mis-spliced AS events); ^ns^*P*_*adj*_ = 0.11, ****P_*adj*_ < 0.0001. Source data

To assess the developmental splicing pattern of ASD-risk genes, we analyzed gene expression data for five mammalian brains at different developmental stages^55^. Our analysis showed an evolutionarily conserved increase of *MBNL* expression during neonate to middle childhood brain development (Fig. 3a). Although *MBNL1* and *MBNL2* are expressed at a similar level at early developmental stages and their expression increases simultaneously during brain development, *MBNL2* expression rises more profoundly and is approximately three-times higher than *MBNL1* in middle childhood and older brains (Fig. 3a and Extended Data Fig. 3a). To assess the association between *Mbnl1* and *Mbnl2* gene expression and MBNL-sensitive splicing transitions in the developing mouse cortex, we evaluated RNA-seq data from WT mice at nine developmental time points^56^. As anticipated, there is a strong correlation between *Mbnl* expression levels and mis-splicing level in ASD-risk genes from the SFARI and both MSSNG studies, and this is similar for MBNL-sensitive miEs (Fig. 3b and Extended Data Fig. 3b). Differential AS analysis demonstrated that 48–56% of mis-spliced AS events in ASD-risk genes were significantly changed between embryonic and adult cortex (Extended Data Fig. 3c). For example, *Scn2a1* mutually exclusive exons (MXE), *Ank2* miE, and *Dmd* miE splicing transitions occurred at early developmental stages to reach a plateau between 2 and 4 weeks of age (Fig. 3c,d and Extended Data Fig. 3d), which is consistent with the developmental expression patterns of *Mbnl* paralogs (Fig. 3a).

To assess whether prenatal MBNL loss influences splicing of ASD-risk genes, we analyzed RNA-seq data of primary embryonic cortical neuron samples from *Mbnl* cDKO, constitutive *Mbnl1*^−/−^ KO (hereafter *Mbnl1* KO), constitutive *Mbnl2*^−/−^ KO (hereafter *Mbnl2* KO) and WT mice^57^. Differential splicing analysis did not reveal profound mis-splicing of ASD-risk genes in single embryonic *Mbnl1* KO and *Mbnl2* KO (Fig. 3e and Extended Data Fig. 3e), as both *Mbnl1* and *Mbnl2* are expressed at comparably low levels in embryonic brains (Fig. 3a and Extended Data Fig. 3a) and they compensate for the loss of each other by regulating the same AS events^14^. As anticipated, we detected significant ASD-risk gene mis-splicing in embryonic *Mbnl* cDKO, including *Dmd* miE (Fig. 3f). This is further supported by significant enrichment of mis-spliced events in ASD-risk genes from the SFARI, MSSNG-2017 and MSSNG-2022 studies in the DM1 cortical organoids^51^, including the DMD miE (Fig. 3g,h). Overall, these results indicated that MBNL proteins govern the splicing patterns of multiple ASD-risk genes, including miEs, in the developing brain.

### *Mbnl2* KO causes ASD-risk gene mis-splicing in hippocampus

**Fig. 4:**
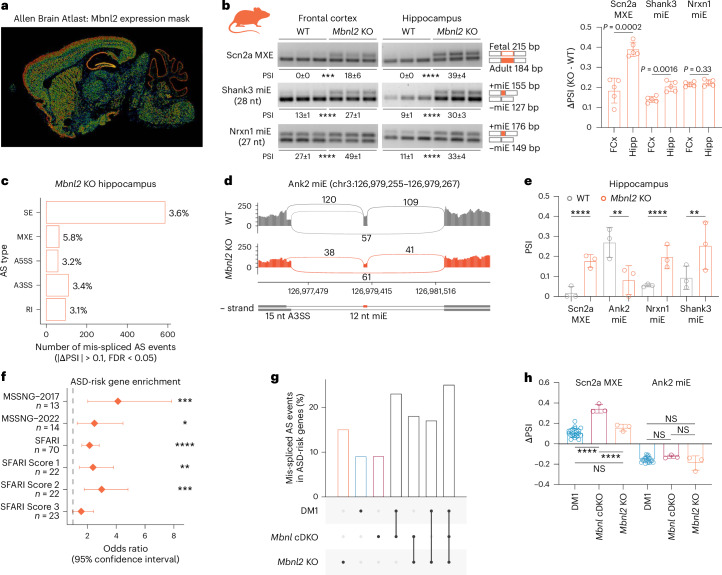
Mis-splicing in the *Mbnl2* KO hippocampus. **a**, The Allen Mouse Brain Atlas shows the normalized color-coded *Mbnl2* expression level (from blue, low to red, high) derived from the informatics data processing of in situ hybridization results (mouse.brain-map.org/gene/show/69724)^79^. **b**, Scn2a MXE, Nrxn1 miE and Shank3 miE mis-splicing in *Mbnl2* KO (*n* = 5) and littermate WT (*n* = 5) frontal cortex (FCx) and hippocampus (Hipp). Two-tailed *t*-test: ***P = 0.0001, ****P < 0.0001. **c**, The number and percentage of AS event types significantly mis-spliced in *Mbnl2* KO hippocampus (*n* = 3 females). **d**, Ank2 miE mis-splicing in *Mbnl2* KO RNA-seq. **e**, ASD-risk gene mis-splicing in *Mbnl2* KO (*n* = 3) versus WT (*n* = 3) hippocampus RNA-seq. **f**, ASD-risk gene set enrichment analysis for mis-spliced genes in *Mbnl2* KO. Diamonds represent OR. Error bars depict 95% confidence intervals. **g**, Percent of overlapping events annotated within ASD-risk genes among DM1 prefrontal cortex, *Mbnl* cDKO frontal cortex and *Mbnl2* KO hippocampus. **h**, Examples of overlapping mis-splicing events in DM1 (*n* = 21), *Mbnl* cDKO (*n* = 3) and *Mbnl2* KO (*n* = 3). One-way ANOVA followed by Tukey’s multiple comparisons test. NS, *P*_adj_ > 0.05, ****P_adj_ < 0.0001. Data are presented as means ± s.d. (**b**,**e**,**h**). *FDR = 0.011, **FDR < 0.01, ***FDR < 0.001, ****FDR < 0.0001 (**e**,**f**). Source data

**Extended Data Fig. 4:**
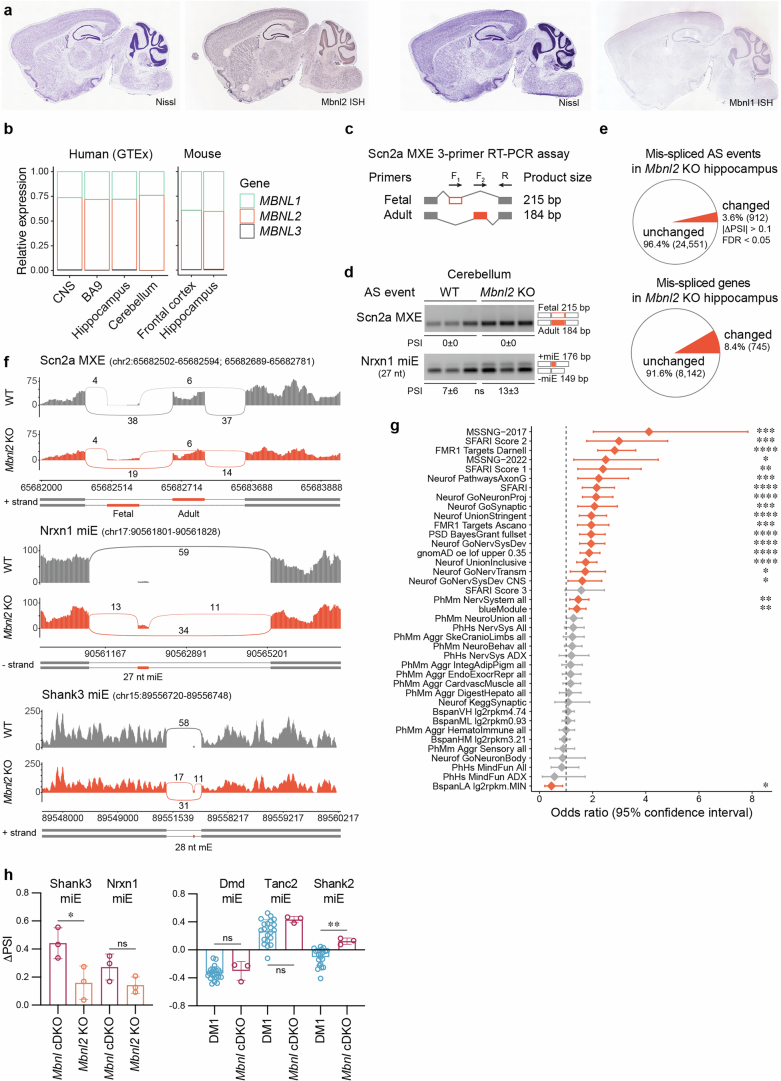
Mis-splicing in the mouse *Mbnl2* KO brain. **a**, Nissl bodies from the Allen Mouse Brain Atlas at the same slice position as Mbnl1 and Mbnl2 RNA *in situ* hybridization (ISH) results. Staining Nissl bodies serve as a reference for the ISH data. For ISH, specific digoxigenin tagged RNA probes were used to label cells expressing Mbnl1 (mouse.brain-map.org/gene/show/36037) and Mbnl2 (mouse.brain-map.org/gene/show/69724) transcripts^79^. **b**, Relative *MBNL1*, *MBNL2*, and *MBNL3* expression in multiple human and mouse brain regions. CNS - central nervous system. **c**, Schematic of the 3-primer Scn2a RT–PCR assay. **d**, Scn2a MXE and Nrxn1 miE mis-splicing in *Mbnl2* KO (N = 5) and littermate WT (N = 3) cerebellum. Two-tailed t-test; ^ns^*P* > 0.05. This result was repeated twice. **e**, Differential AS analysis in frontal hippocampus of *Mbnl2* KO compared to littermate WT control (N = 3 females). The percentage of mis-spliced AS events (left) and genes (right) in the *Mbnl2* KO. **f**, Examples of mis-splicing in ASD-risk genes in *Mbnl2* KO RNA-seq. **g**, ASD-relevant gene set enrichment analysis for mis-spliced genes in *Mbnl2* KO (N = 3). Diamonds represent the odds ratio. Error bars depict the 95% confidence interval. *FDR < 0.05, **FDR < 0.01, ***FDR < 0.001, ****FDR < 0.0001. **h**, Examples of overlapping mis-splicing events in DM1 (N = 21), *Mbnl* cDKO (N = 3), and *Mbnl2* KO (N = 3). Data are presented as mean values ± SD. One-way ANOVA followed by Tukey’s multiple comparisons test; ^ns^*P*_*adj*_ > 0.05, *P_*adj*_ = 0.0393, **P_*adj*_ = 0.0058. Source data

*Mbnl2* is the predominant gene paralog expressed in the adult human and mouse cerebral cortex, hippocampus and cerebellum (Fig. 4a and Extended Data Fig. 4a,b), which are regions known to be involved in ASD^58,59^. To test whether *Mbnl2* loss perturbs splicing of ASD-risk genes in multiple brain regions, we performed RT–PCR splicing analysis of *Scn2a* MXE, *Nrxn1* miE and *Shank3* miE in frontal cortex, hippocampus and cerebellum of adult *Mbnl2* KO. Two of three tested AS events demonstrated that mis-splicing was most profound in the hippocampus (Fig. 4b and Extended Data Fig. 4c,d). Thus, we performed differential splicing analysis on RNA-seq data from *Mbnl2* KO hippocampus^60^. In total, 4% (*n* = 912) of AS events were perturbed in 8% (*n* = 745) of detected genes, including *Scn2a*, *Ank2*, *Nrxn1* and *Shank3* (Fig. 4c–e and Extended Data Fig. 4e,f). As observed for the *Mbnl* cDKO, ASD-risk gene lists were significantly enriched among the mis-spliced genes in the *Mbnl2* KO hippocampus (Fig. 4f and Extended Data Fig. 4g). Although the overall degree of mis-splicing varied between analyzed species, models, brain regions, and experiments (Extended Data Fig. 4h), we found that 17–25% of overlapping mis-spliced events are located in ASD-risk genes (Fig. 4g). The most consistently mis-spliced events were *Ank2* miE and *Scn2a* MXE (Fig. 4h). Therefore, *Mbnl2* loss alone impacts the AS of ASD-risk genes in multiple adult ASD-relevant brain regions, including the hippocampus.

### MBNLs directly regulate splicing of ASD-relevant microexons

**Fig. 5:**
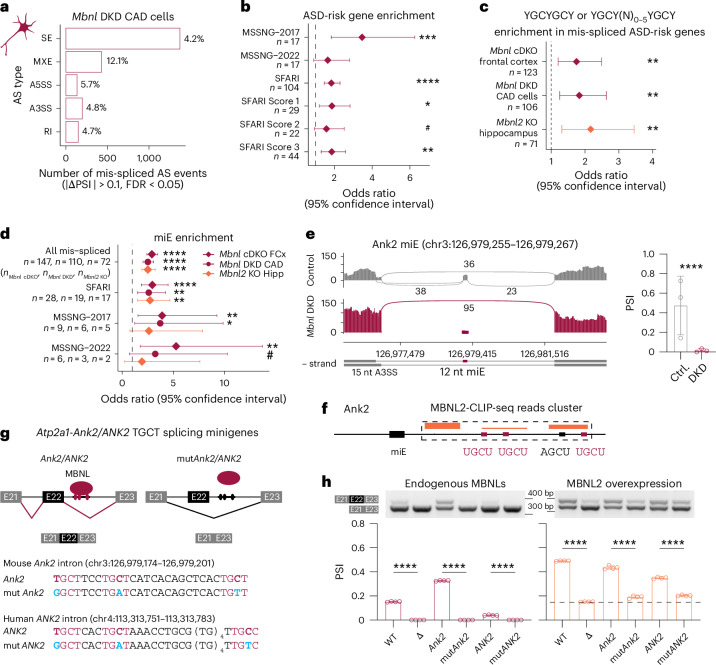
MBNL proteins directly regulate Ank2 miE splicing. **a**, The number and percentage of AS event types significantly mis-spliced in *Mbnl* DKD CAD cells (*n* = 3). **b**, ASD-risk gene set enrichment analysis for mis-spliced genes in *Mbnl* DKD cells. **c**, MBNL-binding motif enrichment in mis-spliced ASD-risk genes. **d**, miE enrichment analysis for mis-spliced ASD-risk gene sets. **e**, Ank2 miE mis-splicing in *Mbnl* DKD RNA-seq. **f**, MBNL2-CLIP-seq reads (orange boxes, combined *n* = 3 females) in the Ank2 miE downstream intron containing three conserved (magenta) and one suboptimal (black) motifs. **g**, Schematic of limited heterologous *Atp2a1* splicing minigenes and regulation by MBNL proteins. MBNL-binding sequences (magenta) identified in mouse *Ank2* and human *ANK2* and their mutants (blue). **h**, Heterologous *Atp2a1* splicing minigene regulation by MBNL proteins in HeLa cells (*n* = 4). Unpaired two-tailed *t*-test: ****P < 0.0001. Data are presented as mean ± s.d. (**e**,**h**). Diamonds represent OR (**b**–**d**). Error bars depict 95% confidence intervals. ^#^FDR ≤ 0.10, ^*^FDR < 0.05, **FDR < 0.01, ***FDR < 0.001, ****FDR < 0.0001. Source data

**Extended Data Fig. 5:**
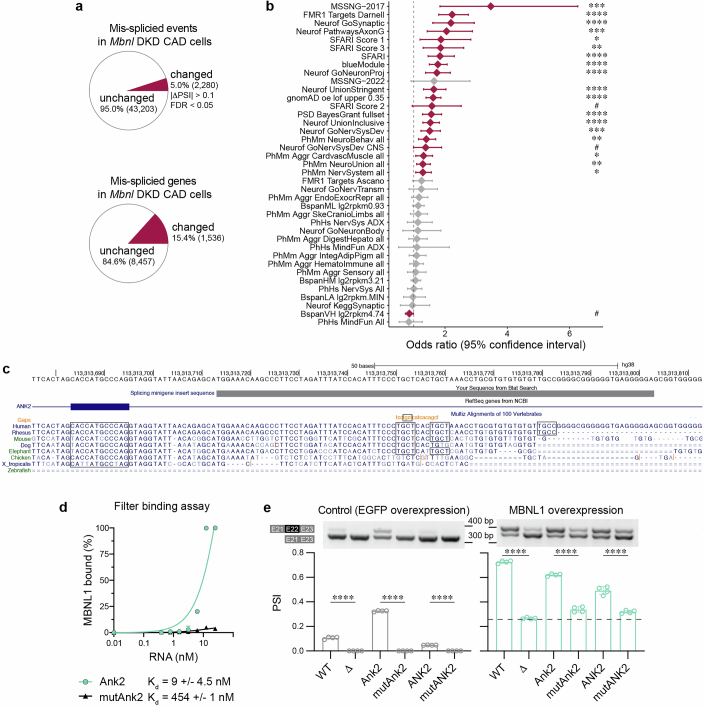
Mis-splicing in the *Mbnl* DKD CAD cells. **a**, Differential AS analysis in *Mbnl* DKD compared to control (N = 3) CAD cells. The percentage of mis-spliced AS events (left) and genes (right) in the *Mbnl* DKD. **b**, ASD-relevant gene set enrichment analysis for mis-spliced genes in *Mbnl* DKD (N = 3). Diamonds represent the odds ratio. Error bars depict the 95% confidence interval. ^#^FDR ≤ 0.10, *FDR < 0.05, **FDR < 0.01, ***FDR < 0.001, ****FDR < 0.0001. **c**, UCSC Genome browser view of human *ANK2* illustrates the conservation of miE 12 bp and intronic MBNL-binding motifs across multiple species. **d**, Binding affinity of recombinant MBNL1 to normal and mutant Ank2 RNA sequences expressed as a K_d_ value (3 replicas). **e**, Heterologous *Atp2a1* splicing minigene regulation by MBNL proteins in HeLa cells (N = 4). Two-tailed t-test; ****P < 0.0001. **d,e**, Data are presented as mean values ± SD. Source data

To support the observation that MBNLs directly regulate splicing of high-confidence ASD-risk genes, we performed differential AS analysis on RNA-seq data from a mouse brain-derived catecholaminergic (CAD) neuronal cell line with siRNA-mediated *Mbnl1*;*Mbnl2* double KD (hereafter *Mbnl* DKD)^57^. In total, 5% (*n* = 2,280) of AS events in 15% (*n* = 1,536) of detected genes were mis-spliced (Fig. 5a and Extended Data Fig. 5a) and previously analyzed ASD-risk gene sets were significantly enriched for mis-splicing (Fig. 5b and Extended Data Fig. 5b). As expected, a genome-wide distribution analysis of high-affinity MBNL-binding sequences identified a significant enrichment of these sequences in mis-spliced ASD-risk genes in all MBNL depletion models (Fig. 5c). We also observed an enrichment of mis-spliced miEs in ASD-risk genes similar to other analyzed mouse model brains, including Ank2 miE (Fig. 5d,e).

As our data indicated MBNL proteins preferentially regulate miE splicing in ASD-risk genes, we selected the highly conserved Ank2 miE, which was consistently mis-spliced in various models, to study the mechanism underlying miE splicing. To assess whether MBNL directly regulates Ank2 miE inclusion, we analyzed MBNL2 crosslinking and immunoprecipitation sequencing (CLIP-seq) samples from hippocampi of WT adult mice^60^. Previous studies demonstrate that MBNL-binding to an intron that is downstream of an alternative exon promotes its inclusion^60,61^; similarly, we identified an MBNL2-CLIP-seq cluster covering a conserved TGCT(N)_3_TGCT(N)_13-18_TGCT/C sequence ~55 nt downstream of the 5′ splice site in *Ank2*/*ANK2* intron (Fig. 5f and Extended Data Fig. 5c). Using a filter-binding assay, we demonstrated that recombinant MBNL1 protein binds to this RNA sequence with high affinity (*K*_d_ = 9 ± 4.5 nM) but does not bind to the RNA with mutated motifs (*K*_d_ = 454 ± 1 nM) (Extended Data Fig. 5d). To test whether MBNL can promote alternative exon inclusion by binding to this sequence, we used our previously developed splicing minigene containing MBNL-regulated *Atp2a1* exon 22 (E22)^44^. We substituted the MBNL-binding site in *Atp2a1* intron 22 with the normal and mutant mouse *Ank2* and human *ANK2* conserved intronic sequences (Fig. 5g,h). We co-transfected HeLa cells with these *Atp2a1* minigenes and measured E22 inclusion level by PCR with reverse transcription (RT–PCR). Unlike mutant *Ank2/ANK2* and negative control (Δ) minigenes, normal *Ank2/ANK2* and positive control (WT) minigenes were sensitive to endogenous MBNLs (Fig. 5h). Further, upon transfection of *Atp2a1* minigenes and MBNL expression vectors, E22 was included at significantly higher levels in normal *Ank2/ANK2* than in mutants (Fig. 5h and Extended Data Fig. 5e). Cumulatively, these data confirm that MBNLs directly regulate Ank2 miE splicing in an ASD-risk gene.

### Mis-splicing of ANK2 microexon in ASD and DM1

**Fig. 6:**
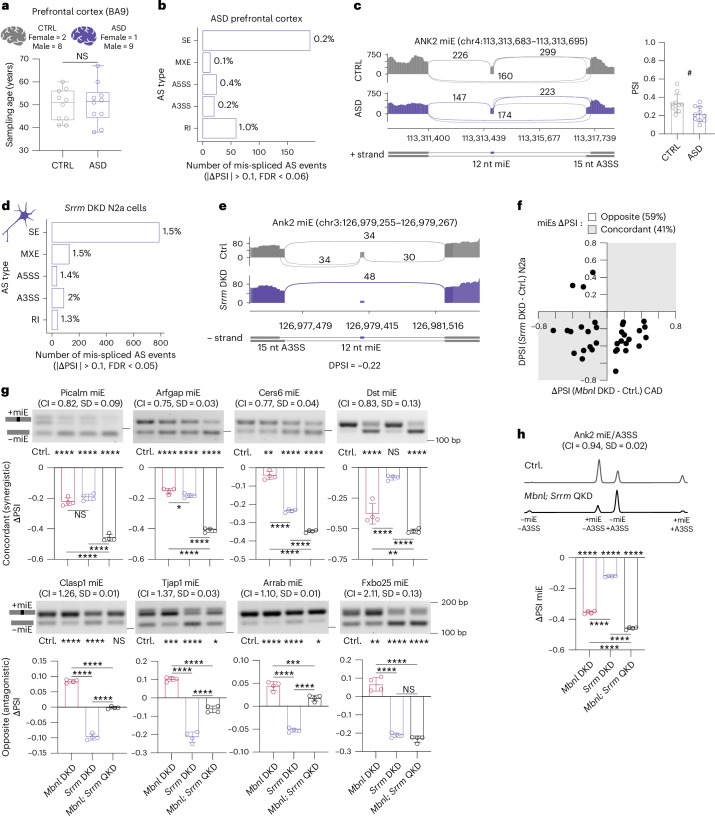
MBNL and SRRM regulate the same miEs. **a**, Age and sex distribution of ASD and CTRL prefrontal cortex samples in PsychENCODE RNA-seq data. ASD was confirmed by the Autism Diagnostic Interview-Revised (*n* = 8) or supported by records (*n* = 2). Box plot shows the lower, middle and upper quartiles. Whiskers show minimum and maximum. Two-tailed *t*-test; NS, *P* = 0.83. **b**, The number and percentage of AS event types significantly mis-spliced in ASD. **c**, ANK2 miE mis-splicing in ASD. Sashimi plots of ASD (*n* = 8) and CTRL (*n* = 8) RNA-seq. ^#^FDR = 0.056. **d**, The number and percentage of AS event types significantly mis-spliced in *Srrm* DKD N2a cells (*n* = 2). **e**, Ank2 miE mis-splicing in *Srrm* DKD RNA-seq (*n* = 2). **f**, Overlapping mis-spliced miEs in *Srrm* DKD CAD and *Mbnl* DKD N2a cells. **g**, Selected miE splicing analysis in *Mbnl* DKD, *Srrm* DKD and *Mbnl;Srrm* QKD N2a cells (*n* = 4). **h**, Capillary electrophoresis analysis of Ank2 miE and A3SS. CI, combination index (**g**,**h**). One-way ANOVA followed by Tukey’s multiple comparisons test; NS, *P*_adj_ > 0.05, *P_adj_ < 0.05, **P_adj_ < 0.01, ***P_adj_ < 0.001, ****P_adj_ < 0.0001. Data are presented as mean ± s.d (**c**,**g**–**h**). Source data

**Extended Data Fig. 6:**
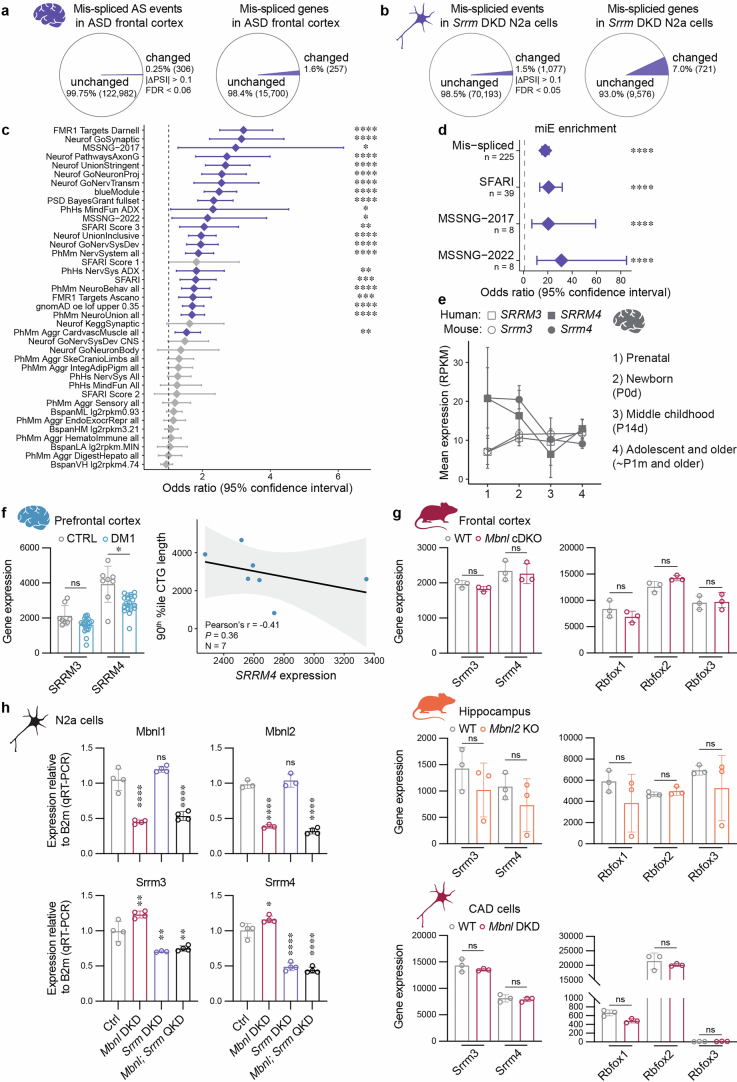
Mis-splicing in the *Srrm* DKD N2a cells. **a**, Differential AS analysis in ASD (N = 10) compared to CTRL (N = 10) prefrontal cortex. The percentage of mis-spliced AS events (left) and genes (right) in the ASD. **b**, Differential AS analysis in *Srrm* DKD compared to control (N = 2) N2a cells. The percentage of mis-spliced AS events (left) and genes (right) in the *Srrm* DKD. **c**, ASD-relevant gene set enrichment analysis for mis-spliced genes in *Srrm* DKD (N = 2). **d**, miE enrichment analysis for mis-spliced ASD-risk gene sets. **c,d**, Diamonds represent the odds ratio. Error bars depict the 95% confidence interval. *FDR < 0.05, **FDR < 0.01, ***FDR < 0.001, ****FDR < 0.0001. **e**, *Srrm3/SRRM3* and *Srrm4/SRRM4* gene expression levels in developing human (N = 49) and mouse (N = 51) brains. **f**, *SRRM3* and *SRRM4* gene expression levels in DM1 prefrontal cortex RNA-seq shows lack of correlation between *DMPK*-CTG lengths and *SRRM4* gene expression. The black diagonal line represents the linear regression line, and the light gray area indicates the 95% confidence interval. P-value for a two-tailed test. **g**, *Srrm3*, *Srrm4*, *Rbfox1*, *Rbfox2*, and *Rbfox3* gene expression levels in *Mbnl* cDKO frontal cortex (N = 3), *Mbnl2* KO hippocampus (N = 3), and *Mbnl* DKD CAD cells (N = 3) RNA-seq. **f,g**, DESeq2; ^ns^*P*_adj_ > 0.05, *P_adj_ = 0.01. **h**, *Mbnl1* and *Mbnl2* gene expression levels in control (Ctrl; N = 4), *Mbnl* DKD (N = 4), *Srrm* DKD (N = 4), and *Mbnl;Srrm* QKD (N = 4) N2a cells measured by RT-qPCR. One-way ANOVA followed by Dunnett’s multiple comparisons test; ^ns^*P*_adj_ > 0.05, *P_adj_ < 0.05, **P_adj_ < 0.01, ****P_adj_ < 0.0001. **e-h**, Data are presented as mean values ± SD. Source data

To ascertain whether there were common mis-spliced genes and AS events between DM1 and ASD, we retrieved human adult idiopathic ASD prefrontal cortex (BA9) and matched control samples from the PsychENCODE Consortium (Fig. 6a)^24,62^. Our differential AS analysis revealed 0.3% (*n* = 306) mis-spliced events in 2% (*n* = 257) of analyzed genes (Fig. 6b and Extended Data Fig. 6a) with 15 mis-spliced AS events overlapping between DM1 and ASD (OR = 3.5, *P* = 5.7 × 10^−5^, Fisher’s exact test), including ANK2 miE (Fig. 6c).

**Extended Data Fig. 7:**
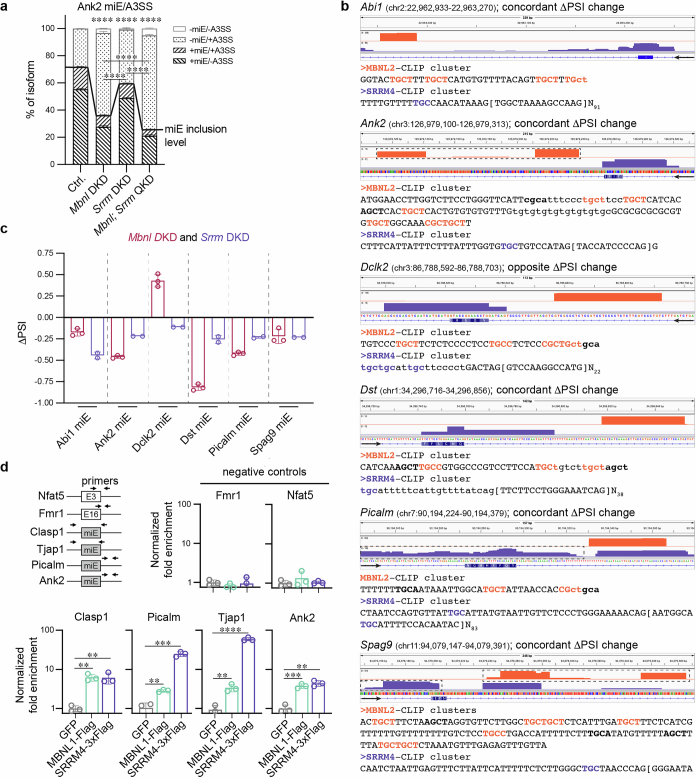
MBNL and SRRM proteins bind to primary transcripts containing miE. **a**, Capillary electrophoresis analysis of endogenous Ank2 miE and A3SS in control (Ctrl; N = 4), *Mbnl* DKD (N = 4), *Srrm* DKD (N = 4), and *Mbnl;Srrm* QKD (N = 4) N2a cells. One-way ANOVA followed by Tukey’s multiple comparisons test; ****P_*adj*_ < 0.0001. **b**, Genome browser view of MBNL2-CLIP-seq clusters (orange) from the hippocampus and SRRM4-CLIP-seq clusters from N2a cells in vicinity of six mis-spliced miEs. The cluster sequences are provided, with conserved (orange or purple) and suboptimal (black) protein binding motifs bolded. Lowercase letters indicate sequences not covered by the CLIP-seq reads, and square brackets indicate miE boundaries. **c**, Mis-splicing of six miEs in *Mbnl* DKD CAD (magenta; N = 3) and *Srrm* DKD N2a (purple; N = 2) cells. *FDR < 0.05, **FDR = < 0.01, ****FDR < 0.0001. **d**, Clasp1, Picalm, Tjap1, Ank2 enrichment in GFP (N = 3), MBNL1-Flag (N = 3) and SRRM4-3xFlag (N = 3) RNA co-IP. Top right, randomly selected Nfat5 and Fmr1 pre-mRNAs serve as negative controls. Top left, a scheme of qRT–PCR-analyzed pre-mRNA fragments in RNA co-IP; black arrows depict regions bound by forward and reverse primers. Multiple two-sample t-test followed by Holm-Šídák correction for multiple comparisons; **P_*adj*_ < 0.01, ***P_*adj*_ < 0.001, ****P_*adj*_ < 0.0001. **a,c,d**, Data are presented as mean values ± SD. Source data

Previous reports have linked neuronal miE mis-splicing in idiopathic ASD brains to reduced SRRM4 expression^26^. Like MBNL1 and MBNL2, SRRM3 and SRRM4 are paralogs that regulate the same set of neuronal miEs^63^. To ascertain whether there are common mis-spliced ASD-relevant miEs upon MBNL loss and SRRM loss, we retrieved RNA-seq data from mouse Neuro2a (N2a) cells with siRNA-mediated *Srrm3*; *Srrm4* DKD (hereafter *Srrm* DKD)^63^. Our differential AS analysis revealed 2% (*n* = 1,077) mis-spliced events in 7% (*n* = 721) of analyzed genes (Fig. 6d and Extended Data Fig. 6b), and ASD-risk gene lists were significantly enriched (Extended Data Fig. 6c). In agreement with previous studies, *Srrm* DKD preferentially altered miE splicing, including those in ASD-risk genes (Extended Data Fig. 6d). Unexpectedly, we identified a nonrandom overlap of 153 mis-spliced AS events between *Mbnl* DKD CAD and *Srrm* DKD N2a (OR = 4.5, *P* = 4.0 × 10^−44^, Fisher’s exact test), including Ank2 and 32 other altered miE events (OR = 2.0, *P* = 4.5 × 10^−3^, Fisher’s exact test) (Fig. 6e). Notably, 41% of overlapping mis-spliced miEs showed concordant, and 59% showed opposite, ΔPSI changes in *Mbnl* DKD and *Srrm* DKD (Fig. 6f). To support this analysis, we chose Ank2 miE and eight randomly selected mis-spliced miEs, four from each of the concordant and opposite groups. We tested their splicing profiles in *Mbnl* DKD, *Srrm* DKD, combined *Mbnl* DKD and *Srrm* DKD (hereafter *Mbnl*;*Srrm* QKD) in N2a cells (Extended Data Fig. 6h). Our RT–PCR analysis confirmed that splicing of all selected miEs was regulated by both MBNL and SRRM proteins (Fig. 6g). Of note, all the concordant miE events identified in both RNA-seq and RT–PCR analyses demonstrated exon exclusion (ΔPSI < 0), including Ank2 miE (Fig. 6h). The combination index (CI) was calculated to further evaluate the combinatory effect of MBNL and SRRM proteins for miEs. All five concordant miEs were regulated synergistically (CI < 1) by MBNL and SRRM proteins, including Ank2 miE (Fig. 6g,h and Extended Data Fig. 7a). On the other hand, the four miEs with opposite ΔPSI changes were regulated antagonistically by those proteins (CI > 1) (Fig. 6g).

**Fig. 7:**
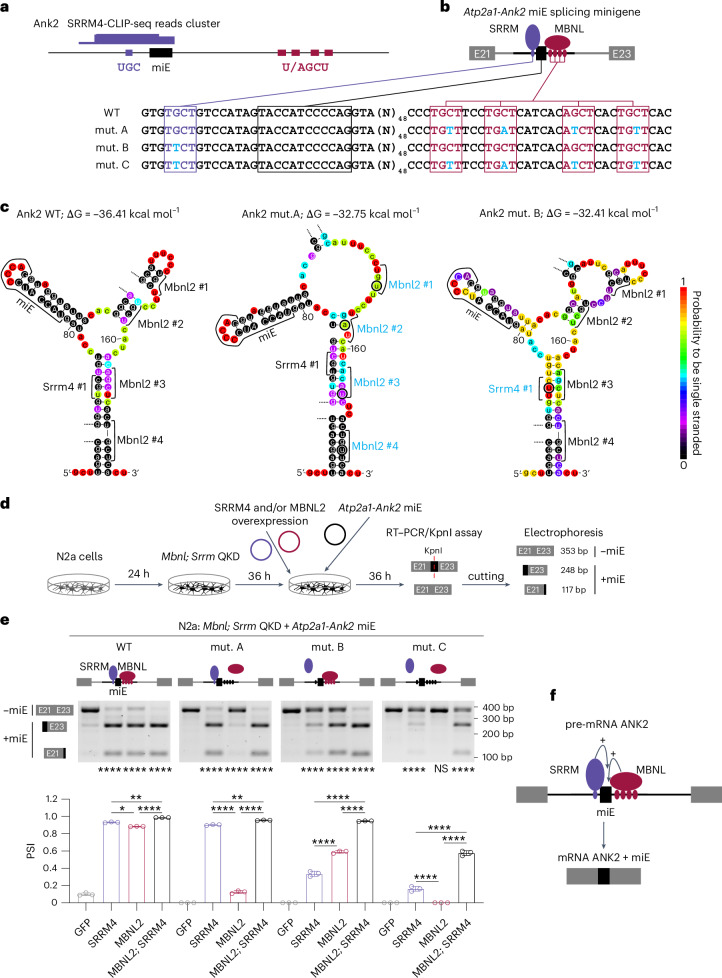
Ank2 miE regulation by MBNL and SRRM proteins. **a**, SRRM4-CLIP-seq reads coverage (purple box) in the Ank2 miE upstream intron containing the UGC motif. **b**, Schematic of heterologous *Atp2a1-Ank2* miE splicing minigenes and regulation by MBNL and SRRM proteins. MBNL (magenta) and SRRM (purple) binding sequences identified in mouse *Ank2* and their mutants (blue). **c**, Proposed secondary structures for normal and mutant Ank2 RNAs. The optimal thermodynamic stability of the structure is expressed in Gibbs free energy (∆G) in kcal mol^−1^ for the reaction at 37 °C using mfold software. **d**, Experimental design. **e**, Atp2a1-Ank2 miE splicing analysis in N2a experiment (*n* = 3). Data are presented as mean values ± s.d. One-way ANOVA followed by Tukey’s multiple comparisons test; NS, *P*_adj_ > 0.05, *P_adj_ < 0.05, **P_adj_ < 0.01, ****P_adj_ < 0.0001. **f**, SRRM and MBNL proteins directly bind to Ank2 and synergistically promote miE inclusion. Source data

To test whether both MBNL and SRRM can bind to the pre-mRNAs of altered miE transcripts, we reanalyzed MBNL2-CLIP-seq along with SRRM4-CLIP-seq data from an N2a cell line. SRRM4 promotes neuronal miE inclusion by binding to an intronic UGC motif 2–20 nt upstream of the regulated exon^63,64^, whereas MBNL proteins bind within ~300 nt of downstream to intronic sequence to induce the same change^61,65^. MBNL binding to exonic or within ~300 nt of upstream intronic sequence inhibits exon inclusion. We identified six high-confidence transcripts that meet these criteria, five of which showed concordant ΔPSI changes, while one showed opposite ΔPSI changes (Extended Data Fig. 7b,c). For example, our analysis revealed a SRRM4-CLIP-seq cluster of reads covering a conserved UGC motif 9 nt upstream of the Ank2 miE (Fig. 7a), and there were no reads supporting SRRM4 interaction with the MBNL-binding site (Extended Data Fig. 7b). To support these results, we overexpressed Flag-tagged MBNL1 and SRRM4 in N2a cells, followed by immunoprecipitation and qRT–PCR analysis of co-immunoprecipitated RNAs. We detected significant enrichment of Ank2 and three other randomly selected endogenous RNA targets, suggesting potential RBP–pre-mRNA interactions^66^ (Extended Data Fig. 7c).

**Extended Data Fig. 8:**
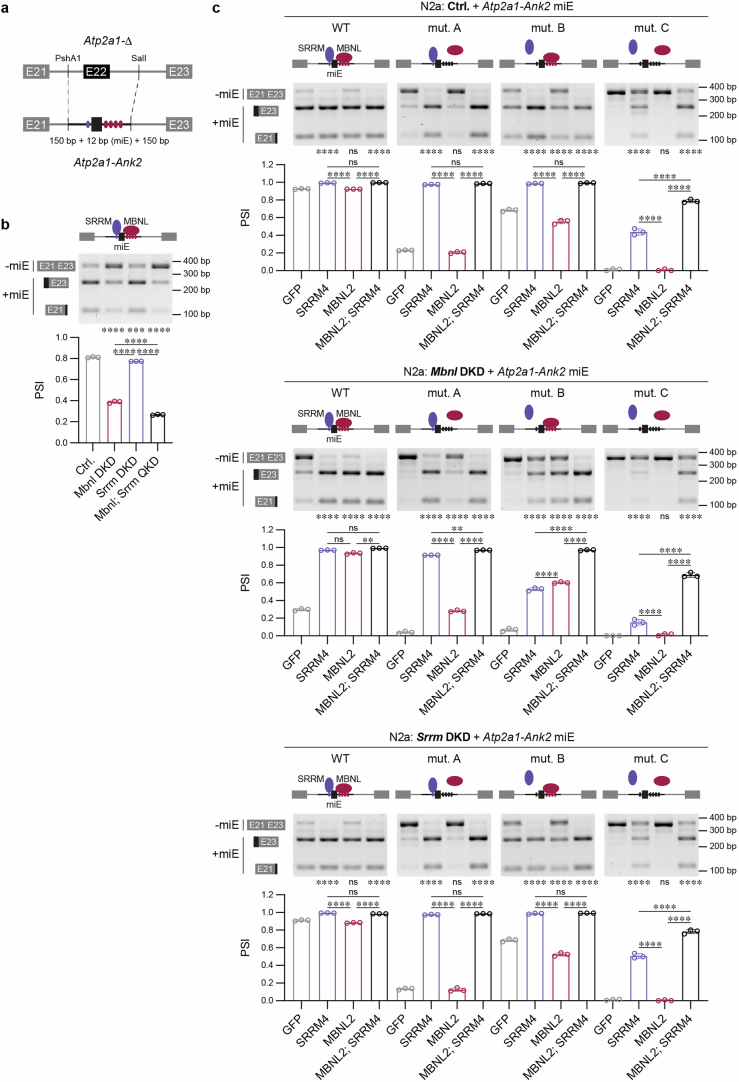
MBNL and SRRM proteins directly bind to Ank2 and regulate miE splicing. **a**, Schematic of heterologous *Atp2a1-Ank2* miE splicing minigenes production. MBNL (magenta) and SRRM (purple) binding sequences identified in mouse *Ank2*. **b,c**, *Atp2a1-Ank2* miE splicing analysis in N2a experiments (N = 3). One-way ANOVA followed by Tukey’s multiple comparisons test; ^ns^*P*_*adj*_ > 0.05, **P_*adj*_ < 0.01, ***P_*adj*_ < 0.001, ****P_*adj*_ < 0.0001. Data are presented as mean values ± SD. Source data

To further investigate the Ank2 miE regulatory mechanism by MBNL and SRRM4, we generated four heterologous *Ank2* constructs containing the miE and flanking intronic sequences with mutated SRRM and/or MBNL-binding sites (Fig. 7b and Extended Data Fig. 8a). In silico modeling predicted that the RNA secondary structure in the vicinity of Ank2 miE is not significantly affected by these mutations (Fig. 7c). To achieve the maximum dynamic range of miE splicing change, we performed knockdowns of *Srrm3;4* and/or *Mbnl1;2* in N2a cells, followed by MBNL2 and/or SRRM4 overexpression (Fig. 7d,e and Extended Data Fig. 8b,c). The exogenous Ank2 splicing change was analyzed by RT–PCR, followed by restriction enzyme cleavage of the miE containing isoform. Our results suggest that both proteins have a synergistic effect (CI = 0.82, s.d. = 0.01) on Ank2 miE inclusion by interacting with distinct sequences (Fig. 7e,f and Extended Data Fig. 8c).

In contrast to the increased postnatal expression of *MBNL1 and MBNL2* (Fig. 3a), *SRRM4* has higher expression in embryonic compared to postnatal brain in human and mouse (Extended Data Fig. 6e). To test the possibility that MBNL and SRRM can also modulate miE splicing indirectly by altering each other’s expression, we assessed Srrm and Mbnl steady-state levels in previously analyzed models. Similar to RBFOX1, we noticed a 28% reduction of SRRM4 RNA in human DM1 brain, which did not correlate with CTG^exp^ length (Extended Data Fig. 6f). Like RBFOX, Srrm RNA steady-state levels were unchanged in mouse and cell models of *Mbnl* KO (Extended Data Fig. 6g). Furthermore, *Srrm* DKD did not affect Mbnl steady-state RNA levels in N2a cells (Extended Data Fig. 6h). These results indicate that the MBNL and SRRM proteins regulate overlapping networks of splicing events, which have either synergistic or compensatory effects on inclusion of miEs.

### Behavioral phenotypes in *Mbnl2* KO and *Dmpk*-CTG^exp^ KI mice

**Fig. 8:**
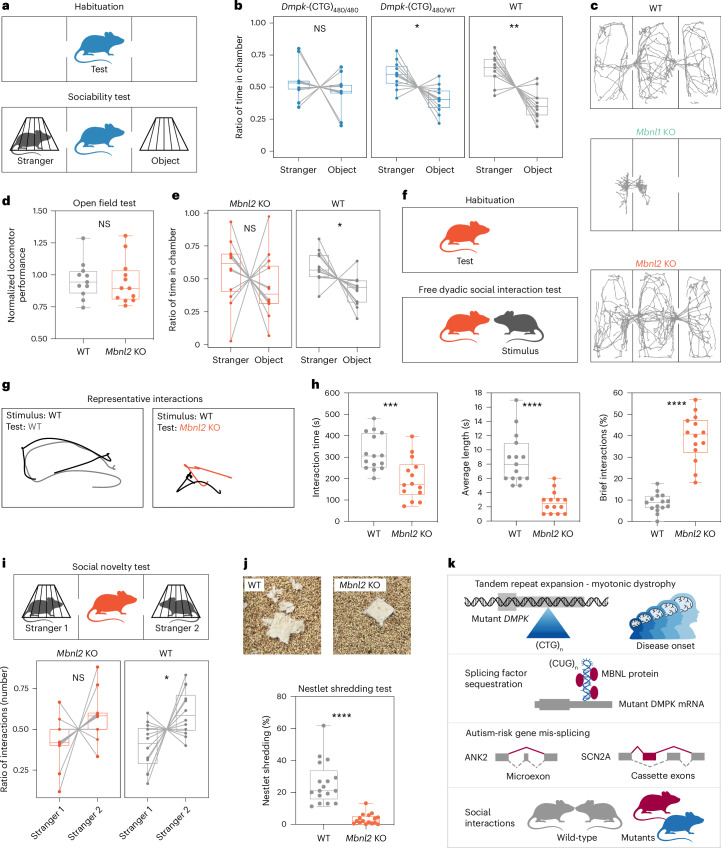
Social behavior deficits in two DM1 mouse models. **a**, Scheme of the three-chamber habituation and sociability test. **b**,**e**, Ratio of time the test mice spent in the chamber with a novel animal (stranger) and object during the sociability test. Time spent in the middle chamber is not included. **b**, WT mice (*n* = 11), *Dmpk*-(CTG)_480/WT_ (*n* = 11), and *Dmpk*-(CTG)_480/480_ (*n* = 11). Paired two-tailed *t*-test; NS, *P* = 0.38 (*t* = 0.9134, d.f. 10), *P = 0.013 (*t* = 2.997, d.f. 10), **P = 0.0025 (*t* = 4.012, d.f. 10). **c**, Representative mouse movement during the habituation phase. **d**, Normalized mean values for 13 parameters measured in the automated open field test. WT mice (*n* = 11), *Mbnl2* KO (*n* = 12). Two-tailed *t*-test; NS, *P* = 0.83 (*t* = 0.2131, d.f. 21). **e**, WT mice (*n* = 12), *Mbnl2* KO (*n* = 12); two-tailed *t*-test; NS, *P* = 0.43 (*t* = 0.8193, d.f. 11), *P = 0.023 (*t* = 2.641, d.f. 11). **f**, Scheme of the free dyadic social interaction test. **g**, Representative mouse interactions. **h**, Mouse interactions analysis. WT (*n* = 15), *Mbnl2* KO (*n* = 14). Interaction time and brief interaction; two-tailed *t*-test; ***P = 0.00098 (*t* = 3.696, d.f. 27), ****P < 0.0001 (*t* = 9.842, d.f. 27). Average length; two-tailed Mann–Whitney *U*-test; ****P < 0.0001. **i**, Scheme of the three-chamber social novelty test. Ratio of interactions with novel animal (stranger 2) and familiar animal (stranger 1) during the social novelty test. WT mice (*n* = 12), *Mbnl2* KO (*n* = 12). Paired two-tailed *t*-test: NS, *P* = 0.078 (*t* = 1.941, d.f. 11), *P = 0.025 (*t* = 2.594, d.f. 11). **j**, Representative photos of nestlet shredding. WT (*n* = 17), *Mbnl2* KO (*n* = 16); two-tailed Mann–Whitney *U*-test; ****P < 0.0001. Box plot shows the lower, middle and upper quartiles (**b**,**d**,**e**,**h**–**j**). Whiskers show minimum and maximum. **k**, Molecular mechanism of autism associated with myotonic dystrophy. Source data

Children with DM1 have a higher incidence of impaired social interaction and communication skills^67^; thus, we examined social behavior in our DM1 mouse models. We first selected heterozygous and homozygous *Dmpk*-(CTG)_480_ KI mouse models, as they reproduce characteristic DM1 pathological molecular signatures, including MBNL sequestration on *Dmpk*-(CUG)_480_ RNAs, RNA mis-splicing in vulnerable cell types and DMPK protein loss^68,69^. Notably, these molecular phenotypes are significantly exaggerated in the homozygous compared to heterozygous *Dmpk*-(CTG)_480_ KI mice^68,69^. WT littermate controls and heterozygous *Dmpk*-(CTG)_480/WT_ KI mice spent significantly more time in the chamber with a novel animal (stranger) than a novel object in the three-chamber test of sociability (Fig. 8a,b). In contrast, homozygous *Dmpk*-(CTG)_480/480_ KI mice showed no significant preference for the chamber with the novel animal over the novel object (Fig. 8b), signifying a lack of sociability.

**Extended Data Fig. 9:**
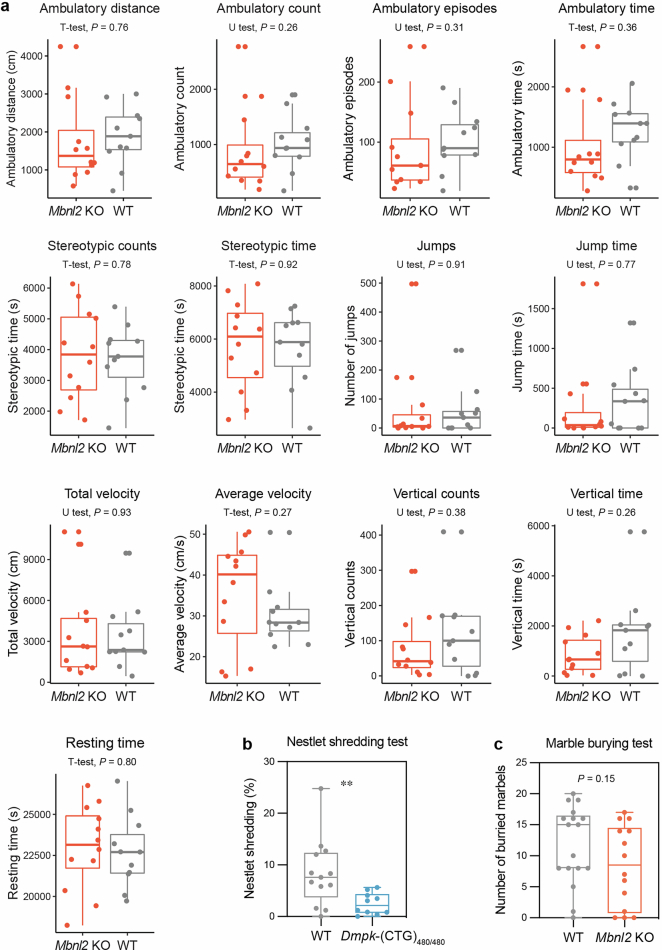
Lack of a locomotor deficit in *Mbnl2* KO mice. **a**, 13 parameters were measured during the 25-minute (5–30 min) interval of the open field test. WT mice (N = 11), *Mbnl2* KO (N = 12). Two-tailed t-test or two-tailed Mann-Whitney (U) test. **b**, Percentage of nestlet shredding. WT mice (N = 13), *Dmpk*-(CTG)_480/480_ (N = 10). Two-tailed Mann-Whitney test; **P = 0.0039. **c**, Number of buried marbles. WT mice (N = 17), *Mbnl2* KO (N = 14). Two-tailed t-test: *P* = 0.1455 (t = 1.496, df=29). **a,b,c**, Box plot shows the lower, middle, and upper quartiles. Whiskers show minimum and maximum. Source data

To test the hypothesis that MBNL loss underlies the social deficit, we evaluated *Mbnl1* and *Mbnl2* KO mouse models. The *Mbnl1* KO is characterized by muscle (such as myotonia), immune system and vision pathology^18,70^, resulting in limited paths of exploration (Fig. 8c) that preclude assessment of social interaction. In contrast, the *Mbnl2* KO exhibits central nervous system abnormalities, including neuronal morphology and synaptic changes^49,60,71^. *Mbnl2* KO mice did not exhibit significant exploratory deficits in the three-chamber test (Fig. 8c), which was further confirmed in the open field (Fig. 8d and Extended Data Fig. 9a). Like homozygous *Dmpk*-(CTG)_480/480_ KI, and in contrast to WT littermate controls, *Mbnl2* KO mice did not spend significantly more time in the chamber with a novel animal (Fig. 8e). To confirm and extend the social interaction deficits in *Mbnl2* KO, we also examined free dyadic social interaction in WT or *Mbnl2* KO mice on a different genetic background (Fig. 8f). We found that *Mbnl2* KO test mice spent significantly less time interacting with the novel stimulus mouse compared to WT littermate test mice, despite a significant increase in the total number of interactions (Fig. 8g,h). There was a significant reduction in the average length of interaction bouts between *Mbnl2* KO and stimulus mice, along with an increase in the incidence of brief interactions (less than 1 s; Fig. 8h). While WT mice frequently pursued stimulus mice to maintain an interaction, *Mbnl2* KO test mice were less likely to do so, and engaged in brief interactions before retreating (Fig. 8h). WT test mice were frequently dominant and engaged in mounting and pinning of stimulus mice, whereas *Mbnl2* KO test mice were less frequently dominant (67% WT versus 14% Mbnl2 KO dyads, *P* < 0.0001, binomial test). Stimulus mice also engaged in threat communication such as tail rattling or vocalization more frequently with *Mbnl2* KO test mice (7% WT versus 36% *Mbnl2* KO dyads, *P* = 0.0016, binomial test), further suggesting unclear territory or dominance relationships in *Mbnl2* KO dyads. Alterations in the response to social stimuli in *Mbnl2* KO mice on two genetic backgrounds, using two distinct assessments of social behavior, provide strong support for a social interaction deficit.

In contrast to WT mice, *Mbnl2* KO mice showed no significant preference for social novelty when presented with a familiar animal (stranger 1) and a novel animal (stranger 2) in the three-chamber apparatus (Fig. 8i). In support of an altered response to novelty more broadly, *Mbnl2* KO and *Dmpk*-(CTG)_480/480_ KI mice showed a reduction in nestlet shredding compared to WT littermate controls (Fig. 8j and Extended Data Fig. 9b)^72^. *Mbnl2* KO mice also showed a tendency toward reduced marble burying compared to WT mice (Extended Data Fig. 9c). Observed behavior patterns across these three tasks reveal altered responses to novelty in *Mbnl2* KO mice, which supports a previous finding of altered novel context processing in an *Mbnl2* constitutive inactivation mouse model^33^.

Collectively, these mouse behavioral results showed that either *Dmpk*-(CTG)_480/480_ expression or MBNL2 protein loss led to social interaction deficits and altered responses to novelty. No significant effect of sex was found in the analysis.

## Discussion

Here, we provide evidence that DMPK-CUG^exp^ expression and subsequent sequestration of MBNL splicing factors adversely impact the developmental splicing program of ASD-risk genes, including ASD-relevant miEs. The loss of MBNL protein function leads to social interaction deficits and restricted responses to novel social and nonsocial stimuli in mouse models. Thus, we demonstrate a new pathway that produces autistic traits, and propose a model where a gene-specific tandem repeat expansion induces an RNA-mediated gain of function, which leads to altered RNA splicing of multiple ASD-risk genes during brain development resulting in behavioral traits associated with autism (Fig. 8k).

Aberrant RNA splicing is a characteristic feature of the ASD brain^23–25^. Although a few splicing factors are downregulated in ASD, the mechanisms that connect genetic mutations to RNA mis-splicing remain enigmatic. For example, neuronal miE mis-splicing seen in approximately one-third of ASD cases, and their abnormal exclusion has been linked to downregulated *SRRM4* expression in ASD brains^23,26^. However, the molecular mechanism responsible for *SRRM4* reduction remains unknown. Here, we demonstrate that MBNL sequestration by expression of DMPK-CUG^exp^ mutations alters the splicing of ASD-risk gene transcripts, including ASD-relevant miEs. For example, the *ANK2* miE analyzed in this study is mis-spliced in both DM1 and ASD brains and is synergistically regulated by MBNL and SRRM proteins. While some miE transcripts can be directly bound and regulated by MBNL and SRRM proteins (such as Ank2), there may also be cases of combined direct and indirect effects, such as the recruitment of multiprotein regulatory complexes. Notably, like MBNL loss, SRRM4 haploinsufficiency, causes miE mis-splicing and a social deficit in mice^30^. Additionally, AS events in the DM1 brain can mimic ASD-associated variants. For example, *SCN2A* MXE mis-splicing results in a protein isoform differing by a single negatively charged amino acid (adult-to-fetal, D209N) in the extracellular loop of the Na_v_1.2 channel voltage-sensing domain. Accordingly, ASD-associated *SCN2A* variants reduce neuronal excitability^73–75^ similar to the fetal, or DM1, pattern that results in an MXE inclusion switch.

Our discovery opens up new therapeutic avenues for ASD by identifying a set of mis-spliced events in ASD-risk genes that could be corrected therapeutically. For example, tideglusib is a competitive inhibitor of glycogen synthase kinase 3 (GSK-3) with anti-inflammatory and neuroprotective properties. In preclinical studies, tideglusib reduces CUG^exp^ RNA levels and corrects aberrant splicing in DM1 models^76^. The role of mis-splicing in DM1-associated ASD is supported by recent clinical trial results that show tideglusib improves ASD symptoms in some treated DM1 children^77^. Several other therapeutic strategies are in clinical trials or under development that induce DMPK-CUG^exp^ RNA degradation and/or displace MBNL proteins from nuclear foci, including antisense oligonucleotides, RNA interference, small compounds, CRISPR/dCas9 and MBNL upregulation (reviewed in ref. ^78^). Although these strategies are being predominantly tested for efficacy in skeletal muscles, they pave the way for future studies to evaluate the effects of reducing CTG^exp^-induced RNA toxicity in the DM1 brain.

## Methods

### Mouse models

All relevant ethical regulations for animal testing and research were observed. This study received approval from the University of Florida Institutional Animal Care and Use Committee (IACUC). All animal procedures and endpoints were in accordance with IACUC guidelines and animals were killed in accordance with IACUC-approved protocols. The mouse strains B6-*Dmpk*-(CTG)_480_ KI, B6.129S1-*Mbnl1*^ΔE3/ΔE3^ (*Mbnl1* KO), B6.129S1-*Mbnl2*^ΔE2/ΔE2^ (B6.129-*Mbnl2* KO) and FVB-*Mbnl2*^ΔE2/ΔE2^ (FVB-*Mbnl2* KO) have been described previously^60,68–70^.

All behavioral analyses were performed between 8 weeks and 6 months of age, followed by brain collection. Mice were housed under specific-pathogen-free conditions. Humidity (50–70%) and temperature (70–75 °F) were controlled and the room was maintained on a 12-h light–dark cycle (lights off at 20:00). Mice were ear-notched and tail-snipped for identification and genotyping. Same-sex littermates were randomly group-caged at weaning (2–4 mice per cage) in cages with water and standard rodent chow available ad libitum. The mice remained in the same cage group throughout the behavioral experiments.

### Mouse behavioral assessments

Behavioral study designs were based on the ARRIVE guidelines (Animal Research Reporting of In Vivo Experiments)^80^. Studies were conducted by experimenters and observers blinded to the genotype of the animal, and all experimental cohorts contained mutant strains and WT littermate controls. Littermate groups originated from 11 litters from five distinct parental mating pairs, which were arranged into cohorts containing at least two animals of each genotype. Due to the need for genotype matching, animals were not randomized into groups. Animals within a cohort were tested on the same day, and additional cohorts were tested at approximately the same time of day on a subsequent day as required. Both male and female offspring were used, and sex was examined as a biological variable.

### Three-chamber test

The three-chamber test was used to assess sociability and the response to social novelty in mouse models. The rectangular apparatus consisted of three 20 × 40.5 × 22-cm chambers separated by clear Plexiglass walls. The walls had small doors that could be lifted or closed between phases to allow or prevent chamber access. Throughout the test, the center chamber remained empty and objects or stimulus (stranger) mice were placed in the left or right chambers.

The test mice that had their behavior analyzed were strains B6-WT (*n* = 11, *n*_XX_ = 7, *n*_XY_ = 4), B6-*Dmpk*-(CTG)_480/WT_ KI (*n* = 11, *n*_XX_ = 7, *n*_XY_ = 4), B6-*Dmpk*-(CTG)_480/480_ KI (*n* = 11, *n*_XX_ = 7, *n*_XY_ = 4), B6.129-WT (*n* = 12, *n*_XX_ = 9, *n*_XY_ = 3), or B6.129-*Mbnl2* KO (*n* = 12, *n*_XX_ = 9, *n*_XY_ = 3). All mice were between 8 weeks and 6 months old. Males and females were tested on different days. Same-sex, randomly group-caged littermates representing different genotypes were tested on the same day. The social stimulus mice (strangers) were matched in both age and sex to the test mouse. The test mice did not undergo any other experiments before being placed in the three-chambered social test. Similarly, the stimulus mice only were subject to being novel mice in the three-chambered test and were not involved in any other experiments. Wire cups were used to confine the stimulus mice, while allowing for social investigation by the test mouse. Before beginning, the two stimulus mice were habituated for 10 min in the inverted wire cups. During this habituation, we observed whether stimulus mice exhibited aggression or abnormal behaviors, such as excessive grooming, bar-biting and jumping, which could interfere with the test and provided grounds for their exclusion. None of the stimulus mice used in this study met these criteria for exclusion.

The test mice were habituated to the otherwise empty chambers for 10 min to allow us to assess whether they showed a preference for one side before any novel objects or animals were placed in the chamber.

For the sociability phase, an empty inverted wire cup was placed in one chamber, and an inverted wire cup with one stimulus animal (stranger) was placed in the opposite chamber. The chamber that contained the stimulus animal alternated with each test animal. The test mouse was placed in the center chamber and the doors were lifted to allow the mouse to explore all chambers for 10 min. The test animal was allowed to interact with the empty cup or with the social partner present for 10 min.

For the social novelty phase, the same stimulus animal (stranger 1) that was used in the sociability phase remained in place, and a novel stimulus mouse (stranger 2) was placed in the previously empty wire cup. The test mouse was placed in the center chamber, then allowed to explore all chambers for 10 min. This phase assessed whether the animal displayed more investigative behavior toward the novel stimulus mouse (stranger 2) or displayed a preference for the familiar mouse (stranger 1).

After the three phases of the social test were completed, the animals were placed back in their home cages. The interior of the chambers and the wire cups were sanitized with ethanol before proceeding with another test mouse. Illumination was kept even on both sides of the apparatus. The test was conducted in a quiet room with minimal visual distractions and was recorded using an overhead video camera. Each video specified the date, test animal ID and stimulus mice used.

Mouse video tracking during habituation phase was performed using ToxTrac (v.2.98)^81^. The recorded videos were observationally coded by blinded human raters using Behavioral Observation Research Interactive System (BORIS v.8.1.2) software^82^. Time in each chamber and the number of social and object interactions were coded during the sociability and social novelty phases, respectively. Social interactions were operationally defined as the test mouse sniffing the stimulus mouse, which could include nose-to-nose interaction, the test mouse sniffing any other part of the body of the stimulus mouse, or nose-to-cup interaction, and rearing on the wire cup containing the stimulus mouse^83^. Object interactions only applied to the sociability phase and were defined as sniffing or rearing on the wire cup that did not contain a stimulus mouse. During the social novelty phase, social interactions were coded for both the novel and familiar stranger. Twenty percent of the coded observations were randomly selected and independently scored by another researcher to determine the agreement between raters. A criterion of 85% or greater inter-observer agreement was established. Observations was scored as being in agreement if the behavioral scores for point events were recorded within 1 s of each other and (for longer-duration behaviors) if the start and stop times were recorded within 2 s. These parameters were set to account for the reaction time of the scorers. All the data included in this study met the criteria for 85% inter-rater agreement.

### Free dyadic social interaction test

Free dyadic social interaction was used as a second measure of social behavior to validate the results from the three-chamber tests. Dyadic social interaction was assessed in a rectangular apparatus measuring 30 × 45 × 20 cm. All mice were between 8 weeks and 6 months old. Test mice, FVB-WT (*n* = 15, *n*_XX_ = 9, *n*_XY_ = 6) or FVB-*Mbnl2* KO (*n* = 14, *n*_XX_ = 8, *n*_XY_ = 6), were first placed into the apparatus to habituate for 5 min, followed by the introduction of a novel stimulus mouse (WT C57BL6J) for 10 min. Test and stimulus mice were matched with respect to sex, age and weight. Test mice were assessed in five independent cohorts of FVB-*Mbnl2* KO and FVB-WT littermate controls. Mouse free dyadic social interaction was performed in a quiet room with minimal visual distractions and was recorded using an overhead video camera. Each video specified the date, test animal ID and stimulus mice used. The recorded videos were observationally coded by blinded human raters for social interactions between the test mouse and a novel stimulus mouse. Parameters tracked included the time stamp and duration of each interaction bout, and whether it was initiated by the test or stimulus mouse or reciprocally via nose-to-nose contact. An interaction bout was considered initiated when one mouse contacted the body or head of the other mouse, and bouts were considered to be maintained until the physical contact between the nose of one mouse and the other mouse (head, body or base of tail) was broken. The total number of bouts, average bout length and the total time spent interacting were calculated for each test animal. We also observed the nature of interactions, noting agonistic interactions, dominance behaviors of mounting and pinning, threat communication such as tail rattling or aggressive behaviors including thrusting, aggressive biting and fighting, as well as brief interactions (lasting less than 1 s) characterized by the retreat of either the test or stimulus mouse.

### Marble burying test

Marble burying was assessed in a rectangular apparatus measuring 30 × 45 × 20 cm filled with 5 cm of Sani Chip bedding, leveled evenly across the bottom of the apparatus. An array of 20 marbles (5 rows of 4 marbles each) was set on top of the bedding. All mice were between 8 weeks and 6 months old. Test mice, FVB-WT (*n* = 17, *n*_XX_ = 10, *n*_XY_ = 7) or FVB-*Mbnl2* KO (*n* = 14, *n*_XX_ = 7, *n*_XY_ = 7), were placed into the apparatus and left undisturbed for 30 min. The total number of marbles buried (at least two-thirds covered with bedding) was recorded, and before-and-after photographs were taken of the apparatus and marble array to assess the overall displacement of marbles and bedding. Data collection was performed blind to the mouse genotype.

### Nestlet shredding test

Nestlet shredding was assessed in a rectangular apparatus measuring 30 × 45 × 20 cm, containing one piece of unused nestlet placed two-thirds toward one end of the apparatus, on top of 0.5 cm of bedding. All mice were between 8 weeks and 6 months old. Test mice, FVB-WT (*n* = 17, *n*_XX_ = 11, *n*_XY_ = 6), FVB-*Mbnl2* KO (*n* = 16, *n*_XX_ = 7, *n*_XY_ = 9) or B6-WT (*n* = 13, *n*_XX_ = 8, *n*_XY_ = 5), B6-*Dmpk*-(CTG)_480/480_ KI (*n* = 10, *n*_XX_ = 6, *n*_XY_ = 4), were placed into the apparatus and left undisturbed for 30 min. The weight of the nestlet was measured before and after (following air drying for 24 h), and before and after photographs were taken of the apparatus and nestlet to assess displacement and extent of shredding or nest building. Data collection was performed blind to the mouse genotype. The extent of nestlet shredding was calculated as a percentage of the final dry weight to the original weight.

### Automated open field test

All mice were between 8 weeks and 6 months old. B6.129-WT (*n* = 11, *n*_XX_ = 8, *n*_XY_ = 3) and B6.129-*Mbnl2* KO (*n* = 12, *n*_XX_ = 9, *n*_XY_ = 3) mice were acclimated to the procedure room for approximately 2 h. For the open field test, mice were placed in the center of the darkened activity-monitoring 17 × 17-inch chamber (Med Associates) and mouse movement was traced for 30 min. Analysis was performed with Activity Monitor (MED Associates) software. For the final statistical analysis, only the 5–30 min interval was taken. Data collection was performed blind to the mouse genotype.

### Minigenes

EGFP-MBNL1-41, EGFP-MBNL2-38, Atp2a1-WT and Atp2a1-Δ minigenes were previously described^14,45^. Human pCMV6-SRRM4 with C-terminal Myc-DDK Tag was purchased from OriGene (RC219268).

Mouse normal and mutant *Atp2a1-Ank2* TGCT minigenes, as well as human normal and mutant *Atp2a1-ANK2* TGCT splicing minigenes, were generated by cloning DNA oligonucleotides between *Not*I and *Sal*I restriction sites in the Atp2a1-Δ minigene. DNA oligonucleotides (120 bp) contained selected TGCT(N)_3_TGCT(N)_13-18_TGCT/C or mutated GGCT(N)_3_TGAT(N)_13-18_TGTC sequences (substitutions are underlined), as well as *Not*I and *Sal*I restriction sites at 5′ and 3′ ends, respectively. DNA oligonucleotide sequences are listed in the key resources table. The complementary single-stranded DNA oligonucleotides (100 µM) were annealed in Annealing Buffer (10 mM Tris, pH 7.5, 50 mM NaCl and 1 mM EDTA) at 95 °C for 5 min, followed by cooling to 25 °C for 45 min. Annealed oligonucleotides were digested with *Not*I and *Sal*I restriction enzymes (New England Biolabs), purified using Clean-Up Concentrator kit (A&A Biotechnology) and ligated. The design of the hybrid *Atp2a1* minigenes preserves RNA structures within a thermodynamically stable region at the Atp2a1 insertion site^45^. Final splicing minigenes were read by Sanger sequencing.

Mouse normal *Atp2a1-Ank2* miE splicing minigenes were generated by cloning a digested PCR fragment between *Psh*AI and *Sal*I restriction sites in the Atp2a1-Δ minigene. PCR amplification was performed using CloneAmp HiFi PCR Premix (Takara), with N2a genomic DNA as a template. The PCR product was purified using Clean-Up Concentrator kit (A&A biotechnology) and digested with *Psh*AI and *Sal*I restriction enzymes (New England Biolabs). The inserts for mutant *Atp2a1-Ank2* miE splicing minigenes were generated by amplification of commercially synthesized DNA sequences (GenScript).

Srrm4-3xFlag was generated in two steps. First, the 3xFlag sequence was amplified from the PX458 plasmid (Addgene), purified on a column, digested with *Hin*dIII and *Eco*RI restriction enzymes (New England Biolabs), and inserted between *Hin*dIII and *Eco*RI restriction sites in pcDNA3.1(+) vector. Second, mouse Srrm4 was amplified from N2a cDNA, purified on a column, digested with *Eco*RI and *Xho*I (New England Biolabs), and inserted between *Eco*RI and *Xho*I restriction sites in 3xFlag-pcDNA3.1(+) plasmid.

Plasmid sequences were confirmed by Sanger sequencing. All PCR oligonucleotides and primers are listed in Supplementary Table 3.

### Cell culture and transfection

HeLa cells were cultured in Dulbecco’s modified Eagle medium (high-glucose, GlutaMAX supplement, pyruvate) supplemented with 10% fetal bovine serum (FBS) and 1× antibiotic and antimycotic. The Neuro2a (N2a) cell line (89121404, ECACC) was grown in minimum essential medium (EMEM) supplemented with 10% FBS, 1× MEM Non-essential Amino Acid Solution, 1× penicillin–streptomycin and 1× Normocin antimicrobial reagent. The cells were grown at 37 ˚C and 5% CO_2_.

Cells were seeded on 12-well plates filled with 1 ml of medium and allowed to grow up to 30% or 60% confluence before transfection for the 72-h and 48-h experiments, respectively. Cells were transfected with siRNA using Lipofectamine RNAiMAX Transfection Regent and with plasmids using Lipofectamine 3000 Transfection Regent according to the manufacturer’s protocol (Thermo Fisher Scientific). Genes were knocked down with ON-TARGETplus mouse Mbnl1 siRNA (50 nM, Dharmacon, L-065216-00-0020), ON-TARGETplus mouse Mbnl2 siRNA (25 nM, Dharmacon, L-065217-00-0010), Srrm3 (25 nM, Dharmacon, M-051745-01-0005), Srrm4 (25 nM, Dharmacon, M-058651-01-0005) and supplemented with control siRNA to reach 125 nM in total per well. The 125 nM control siRNA was used as a control (sense, /5Phos/rUrCrGrArArGrUrArUrUrCrCrGrCrGrUrArCrGdTdT; antisense, /5Phos/rCrGrUrArCrGrCrGrGrArArUrArCrUrUrCrGrAdTdT). For exogenous splicing analysis, HeLa or N2a cells were co-transfected with 200 ng of the indicated minigene construct and 500 ng of the EGFP-MBNL1-41, EGFP-MBNL2-38, SRRM4 or EGFP-expressing vector. The cells were collected 48 or 72 h after transfection.

### RNA isolation

Mouse tissues were homogenized in TRIzol (Ambion) with 1.5 mm zirconium beads in a Bead Ruptor 12 (OMNI International). Total RNA from mouse tissues and cells was isolated by using TRIzol Reagent (Thermo Fisher Scientific)/TRI Reagent (Sigma-Aldrich) and the Direct-zol RNA MiniPrep kit (Zymo Research)/Total RNA Zol-Out D kit (A&A Biotechnology) with on-column DNase digestion according to the manufacturer’s protocol.

### RT–PCR splicing and RNA steady-state level analysis

Total RNA (1–2 µg) was reverse transcribed using the GoScript Reverse Transcription System (Promega)/High-Capacity cDNA Reverse Transcription kit (Thermo Fisher Scientific) with Random Primers (Promega, Thermo Fisher Scientific) according to the manufacturer’s protocol. PCR was conducted using GoTaq G2 Flexi DNA Polymerase (Promega). PCR products of *Atp2a1-Ank2* miE minigene splicing were purified using Clean-Up Concentrator kit (A&A biotechnology) and digested overnight with *Kpn*I restriction enzyme (10 U; New England Biolabs). PCR products were resolved on 2% agarose gels stained with ethidium bromide and visualized on a Molecular Imager ChemiDoc XRS+ (Bio-Rad) or G:Box (Syngene) and analyzed using Image Lab v.6.1 (Bio-Rad), GeneTools v.4.3.9.0 (Syngene) or Multi Gauge v.3.0 (Fujifilm) software. All primers and PCR product sizes are listed in the key resources table.

Endogenous splicing of Ank2 miE and A3SS was analyzed by PCR with the 6-FAM-labeled forward primer followed by capillary electrophoresis. A 35-fold diluted labeled RT–PCR product (1 µl) from the RT–PCR reactions was mixed with 9 µl HiDi formamide containing GeneScan 600 LIZ Size Standard (Applied Biosystems) and denatured for 5 min at 95 °C, cooled and subjected to capillary electrophoresis on a 3130xl ABI Prism genetic analyzer (Applied Biosystems) using 36-cm capillaries and POP7 polymer. Peak size and area data were analyzed with PeakScanner software (Applied Biosystems). RT–PCR products were accurately identified with ±4-nt resolution. The relative height of fluorescent peaks for RT–PCR products with expected sizes for the alternatively spliced products were recorded.

For the RT–PCR validation of miEs from the datasets presented in Fig. 6f, we randomly selected eight out of 15 miEs that met the following criteria: (1) FDR < 0.05; (2) |ΔPSI| ≥ 20 for *Srrm* DKD dataset and |ΔPSI| ≥ 15 for *Mbnl* DKD dataset; and (3) miE length ≥18 bp, which allows to differentiate two isoforms on an agarose gel.

qRT–PCR was conducted with Maxima SYBR Green Rox (Thermo Fisher Scientific) on a QuantStudio 7 Flex instrument (Thermo Fisher Scientific). The experiments were carried out in triplicate amplification replicates for four experimental replicates. The relative quantification in gene expression was determined using the 2^–∆∆Ct^ method. The B2m-specific signal was used as a reference.

### Native RNA immunoprecipitation

Native RNA co-IP to detect RNAs that bind MBNL1 and SRRM4 directly or indirectly through a larger RNA-protein complex was performed using N2a cells transfected with EGFP-MBNL1-41-1xFlag, Srrm4-3xFlag or EGFP-C1 constructs and incubated for 48 h. Cells (2 × 10-cm plate per biological replicate) were collected and resuspended in RIPA buffer (150 mM NaCl, 50 mM Tris-HCl (pH 8.0), 1 mM EDTA, 0.5% NP-40, 0.5% Triton X-100, 0.5% sodium deoxycholate and 0.1% SDS) supplemented with EDTA-free Halt protease inhibitor, 1 mM dithiothreitol and 40 U RNasin (Promega), followed by incubation on ice for 30 min and then at −80 °C overnight. Anti-FLAG M2 magnetic beads (Merck) were first blocked with 4 µg BSA (Invitrogen) in RIPA buffer supplemented with Halt protease inhibitor on a rotating wheel overnight at 4 °C. The whole cell extract (WCE) was homogenized by passing the lysate five times through a 25-gauge needle and centrifuged at 450*g* for 10 min at 4 °C. Then, 15% of the WCE was collected for INPUT. The WCE was incubated with the beads on rotating wheel for 2 h at 4 °C followed by three rounds of washing with PBST (137 mM NaCl, 2.7 mM KCl, 8 mM Na_2_HPO_4_, 2 mM KH_2_PO_4_ and 0.02% Tween-20) and elution with acidic glycine solution. RNA was isolated using PureLink RNA mini kit combined with DNase treatment (Thermo Fisher). cDNA was prepared using High-Capacity Reverse transcription kit (Thermo Fisher) following the manufacturer’s protocol. The pre-mRNA analyses were conducted with qRT–PCR using primers binding in close proximity to miEs and potential MBNL and SRRM-binding sites and amplifying up to 300-bp-long regions. Randomly selected Nfat5 and Fmr1 pre-mRNAs constitute negative controls and cutoff points for studied RNAs. RIP qRT–PCR results were represented as a relative IP/INPUT =  2^x^; *x* = (Ct_IP:MBNL1 or SRRM4_ − Ct_INPUT:MBNL1 or SRRM4_) − (Ct_IP:GFP_ − Ct_INPUT:GFP_) normalized to two negative controls ((IP/INPUT Nfat5 + IP/INPUT Fmr1)/2).

### Combinatory effect of MBNL and SRRM proteins

To calculate the combinatory effect for SRRM and MBNL proteins, we utilized the formula CI = (EA + EB-EA × EB)/EAB; where CI is combination index. For concordant changes EB is ∆PSI *Srrm* DKD, EA is ∆PSI *Mbnl* DKD and EAB is ∆PSI *Srrm*;*Mbnl* QKD. For opposite changes the PSI value was used. CI < 1 or CI > 1 indicates a synergistic or antagonistic combination, respectively.

### Quantification of in vitro MBNL1–Ank2 RNA interaction

A biochemical assay based on double-membrane filtration analysis was performed as previously described^44^. The *K*_d_ of the recombinant MBNL1–RNA complexes was calculated based on the signal on nitrocellulose from three experimental replicates in GraphPad using normalization to 0–1 range followed by one site specific binding curve equation, *Y* = *B*_max_ × *X*/(*K*_d_ + *X*).

Ank2 template: gGGAACCTTGGTCTTCCTGGGTTCATTCGCATTTCCCTGCTTCCTGCTCATCACAGCTCACTGCTCACTGTGTGTGTTTGTGTGTGTGTG;

Ank2-mut template: gGGAACCTTGGTCTTCCTGGGTTCATTCGCATTTCCCGGCTTCCTGATCATCACAGCTCACTGTTCACTGTGTGTGTTTGTGTGTGTGTG.

### RNA-seq and CLIP-seq analysis

All RNA-seq and CLIP-seq accession numbers and references to the original research articles are listed in Supplementary Table 2. Reads were aligned to the human hg38 or mouse mm10 genomes using STAR (v.2.7.5c)^84^. Splicing analysis was performed using rMATS (v.4.1.0)^85^. Criteria for abnormal splicing include an absolute mean of differential PSI values (|ΔPSI|) > 0.1 and FDR < 0.05. Sashimi plots were generated using ggsashimi.py script^86^. Median coverage was used to generate the plot (-A median). The total numbers of junction reads are shown. The introns were compressed for better representation (--shrink). Transcript expression quantification was performed using Salmon (v.1.1)^87^, and differential gene expression analysis was performed using DESeq2 (v.1.32.C)^88^. Mapped RNA-seq reads were counted using featureCounts (v.1.6.2)^89^.

### Gene set enrichment analysis

The 38 gene sets and available databases were previously used to assess the relevance of particular genes to ASD (Supplementary Table 1). We carried out a gene set enrichment analysis for mis-spliced genes in our study. The OR of mis-spliced events in ASD-risk genes was calculated over mis-spliced events in other genes, using a Fisher’s exact test. The 95% confidence intervals were also determined. FDR values were estimated from *P* values using the Benjamini–Hochberg procedure.

### MBNL and RBFOX binding motif enrichment analysis

The distance to the nearest MBNL-binding motif pattern (YGCY or YGCY(N)_0–5_YGCY; Y indicates pyrimidine) and RBFOX binding pattern (GCAYG) was calculated for all mis-spliced (|ΔPSI|> 0.1, FDR < 0.05,) SE events. For all SE events that have an MBNL/RBFOX binding motif within 250 bp, we calculated the enrichment of mis-spliced exons in ASD-risk genes over other mis-spliced exons (Fig. 1h). Alternatively (Fig. 5c), genes were annotated as mis-spliced or not mis-spliced, and then we calculated the enrichment of mis-spliced ASD-risk genes over other genes. Fisher’s exact test was used to determine the enrichment and 95% confidence interval. FDR values were estimated from *P* values using the Benjamini–Hochberg procedure.

### Human and mouse AS overlap

Coordinates of AS events from DM1 patient brains were transferred to mouse genome (mm10) coordinates using the UCSC genome browser LiftOver feature (https://www.genome.ucsc.edu/cgi-bin/hgLiftOver). SE events from DM1, *Mbnl* DKO and *Mbnl2* KO were overlapped, and the percentage of overlapping events annotated in ASD-risk genes were calculated (Fig. 4g).

### Gene expression database

Gene expression was retrieved from Evo-devo mammalian organs dataset^55^ and the Genotype-Tissue Expression (accession no. phs000424.v8.p2).

### RNA structure prediction

The normal and mutant 179-nt long Ank2 RNA sequences, carrying MBNL and SRRM-binding sites, were run through the mfold secondary structure prediction software program (http://www.unafold.org/mfold) with default parameters.

### Protein structure prediction

The modeled structures of mouse proteins up to 50 aa or 214 aa for SHANK3 were predicted using the UCSF ChimeraX AlphaFold tool with the use of ColabFold, an optimized version of AlphaFold2 with default parameters^90,91^. Protein fragments used for structure modeling with miE-encoded residues are underlined.

Ank2 without miE-encoded sequence: GMNYLRYSLEGGRSDSLRSFSSDRSHTLSHASYLRDSAMIDD

Ank2 with miE-encoded sequence: GMNYLRYSLEGGRSDSTIPSSDRSHTLSHASYLRDSAMIDD

Shank3 without miE-encoded sequence: GGLGSLLDPAKKSPIAAARLFSSLGELSTISAQRSPGGPGGGASYSVRPSGRYPVARRAPSPVKPASLERVEGLGAGVGGAGRPFGLTPPTILKSSSLSIPHEPKEVRFVVRSVSARSRSPSPSPLPSPSPGSGPSAGPRRPFQQKPLQLWSKFDVGDWLESIHLGEHRDRFEDHEIEGAHLPALTKEDFVELGVTRVGHRMNIERALRQLDGS

Shank3 with miE-encoded sequence: GGLGSLLDPAKKSPIAAARCAVVPSAGCALQQPR

Nrxn1 without miE-encoded sequence: RLPDLISDALFCNGQIERGCEGPSTTCQEDSCSNQGVCLQQ

Nrxn1 with miE-encoded sequence: RLPDLISDALFCNGQIERGCEVALMKADLQGPSTTCQEDSCSNQGVCLQQ

Dmd without miE-encoded sequence: TGLEEVMEQLNNSFPSSRGHNVGSLFHMADDLGRAMESLVSVMTDEEGAE

Dmd with miE-encoded sequence: TGLEEVMEQLNNSFPSSRGRNAPGKPMREDTM

### Post-translational modifications

Post-translational modifications were retrieved from PhosphoSitePlus (v.6.7.1.1; www.phosphosite.org).

### Allen Mouse Brain Atlas

The Allen Mouse Brain Atlas was accessed from mouse.brain-map.org. Experiments were performed on P56d old male C57BL/6J mice. A detailed description of the in situ hybridization procedure and informatics data processing is at https://community.brain-map.org/t/documentation-allen-mouse-brain-atlas/2846.

### Group size

Group size determinations for behavioral experiments were based on assuming power of 0.8, *α* = 0.05, with effect sizes estimated based on our previous studies, using G*Power (v.3.1) software. We analyzed sex- and age-matched groups. The *n* values represent biological replicates. RNA-seq maximum group sizes and sample characteristics were predetermined as we used data from repositories. For splicing analyses, no statistical methods were used to predetermine sample sizes, but our sample sizes are similar to those reported in previous publications^14,44,60^.

### Data exclusions

For the PsychENCODE ASD splicing analysis, we selected RNA-seq samples that recapitulated the age distribution of the DM1 BA10 RNA-seq samples (DM1, median = 56 y, min = 39 y, max = 77 y; ASD, median = 51 y, min = 38 y, max = 67 y). Some RT–PCR results for endogenous AS events were not included due to very low or no amplification. We had a surplus of behavioral data for WT and *Dmpk*-(CTG)_480/WT_ KI mice due to a skewed offspring distribution. Mice were randomized into cages, and experimenters were blinded to their genotypes during the three-chamber test and observational coding. To match *n* = 11 for the *Dmpk*-(CTG)_480/480_ group, we randomly selected 11 sex-matched results for WTs and *Dmpk*-(CTG)_480/WT_ KIs mice (Fig. 8b). One animal was excluded from the free dyadic social interaction test due to excessive aggressive behavior that prevented meaningful interpretation of results (Fig. 8h). Although balanced animal groups were planned, Mendelian ratios were not achieved for the experimental animal sex distribution. Furthermore, during extended behavioral testing some animals died of unknown causes resulting in uneven group numbers for some assessments.

### Statistical analysis

Whole-transcriptome statistical analysis for splicing and gene expression was performed using rMATS (v.4.1.0)^85^ and DESeq2 (v.1.32.C)^88^, respectively. The OR was calculated using the ‘epitools’ package in R, and the statistical significance was determined based on Fisher’s exact test followed by the multiple comparison correction using the FDR method. Other statistical analyses were performed using GraphPad Prism (v.9.5.1). The normal distribution was assessed by the Shapiro–Wilk test followed by parametric or nonparametric tests and the post hoc test for multiple comparisons. Details are specified in the figure legends. Graphs were generated in R using the ‘ggplot2’ package and GraphPad Prism (v.9.5.1) software.

### Reporting summary

Further information on research design is available in the Nature Portfolio Reporting Summary linked to this article.

## Online content

Any methods, additional references, Nature Portfolio reporting summaries, source data, extended data, supplementary information, acknowledgements, peer review information; details of author contributions and competing interests; and statements of data and code availability are available at 10.1038/s41593-025-01943-0.

## Supplementary information

Reporting Summary

Supplementary Tables 1–3 Supplementary Tables 1–3.

## Source data

Source Data Fig. 1 Statistical source data file.

Source Data Fig. 2 Statistical source data file.

Source Data Fig. 3 Statistical source data file.

Source Data Fig. 4 Statistical source data file.

Source Data Fig. 5 Statistical source data file.

Source Data Fig. 6 Statistical source data file.

Source Data Fig. 7 Statistical source data file.

Source Data Fig. 8 Statistical source data file.

Source Data Extended Data Fig. 1 Statistical source data file.

Source Data Extended Data Fig. 2 Statistical source data file.

Source Data Extended Data Fig. 3 Statistical source data file.

Source Data Extended Data Fig. 4 Statistical source data file.

Source Data Extended Data Fig. 5 Statistical source data file.

Source Data Extended Data Fig. 6 Statistical source data file.

Source Data Extended Data Fig. 7 Statistical source data file.

Source Data Extended Data Fig. 8 Statistical source data file.

Source Data Extended Data Fig. 9 Statistical source data file.

Source Data Extended Data Fig. 10 Unprocessed gels.

## Data Availability

Requests for further information or resources and reagents should be directed to the lead author, Ł.J.S. (lukasz.sznajder@unlv.edu). Previously published RNA-seq and CLIP-seq data were used for this work (GSE157428, GSE201898, GSE36710, GSE130905, SRP055008, SRP142522, GSE38497, GSE67828, GSE112600 and GSE57278)^35,47,51,52,56,57,60,63,64,92^. The run numbers are listed in Supplementary Table 2. The restricted-access ASD RNA-seq data are available at Synapse, and access can be granted by the National Institute of Mental Health Repository and Genomics Resource. The sources of all ASD-risk gene datasets are listed in the Supplementary Table 1. Gene expression was retrieved from Evo-devo mammalian organs dataset^55^ and the Genotype-Tissue Expression (accession no. phs000424.v8.p2). Post-translational modifications were retrieved from PhosphoSitePlus (v.6.7.1.1; www.phosphosite.org). The Allen Mouse Brain Atlas was accessed from mouse.brain-map.org; Mbnl1 (mouse.brain-map.org/gene/show/36037) and Mbnl2 (mouse.brain-map.org/gene/show/69724)^79^. Additional information required to reanalyze the data reported here is available from the lead contact upon request. Source data are provided with this paper.

## Code availability

The code used in this study can be accessed via GitHub at https://github.com/mahreenkn/DM1-Autism-RNABinding.
