# Supplementary material for: Autism-related traits in myotonic dystrophy type 1 model mice are due to MBNL sequestration and RNA mis-splicing of autism-risk genes
Source: Nat Neurosci. 2025 Apr 21;28(6):1199–212. doi: 10.1038/s41593-025-01943-0 (PMC12148930; doi:10.1038/s41593-025-01943-0)
Supplement: Supplementary file 1 — Reporting Summary [file 41593_2025_1943_MOESM1_ESM.pdf]

nature portfolio

Corresponding author(s): Łukasz J. Sznajder

Last updated by author(s): 02/25/2025

Reporting Summary

Nature Portfolio wishes to improve the reproducibility of the work that we publish. This form provides structure for consistency and transparency in reporting. For further information on Nature Portfolio policies, see our Editorial Policies and the Editorial Policy Checklist.

Statistics

For all statistical analyses, confirm that the following items are present in the figure legend, table legend, main text, or Methods section.

n/a	Confirmed	
☐	☒	The exact sample size (*n*) for each experimental group/condition, given as a discrete number and unit of measurement
☐	☒	A statement on whether measurements were taken from distinct samples or whether the same sample was measured repeatedly
☐	☒	The statistical test(s) used AND whether they are one- or two-sided<br>*Only common tests should be described solely by name; describe more complex techniques in the Methods section.*
☐	☒	A description of all covariates tested
☐	☒	A description of any assumptions or corrections, such as tests of normality and adjustment for multiple comparisons
☐	☒	A full description of the statistical parameters including central tendency (e.g. means) or other basic estimates (e.g. regression coefficient) AND variation (e.g. standard deviation) or associated estimates of uncertainty (e.g. confidence intervals)
☐	☒	For null hypothesis testing, the test statistic (e.g. *F*, *t*, *r*) with confidence intervals, effect sizes, degrees of freedom and *P* value noted<br>*Give P values as exact values whenever suitable.*
☒	☐	For Bayesian analysis, information on the choice of priors and Markov chain Monte Carlo settings
☒	☐	For hierarchical and complex designs, identification of the appropriate level for tests and full reporting of outcomes
☐	☒	Estimates of effect sizes (e.g. Cohen's *d*, Pearson's *r*), indicating how they were calculated

Our web collection on statistics for biologists contains articles on many of the points above.

Software and code

Policy information about availability of computer code

Data collection	PCR products were resolved on agarose gels stained with ethidium bromide and visualized on a Molecular Imager ChemiDoc XRS + (BioRad) or G:Box (Syngene). Quantitative RT-PCR (qRT-PCR) was conducted with Maxima SYBR Green Rox (Thermo Fisher Scientific) on a QuantStudio 7 Flex instrument (Thermo Fisher Scientific). Data collected by the automated open field system were extracted using Activity Monitor software (MED Associates). Mice were recorded by the overhang video camera during the three chamber and free dyadic social interaction tests.
Data analysis	The recorded videos were observationally coded by human raters using Behavioral Observation Research Interactive System (BORIS v 8.1.2) software. Mouse video tracking during habituation phase was performed using ToxTrac (v 2.98). The intensity of the PCR bands was analyzed using Image Lab v 6.1 (BioRad), GeneTools v 4.3.9.0 (Syngene), or Multi Gauge v 3.0 (Fujifilm) software. RNA-seq reads were aligned to the human hg38 or mouse mm10 genomes using STAR (v 2.7.5c). Splicing analysis was performed using rMATS (v 4.1.0). Criteria for abnormal splicing include an absolute mean of differential percent spliced-in values (|deltaPSI|) > 0.1 and a false discovery rate (FDR) < 0.05. Sashimi plots were generated using ggsashimi.py script. Median coverage was used to generate the plot (-A median). The total numbers of junction reads are shown. The introns were compressed for better representation (--shrink). Transcript expression quantification was performed using Salmon (v 1.1), and differential gene expression analysis was performed using DESeq2 (v 1.32.C). Mapped RNA-seq reads were counted using featureCounts (v 1.6.2). The odds ratio (OR) was calculated using 'epitools' package in R, and the statistical significance was determined based on Fisher's exact test followed by the multiple comparison correction using the FDR method. Other statistical analyses were performed using GraphPad Prism (v 9.5.1). The normal distribution was assessed by the Shapiro–Wilk test followed by parametric or nonparametric tests and the post hoc test for multiple comparisons. Graphs were generated in R using the 'ggplot2' package and GraphPad Prism (v 9.5.1) software. The code used in this study can be accessed via GitHub at (https://github.com/mahreenkn/DM1-Autism-RNABinding).

For manuscripts utilizing custom algorithms or software that are central to the research but not yet described in published literature, software must be made available to editors and reviewers. We strongly encourage code deposition in a community repository (e.g. GitHub). See the Nature Portfolio guidelines for submitting code & software for further information.

Data

Policy information about availability of data

All manuscripts must include a data availability statement. This statement should provide the following information, where applicable:

- Accession codes, unique identifiers, or web links for publicly available datasets
- A description of any restrictions on data availability
- For clinical datasets or third party data, please ensure that the statement adheres to our policy

Previously published RNA-seq and CLIP-seq data were used for this work (GSE157428, GSE201898, GSE36710, GSE130905, SRP055008, SRP142522, GSE38497, GSE67828, GSE112600, GSE57278). The run numbers are listed in Supplementary Table 2. The restricted-access ASD RNA-seq data are available at Synapse, and access can be granted by the NIMH Repository & Genomics Resource. The sources of all ASD-risk gene datasets are listed in the Supplementary Table 1. Gene expression was retrieved from Evo-devo mammalian organs dataset and the Genotype-Tissue Expression (GTEx; accession number phs000424.v8.p2). Post-translational modifications were retrieved from PhosphoSitePlus (v 6.7.1.1; www.phosphosite.org). Mouse Brain Atlas was accessed from mouse.brain-map.org; Mbnl1 (mouse.brain-map.org/gene/show/36037), Mbnl2 (mouse.brain-map.org/gene/show/69724).

Research involving human participants, their data, or biological material

Policy information about studies with human participants or human data. See also policy information about sex, gender (identity/presentation), and sexual orientation and race, ethnicity and racism.

Reporting on sex and gender	Supplementary Table 2 contains information about the biological gender.
Reporting on race, ethnicity, or other socially relevant groupings	This study used de-identified postmortem human RNA-seq brain samples, and information about race, ethnicity, and social group was not available.
Population characteristics	Supplementary Table 2 file contains information about the age of death, diagnosis, and genetic mutation.
Recruitment	There was no recruitment.
Ethics oversight	This study used RNA-seq data available at GEO. Access to ASD RNA-seq data deposited at Synapse was granted by the NIMH Repository & Genomics Resource.

Note that full information on the approval of the study protocol must also be provided in the manuscript.

Field-specific reporting

Please select the one below that is the best fit for your research. If you are not sure, read the appropriate sections before making your selection.

☒ Life sciences ☐ Behavioural & social sciences ☐ Ecological, evolutionary & environmental sciences

For a reference copy of the document with all sections, see nature.com/documents/nr-reporting-summary-flat.pdf

Life sciences study design

All studies must disclose on these points even when the disclosure is negative.

Sample size	Animal group size determination for behavioral experiments was based on assuming power = 0.8, α = 0.05 with effect sizes estimated based on our previous studies using G*Power (v 3.1) software. Human postmortem brain group sizes and sample characteristics were predetermined by RNA-seq data availability. Group sizes for RT-PCR splicing analysis in mouse and cell models were estimated based on our previous studies.
Data exclusions	For the PsychENCODE ASD splicing analysis, we selected RNA-seq samples that recapitulated the age distribution of the DM1 BA10 RNA-seq samples (DM1: median=56y, min=39y, max=77y; ASD: median=51y, min=38y, max=67y). Some RT-PCR results for endogenous AS events were not included due to very low or no amplification. We had a surplus of behavioral data for WT and Dmpk-(CTG)480/WT KI mice due to a skewed offspring distribution. Mice were randomized into cages, and experimenters were blinded to their genotypes during the three-chamber test and observational coding. To match N=11 for the Dmpk-(CTG)480/480 group, we randomly selected 11 sex-matched results for WTs and Dmpk-(CTG)480/WT KIs mice (Fig.8b). One animal was excluded from the free dyadic social interaction test due to excessive aggressive behavior that prevented meaningful interpretation of results (Fig.8h). Although balanced animal groups were planned, Mendelian ratios were not achieved for the experimental animal sex distribution. Furthermore, during extended behavioral testing some animals died of unknown causes resulting in uneven group numbers for some assessments.
Replication	We attempted to replicate most of the computational analyses and molecular biology experiments included in the manuscript, and all were successful, with independent researchers obtaining the same results.
Randomization	Same-sex littermates were randomly group-caged at weaning. RT-PCR samples were not randomized because doing so would introduce unnecessary complexity, whereas the goal is to control for experimental variables and minimize technical variability.

Blinding	The experimenters were blinded to the tested mice genotypes at the time of testing and video tracking analysis. We were not blinded for RNA-seq and RT-PCR analysis due to the standardized procedures and the limited impact of researcher bias on data acquisition.

Reporting for specific materials, systems and methods

We require information from authors about some types of materials, experimental systems and methods used in many studies. Here, indicate whether each material, system or method listed is relevant to your study. If you are not sure if a list item applies to your research, read the appropriate section before selecting a response.

Materials & experimental systems

n/a	Involved in the study
☒	☐ Antibodies
☐	☒ Eukaryotic cell lines
☒	☐ Palaeontology and archaeology
☐	☒ Animals and other organisms
☒	☐ Clinical data
☒	☐ Dual use research of concern
☒	☐ Plants

Methods

n/a	Involved in the study
☒	☐ ChIP-seq
☒	☐ Flow cytometry
☒	☐ MRI-based neuroimaging

Eukaryotic cell lines

Policy information about cell lines and Sex and Gender in Research

Cell line source(s)	HeLa (CCL-2; ATCC) and Neuro2a (CCL-131; ATCC)
Authentication	Cell lines were not authenticated.
Mycoplasma contamination	Cell lines were not tested for mycoplasma contamination.
Commonly misidentified lines (See ICLAC register)	No misidenified cell lines were used in the study.

Animals and other research organisms

Policy information about studies involving animals; ARRIVE guidelines recommended for reporting animal research, and Sex and Gender in Research

Laboratory animals	Mouse strains: B6-Dmpk-(CTG)480 KI, B6.129S1-Mbnl1deltaE3/deltaE3 (Mbnl1 KO), B6.129S1-Mbnl2deltaE2/deltaE2 (B6.129-Mbnl2 KO), and FVB-Mbnl2deltaE2/deltaE2 (FVB-Mbnl2 KO). All behavioral analyses were performed between 8 weeks and 6 months of age followed by brain harvesting.
Wild animals	The study did not involve wild animals.
Reporting on sex	We analyzed both males and females in our study. We did not perform sex-based analyses because it is unknown whether autistic traits in myotonic dystrophy are predominant in one sex.
Field-collected samples	This study did not involve samples collected from the field.
Ethics oversight	All relevant ethical regulations for animal testing and research were observed. This study received approval from the University of Florida Institutional Animal Care and Use Committee (IACUC). All animal procedures and endpoints were in accordance with IACUC guidelines, and animals were sacrificed in accordance with IACUC-approved protocols.

Note that full information on the approval of the study protocol must also be provided in the manuscript.
