# Supplementary material for: Autism-related traits in myotonic dystrophy type 1 model mice are due to MBNL sequestration and RNA mis-splicing of autism-risk genes
Source: Nat Neurosci. 2025 Apr 21;28(6):1199–212. doi: 10.1038/s41593-025-01943-0 (PMC12148930; doi:10.1038/s41593-025-01943-0)

Source Data File

Autism-related traits in myotonic dystrophy type 1 model mice are due to MBNL sequestration and RNA mis-splicing of autism-risk genes

Sznajder *et al.*

Data for Figure 4b and Extended Data Figure 4d

Figure 4b
Frontal cortex, Scn2a MXE
WT | *Mbnl2* KO

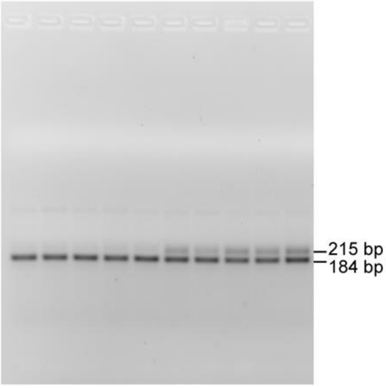

Figure 4b
Hippocampus, Scn2a MXE
WT | *Mbnl2* KO

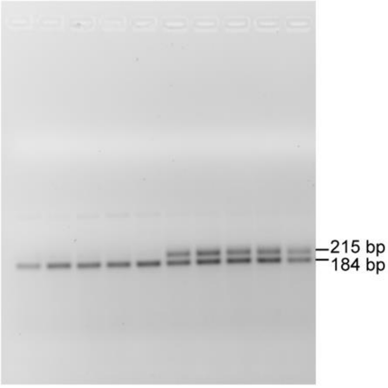

Extended Data Figure 4d
Cerebellum, Scn2a MXE
WT | *Mbnl2* KO

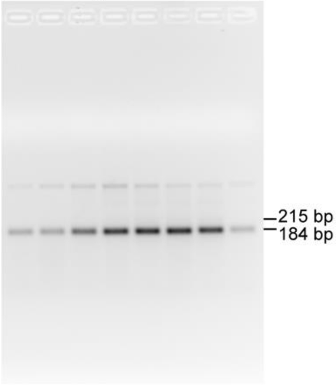

Figure 4b
Frontal cortex, Nrxn1 miE
WT | *Mbnl2* KO

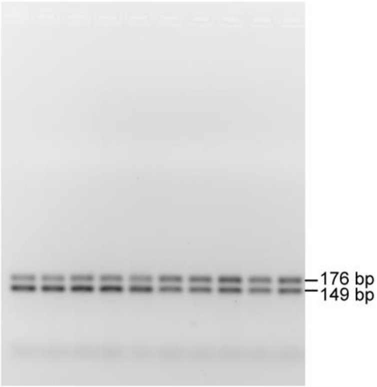

Figure 4b
Hippocampus, Nrxn1 miE
WT | *Mbnl2* KO

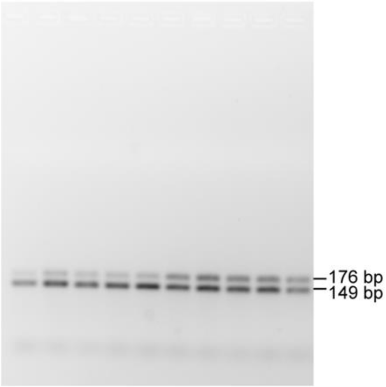

Extended Data Figure 4d
Cerebellum, Nrxn1 miE
WT | *Mbnl2* KO

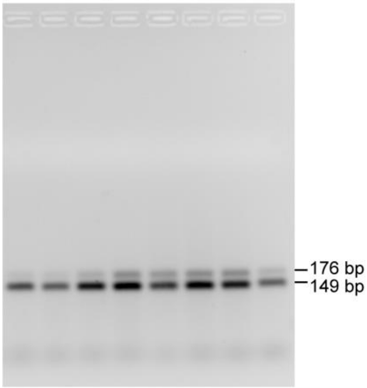

Figure 4b
Frontal cortex, Shank3 miE
WT | *Mbnl2* KO

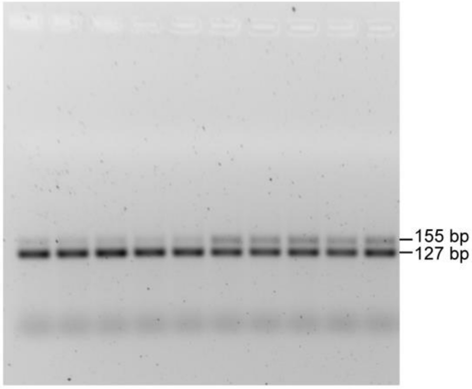

Figure 4b
Hippocampus, Shank3 miE
WT | *Mbnl2* KO

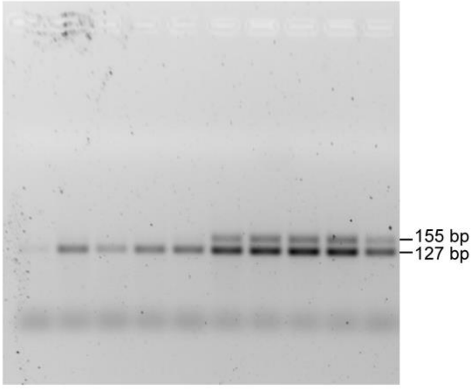

Data for Figure 5h and Extended Data Figure 5e

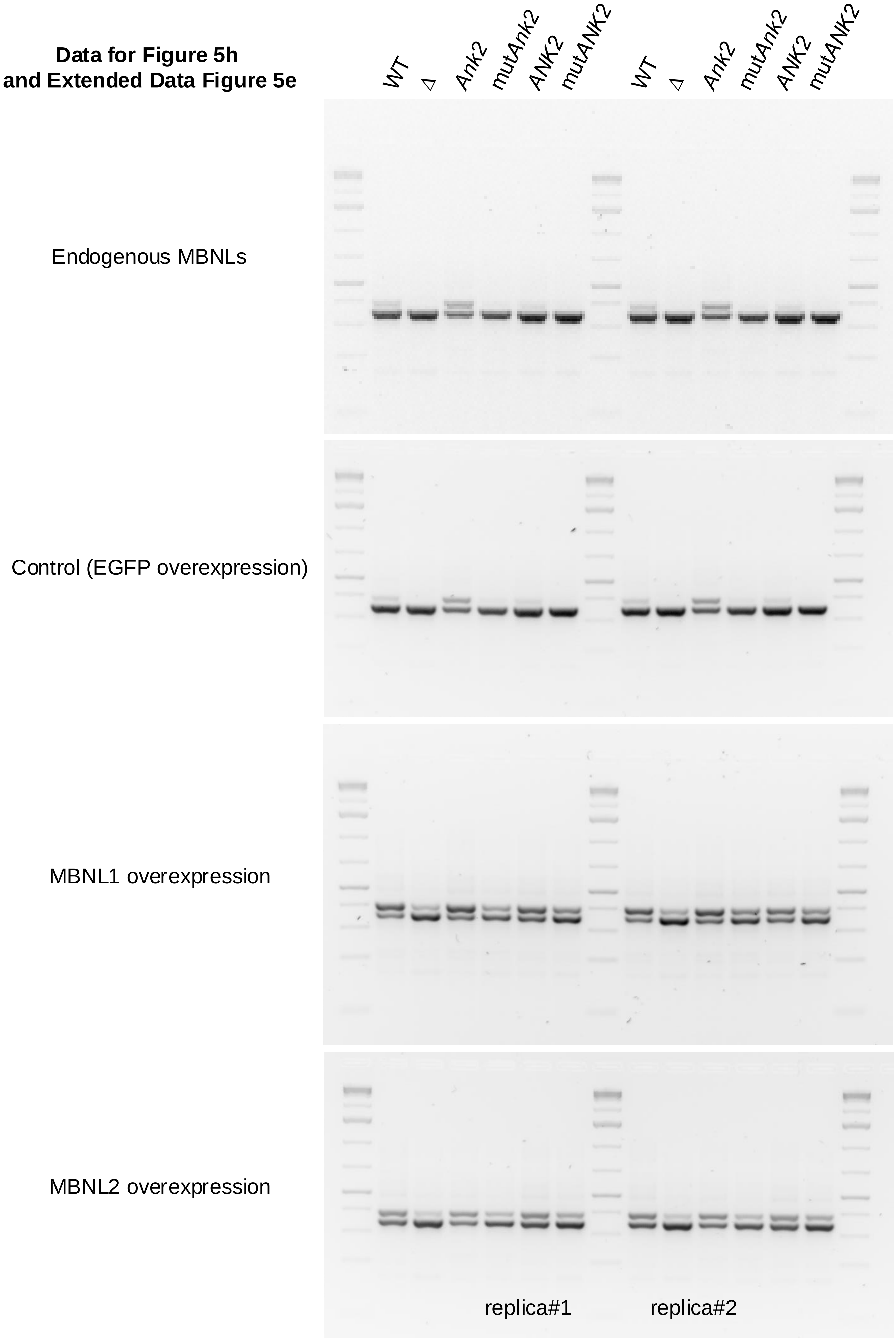

Data for Figure 5h and Extended Data Figure 5e

Non-treated control (NTC) – Endogenous MBNLs

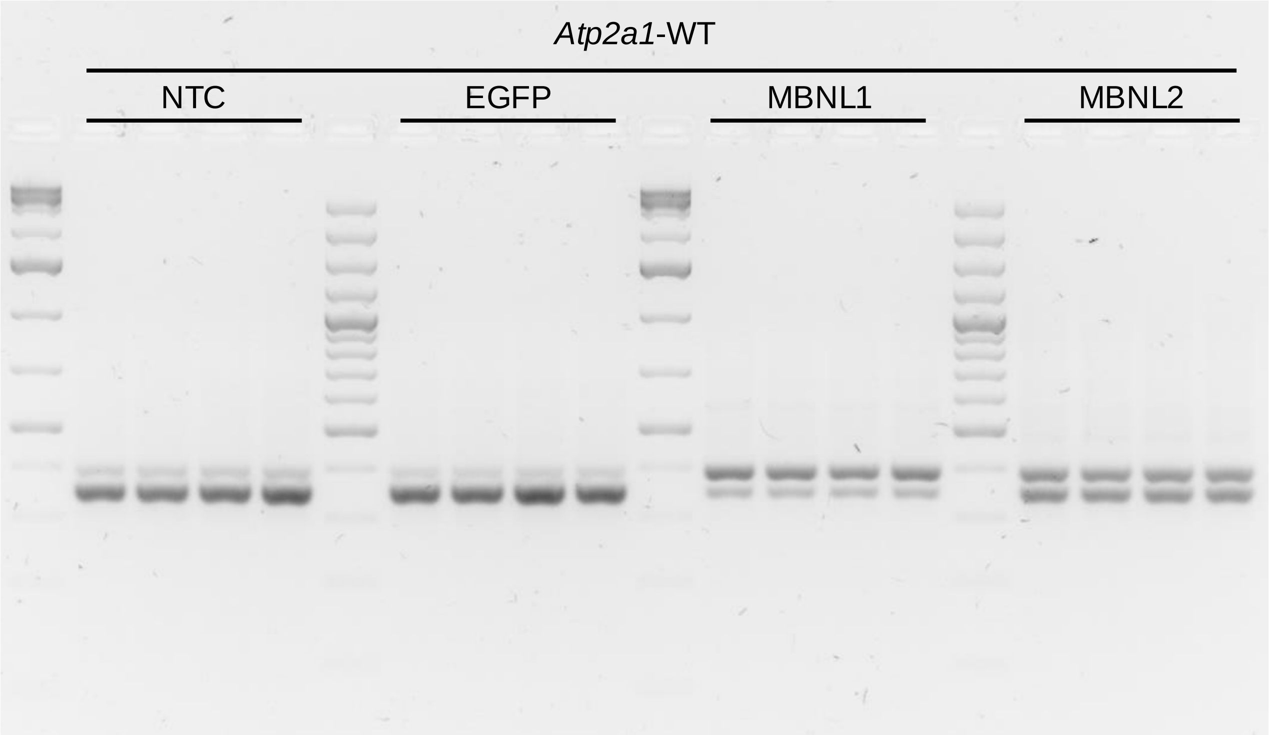

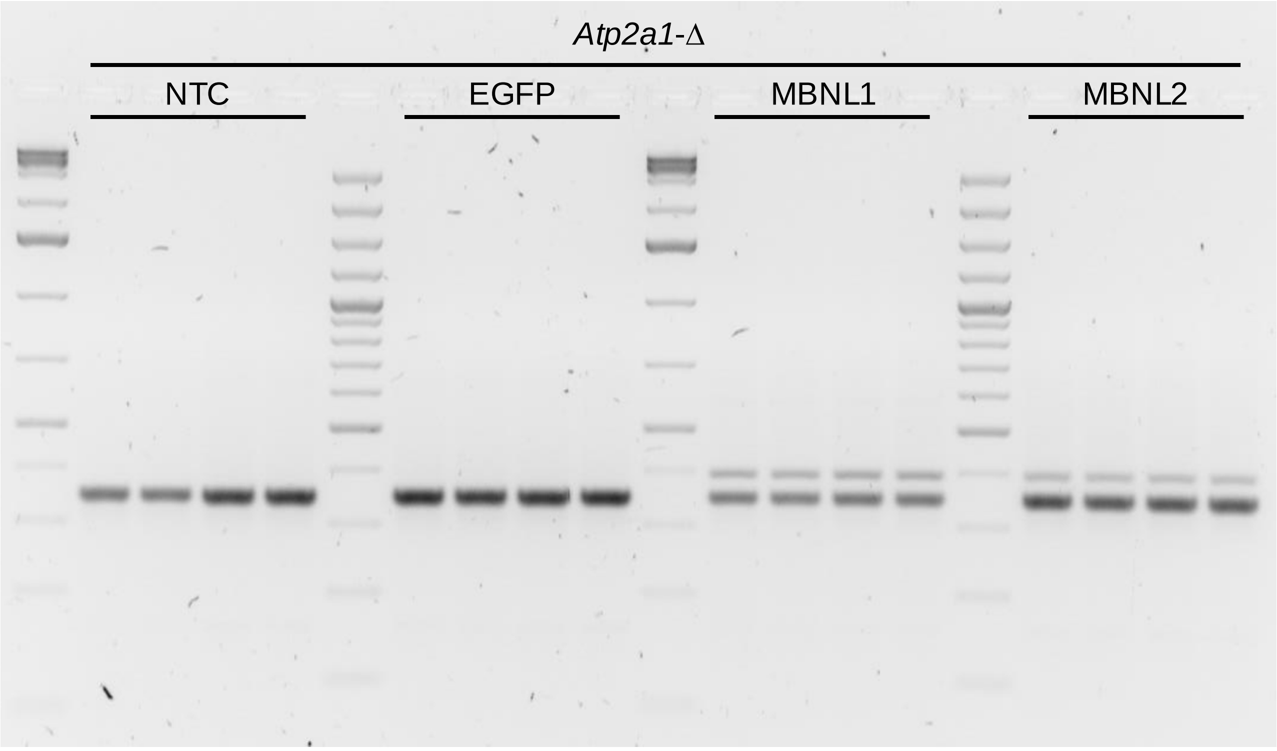

Data for Figure 5h and Extended Data Figure 5e

Non-treated control (NTC) – Endogenous MBNLs

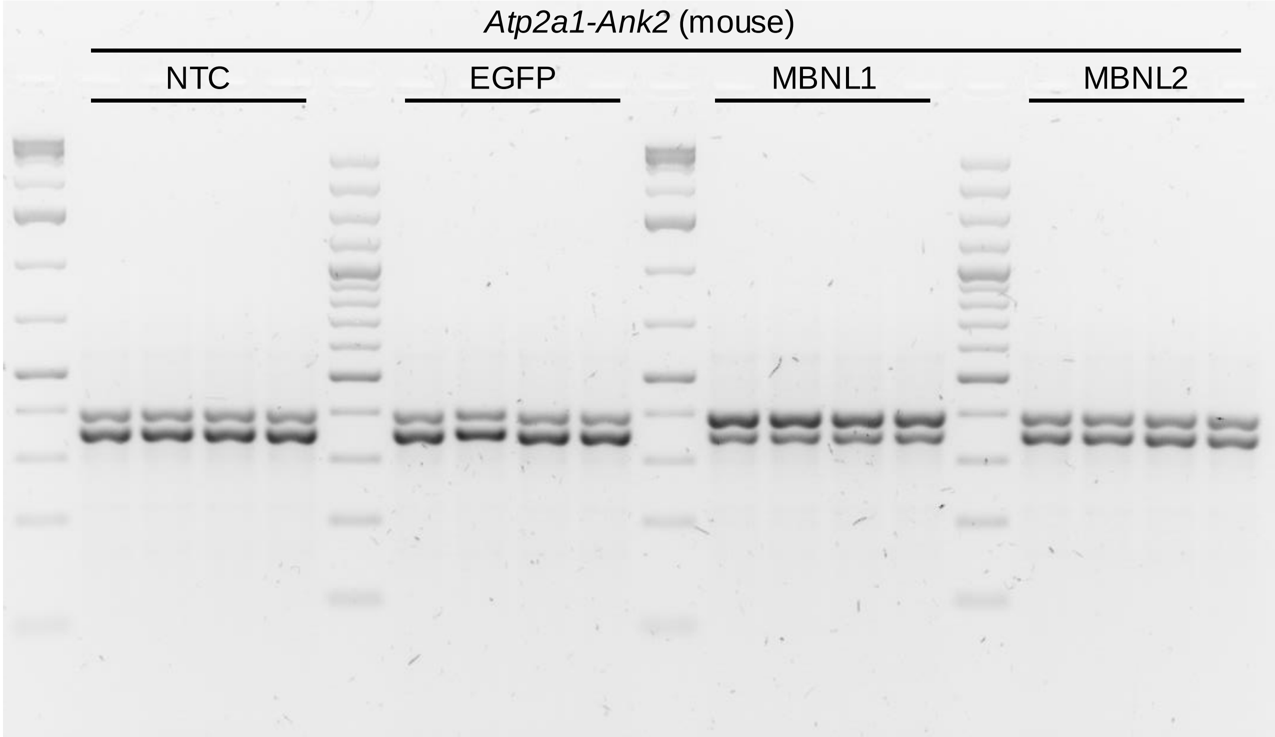

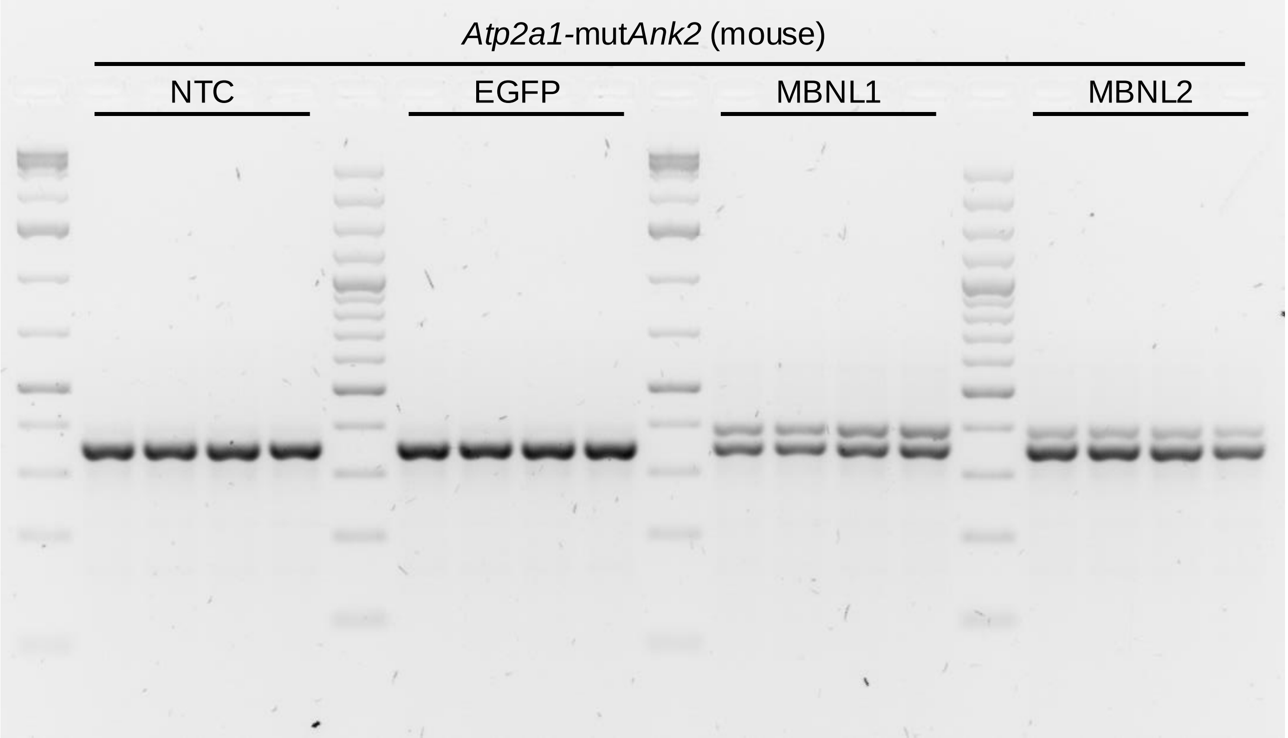

Data for Figure 5h and Extended Data Figure 5e

Non-treated control (NTC) – Endogenous MBNLs

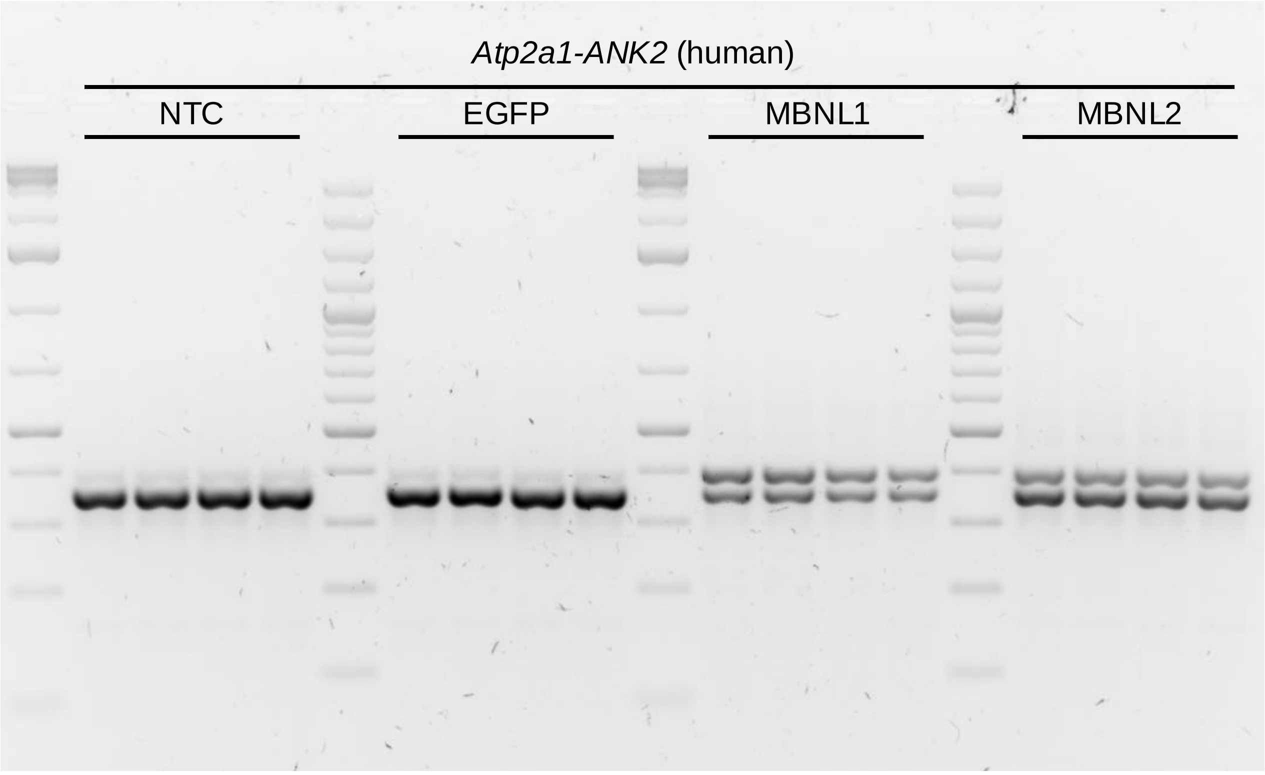

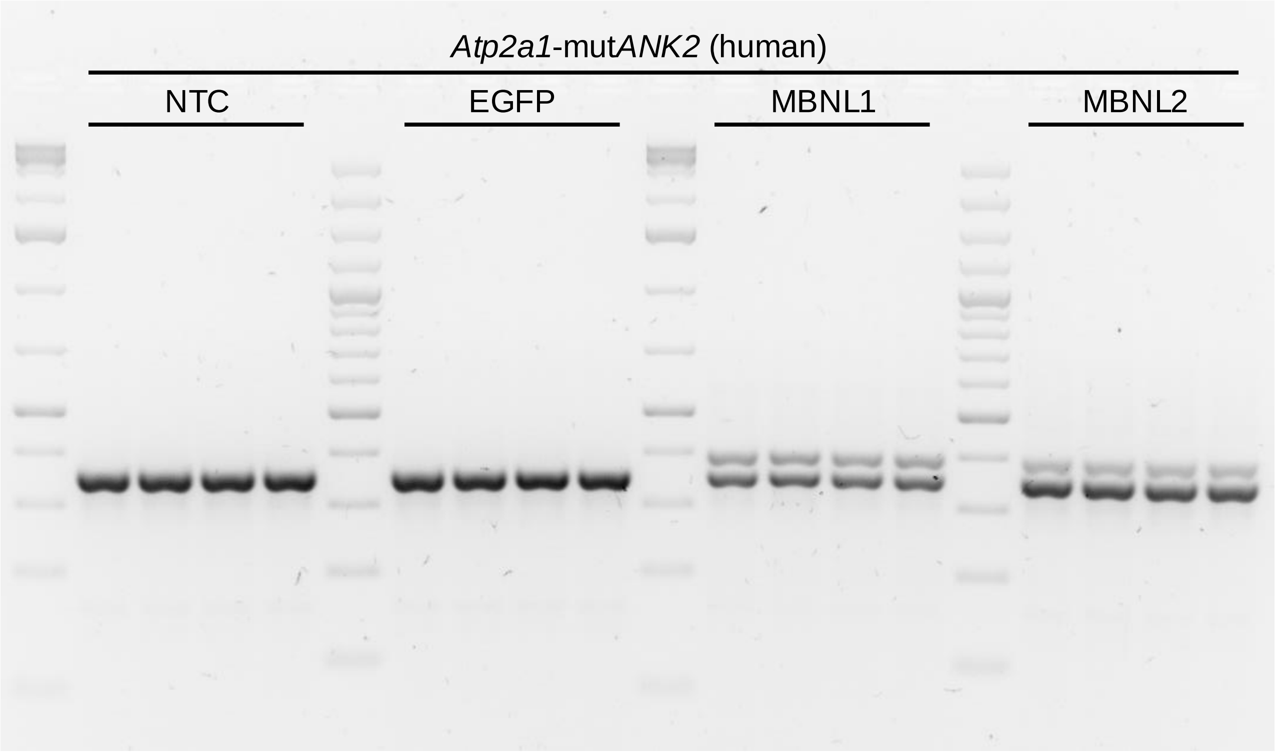

Data for Figure 6g

Non-treated control (NTC)

Picalm miE

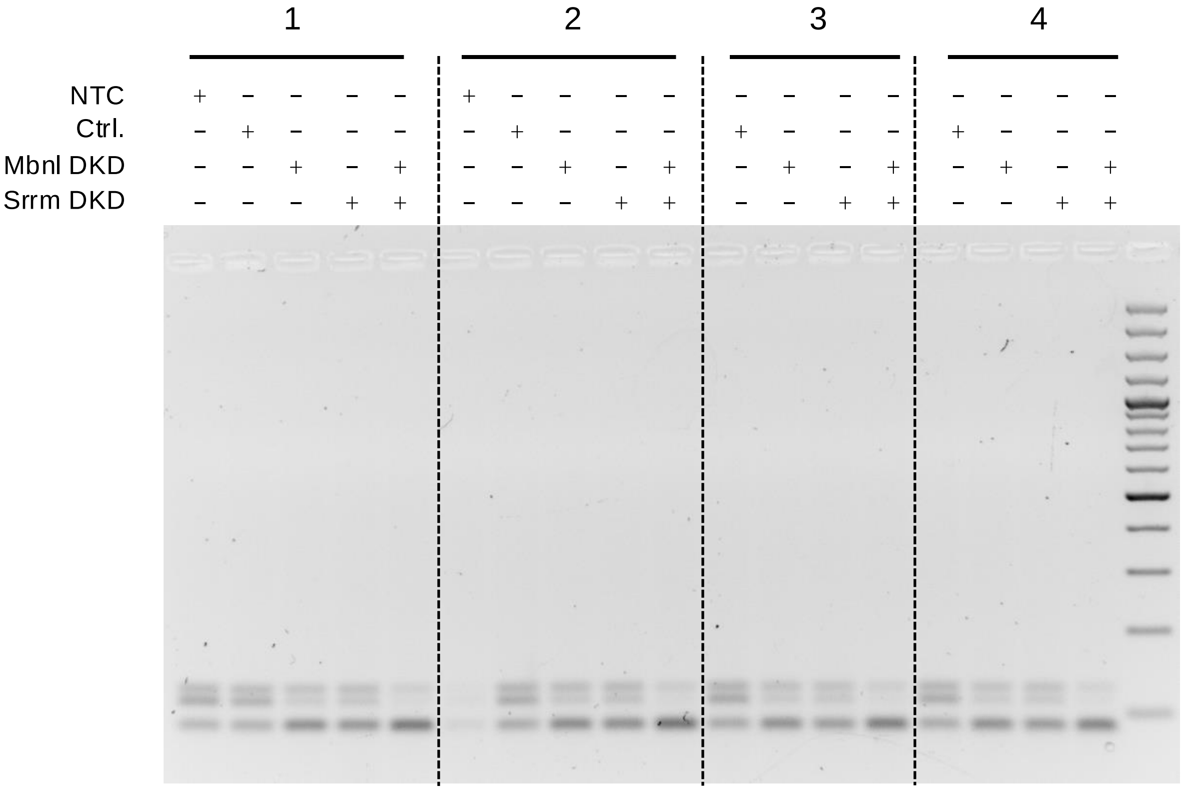

Arfgap miE

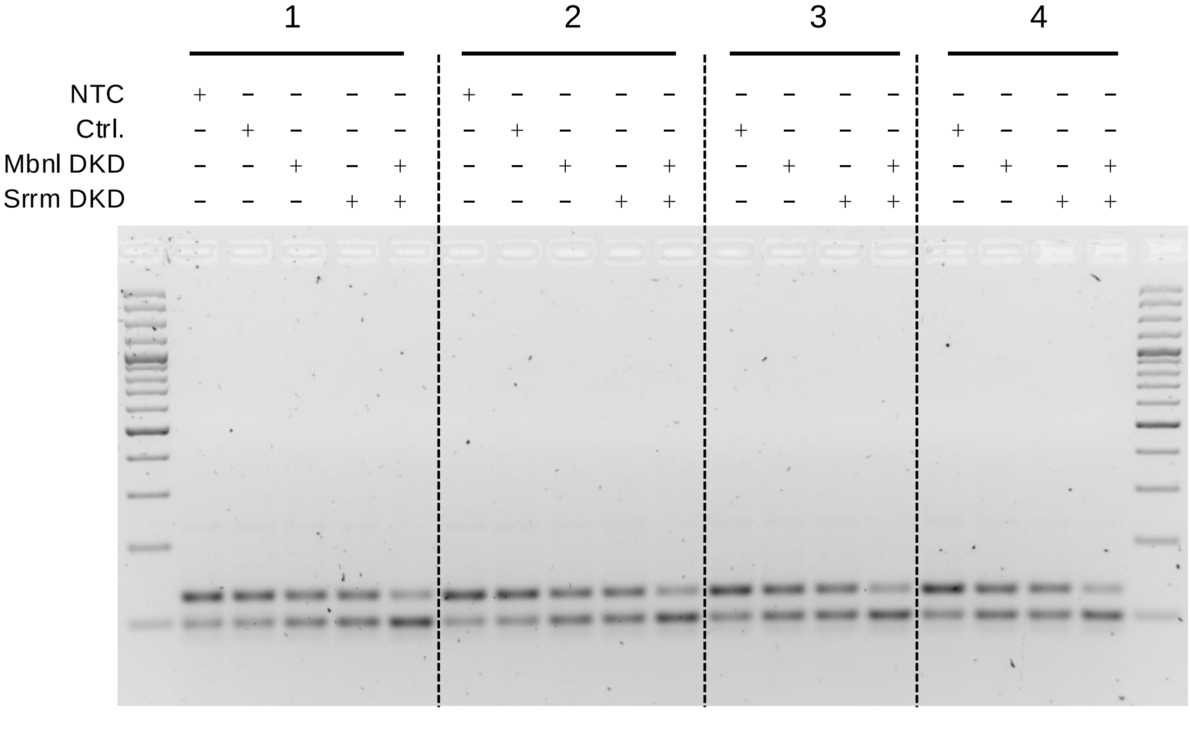

Data for Figure 6g

Non-treated control (NTC)

Cers6 miE

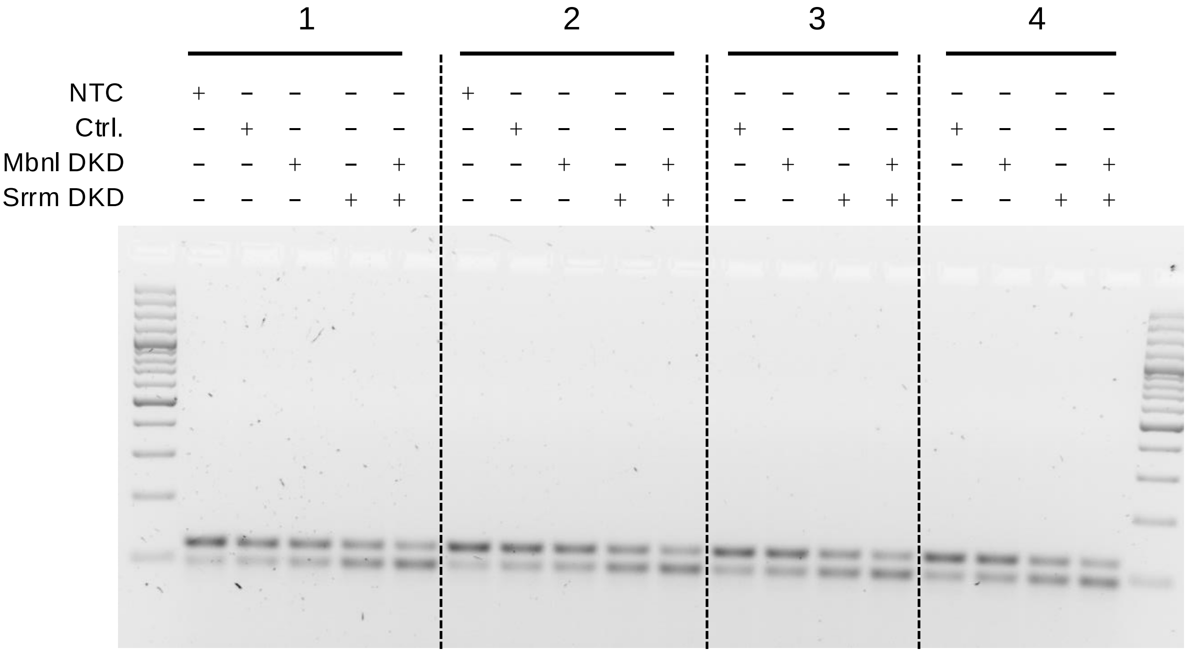

Dst miE

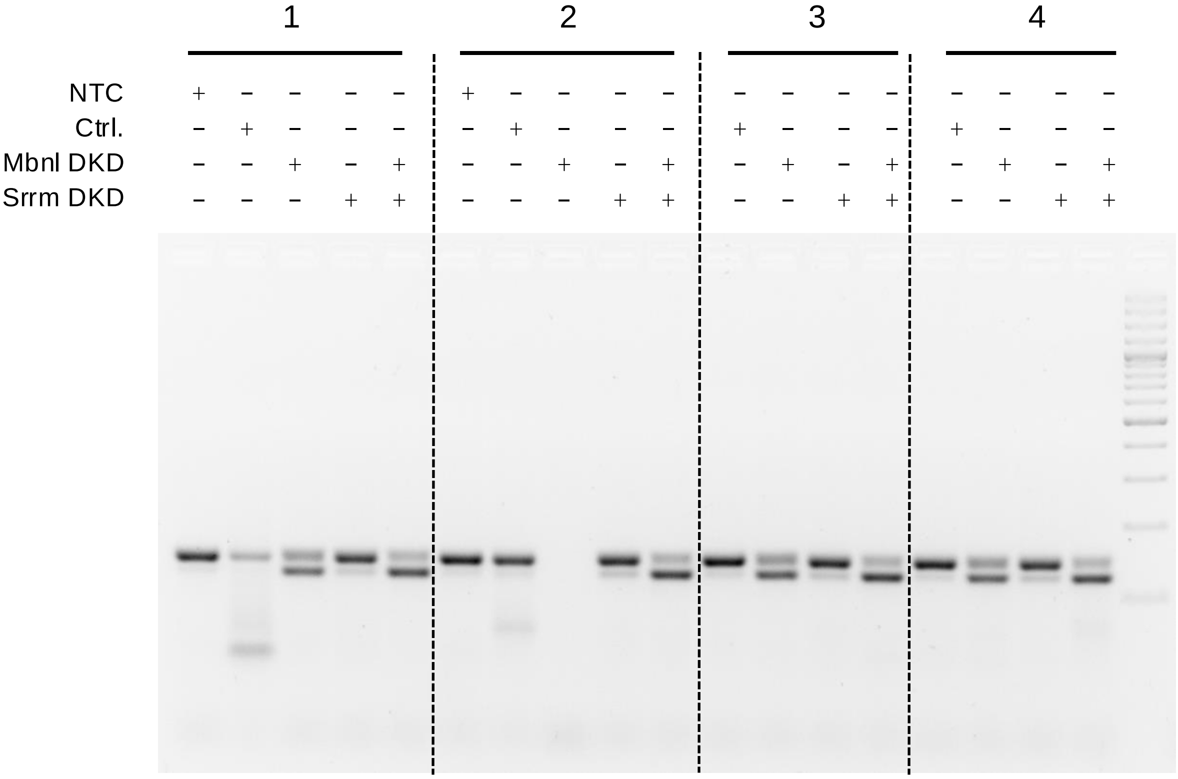

Data for Figure 6g

Non-treated control (NTC)

Clasp1 miE

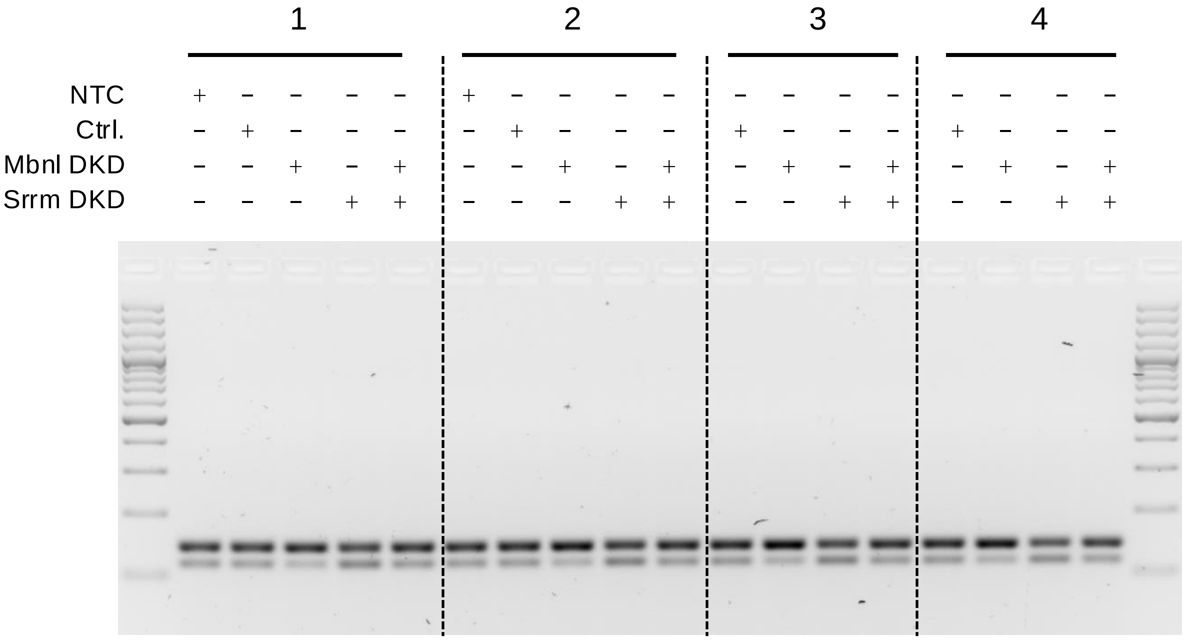

Tjap1 miE

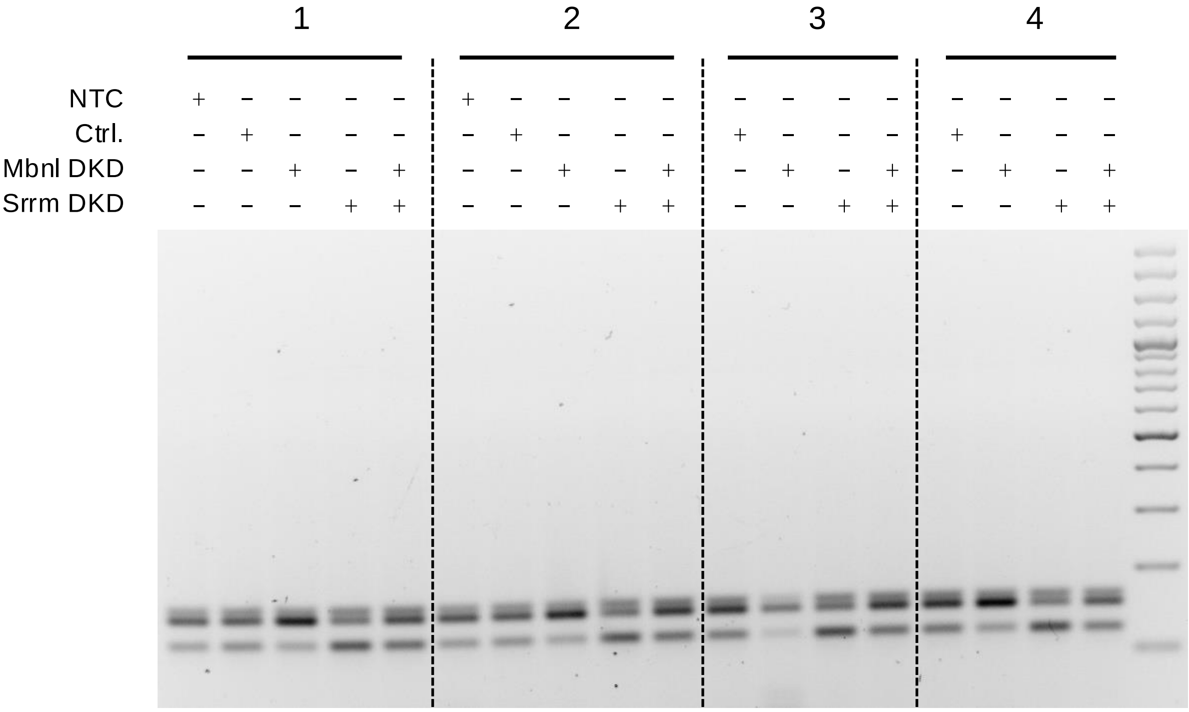

Data for Figure 6g

Non-treated control (NTC)

Arrab miE

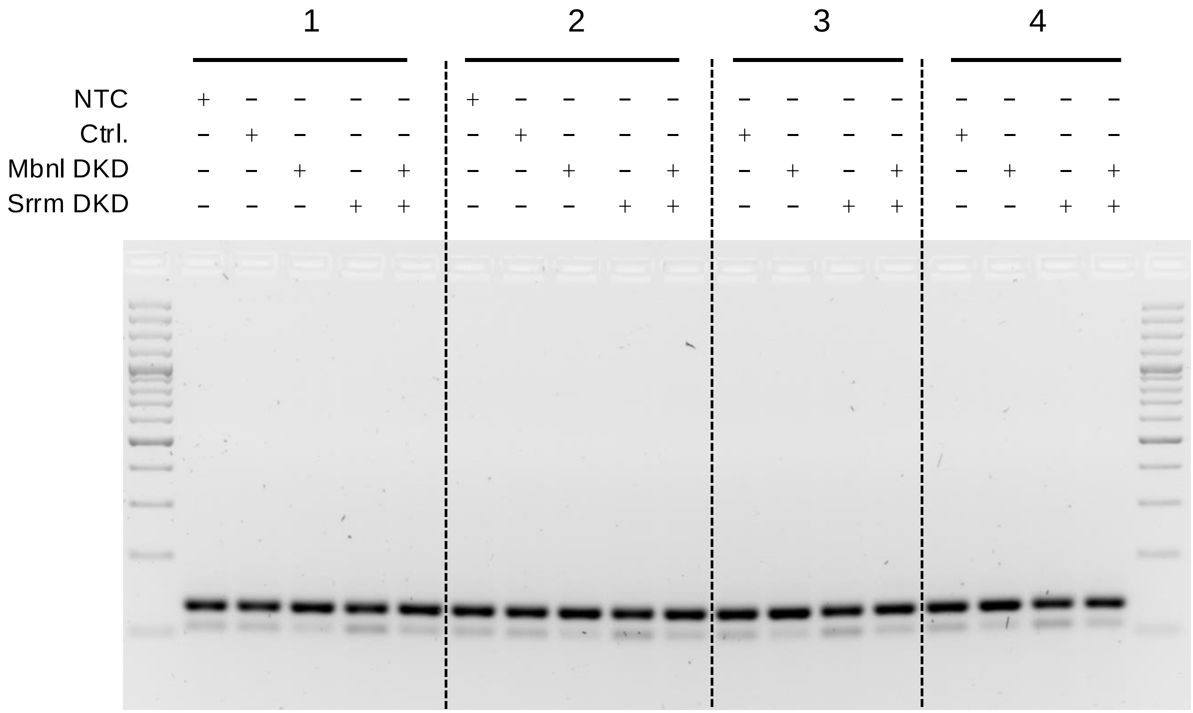

Fxbo25 miE

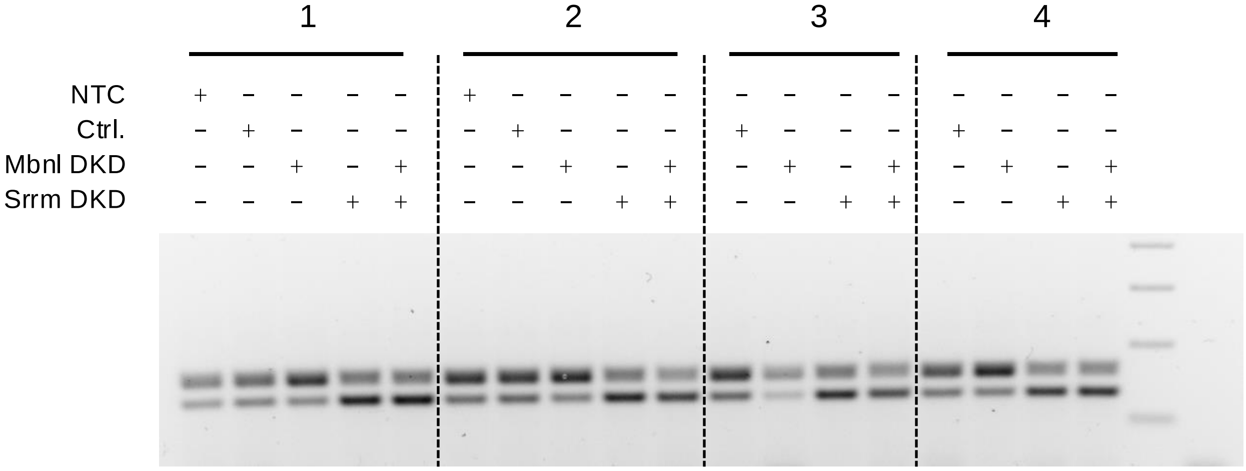

Data for Figure 6h

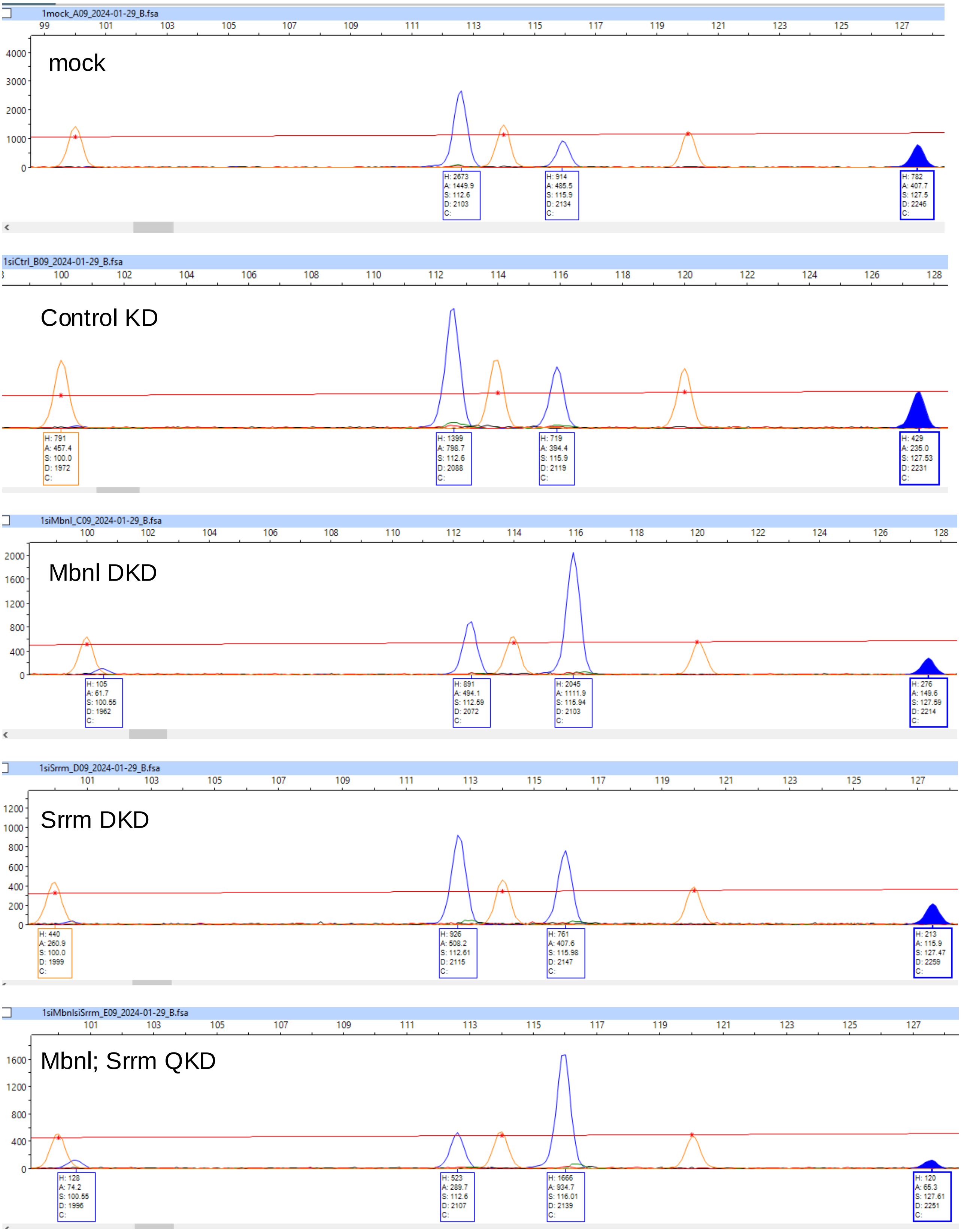

Data for Extended Data Figure 8b

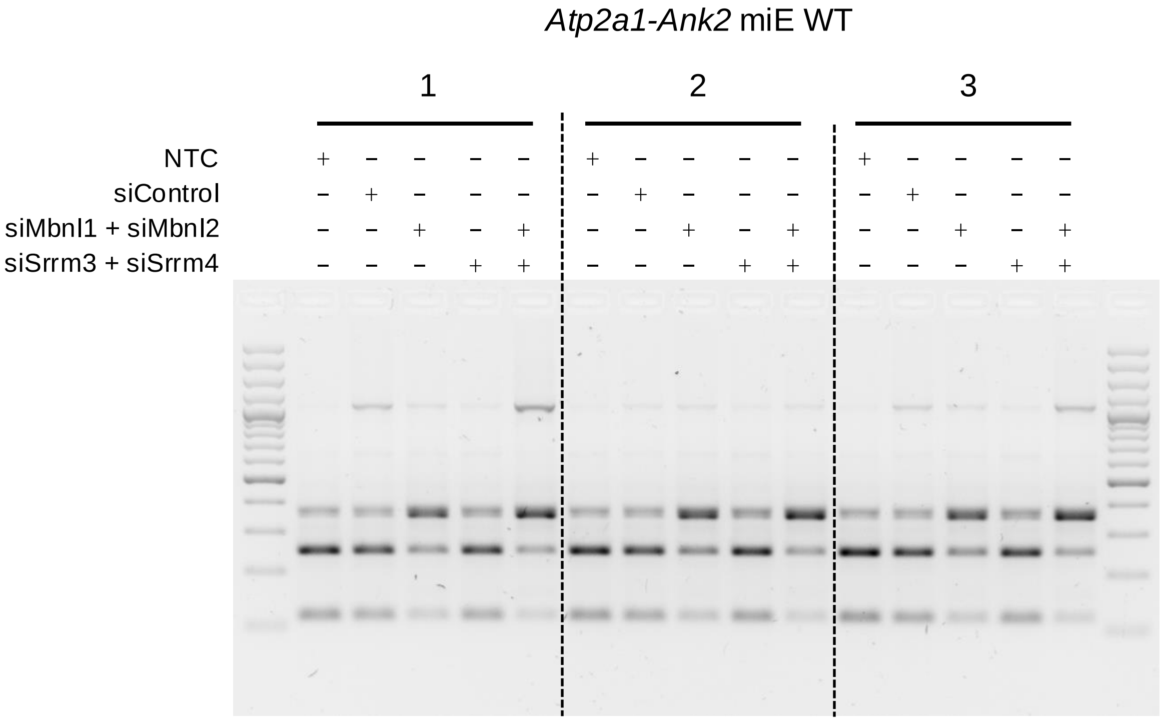

Data for Figure 7f and Extended Data Figure 8c

Atp2a1-Ank2 miE WT

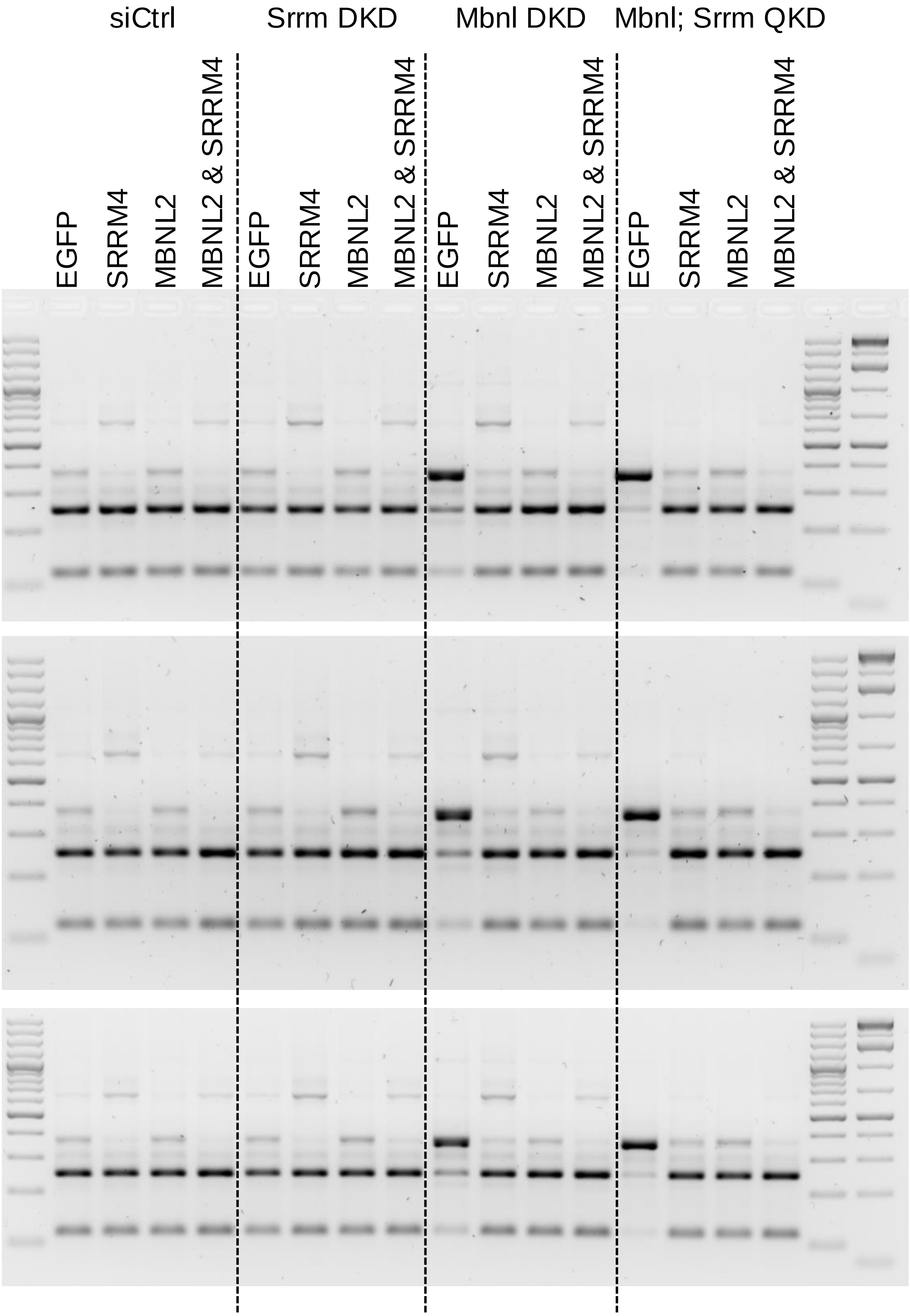

Data for Figure 7f and Extended Data Figure 8c

Atp2a1-Ank2 miE mut. A

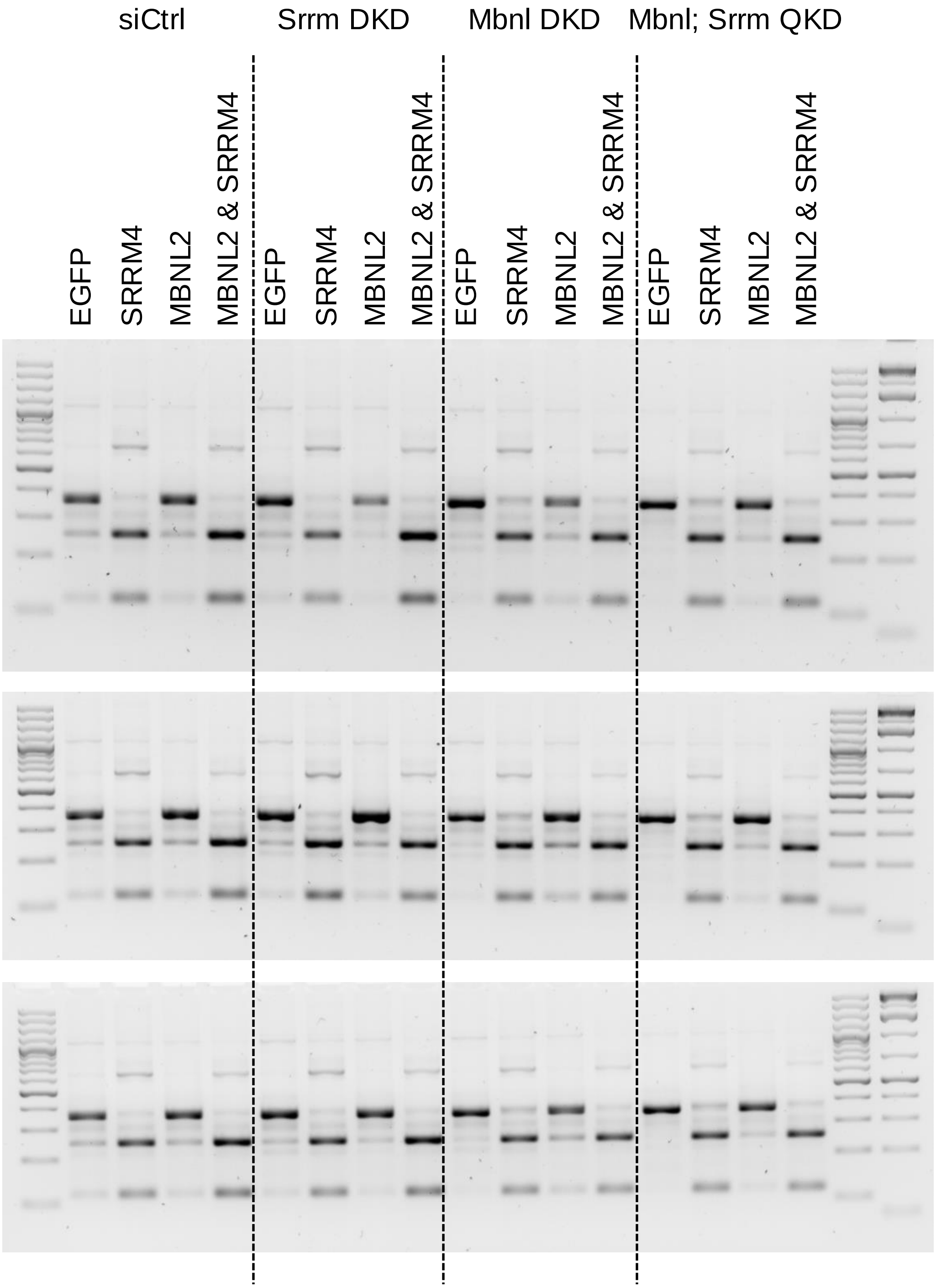

Data for Figure 7f and Extended Data Figure 8c

Atp2a1-Ank2 miE mut. B

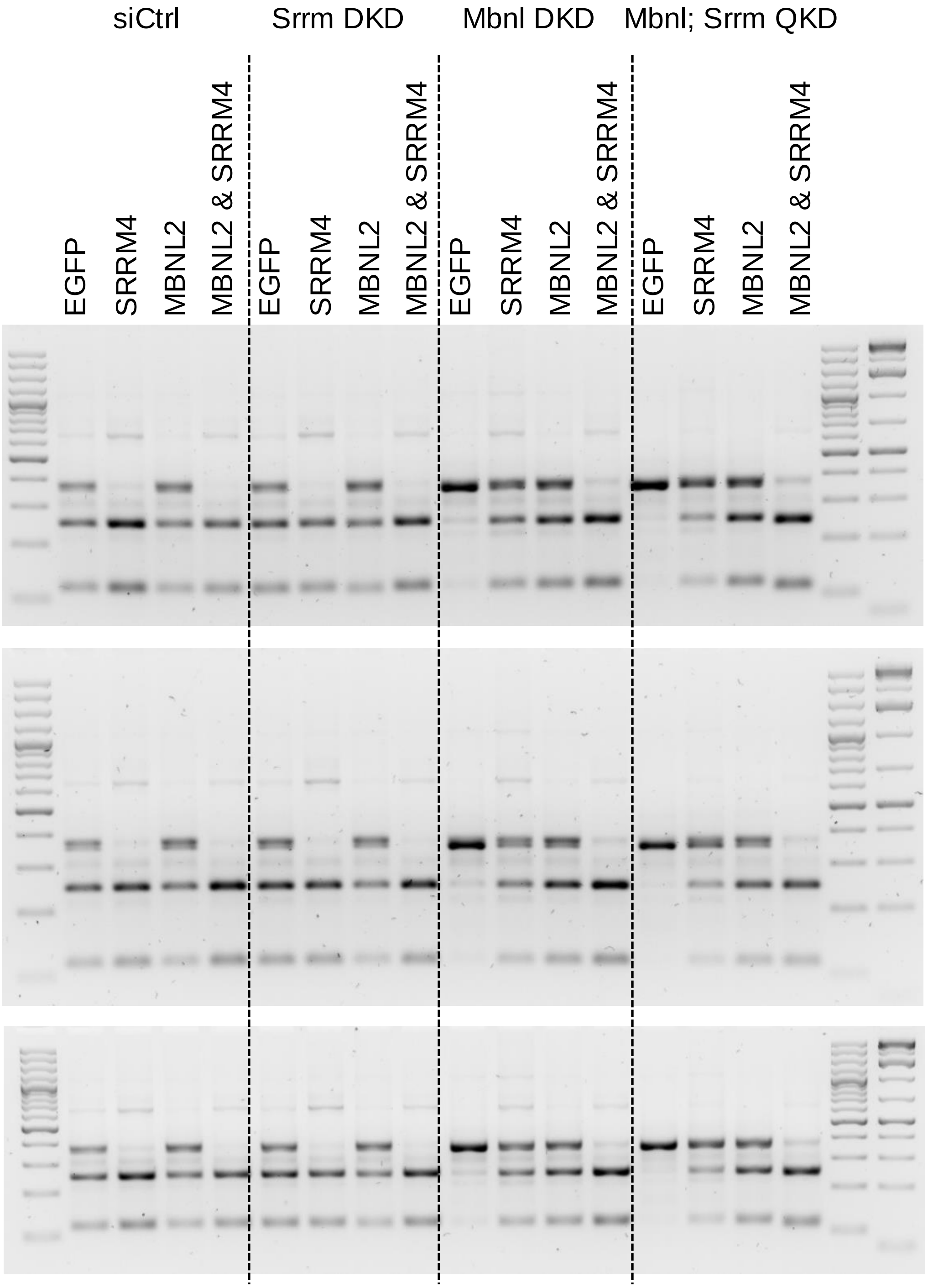

Data for Figure 7f and Extended Data Figure 8c

Atp2a1-Ank2 miE mut. C

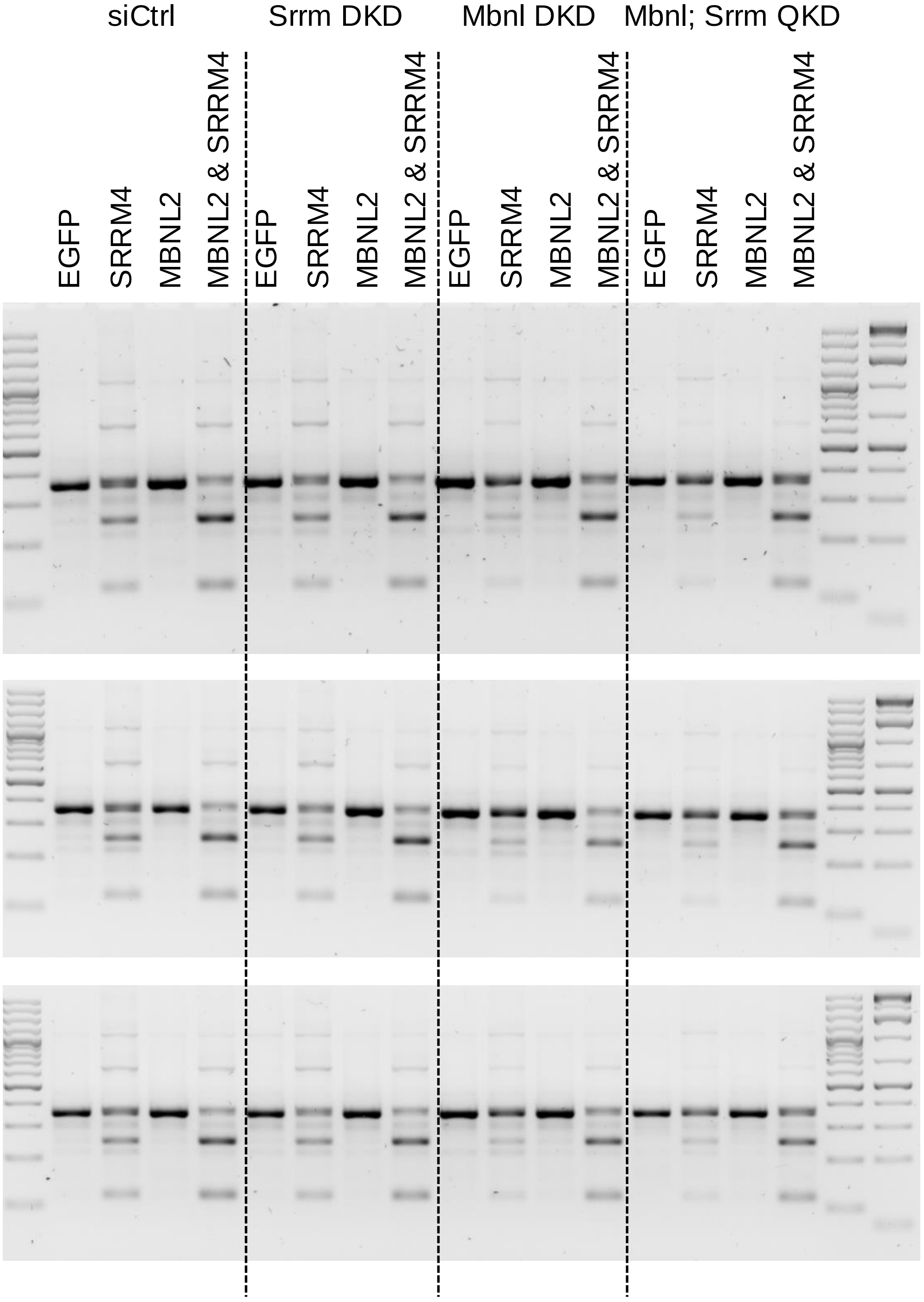

Data for Extended Data Figure 5d

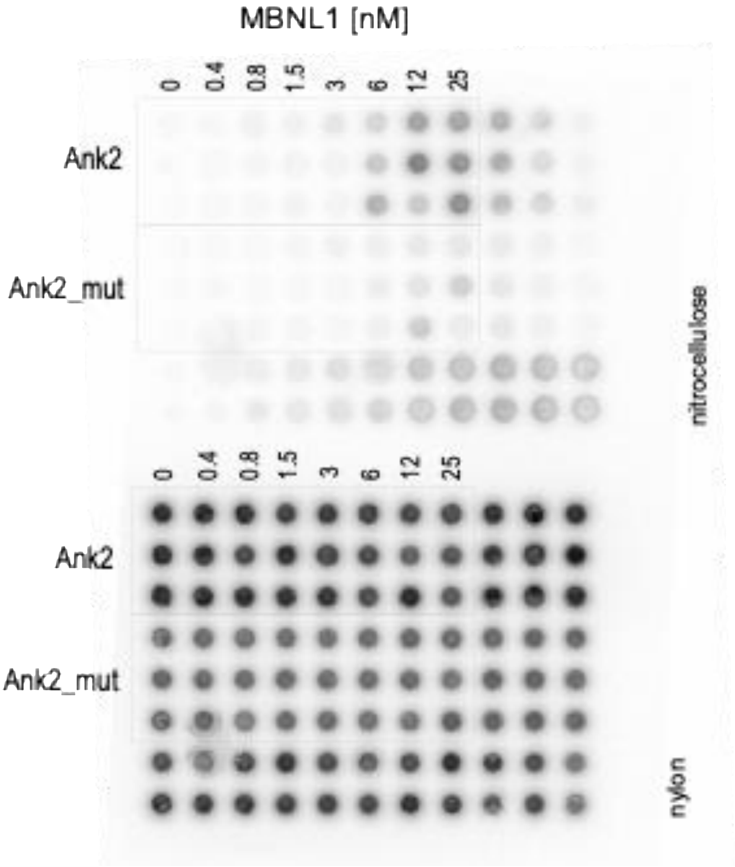

Data for Figure 8j

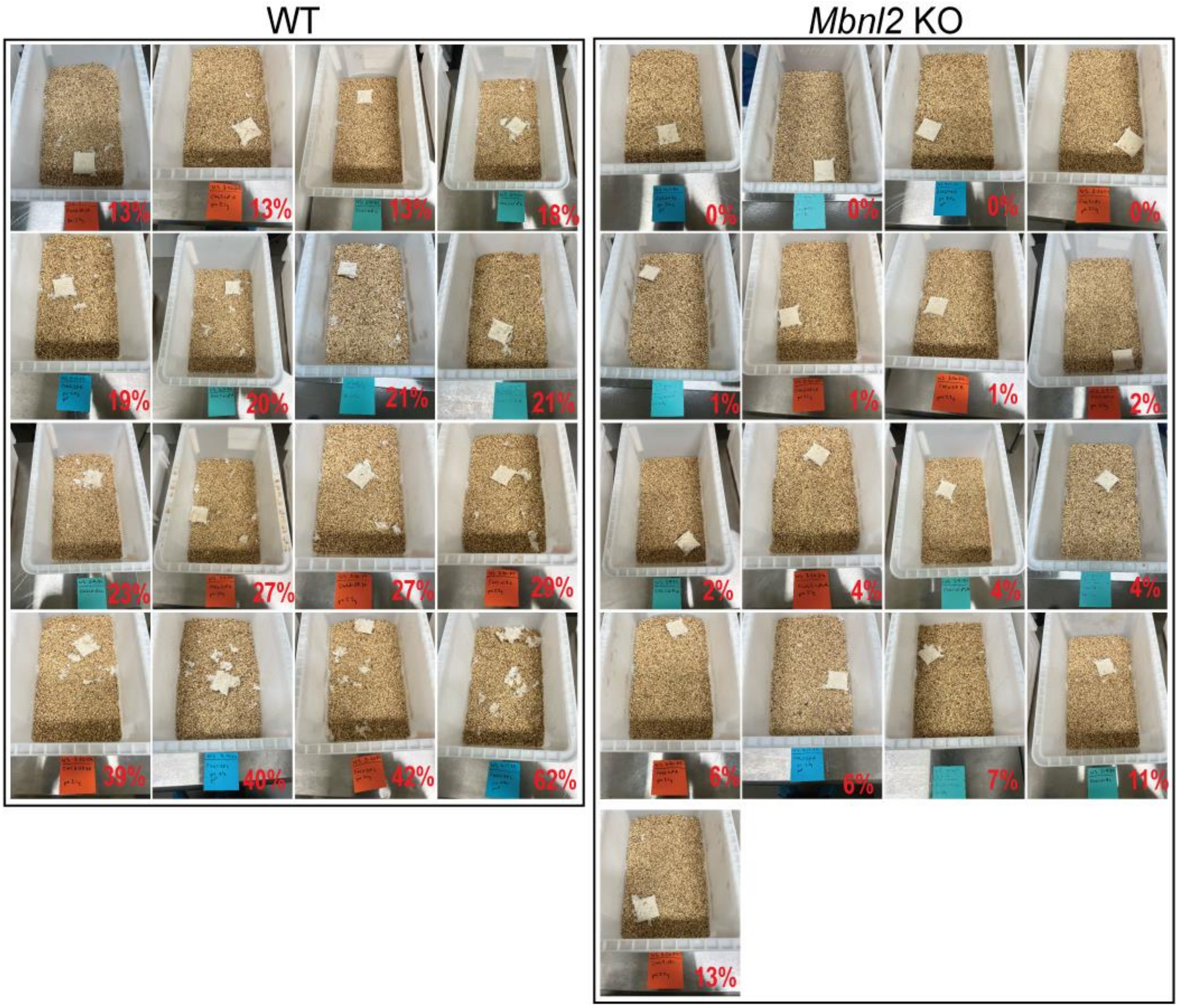

Supplement: Supplementary file 20 — Unprocessed gels. [file 41593_2025_1943_MOESM20_ESM.pdf]
